# Health position paper and redox perspectives on reactive oxygen species as signals and targets of cardioprotection

**DOI:** 10.1016/j.redox.2023.102894

**Published:** 2023-10-06

**Authors:** Gerd Heusch, Ioanna Andreadou, Robert Bell, Edoardo Bertero, Hans-Erik Botker, Sean M. Davidson, James Downey, Philip Eaton, Peter Ferdinandy, Bernard J. Gersh, Mauro Giacca, Derek J. Hausenloy, Borja Ibanez, Thomas Krieg, Christoph Maack, Rainer Schulz, Frank Sellke, Ajay M. Shah, Holger Thiele, Derek M. Yellon, Fabio Di Lisa

**Affiliations:** a Institute for Pathophysiology, West German Heart and Vascular Center, University of Duisburg-Essen, Essen, Germany; b Laboratory of Pharmacology, Faculty of Pharmacy, National and Kapodistrian University of Athens, Athens, Greece; c The Hatter Cardiovascular Institute, University College London, London, United Kingdom; d Chair of Cardiovascular Disease, Department of Internal Medicine and Specialties, University of Genova, Genova, Italy; e Department of Cardiology, Institute for Clinical Medicine, Aarhus University, Aarhus N, Denmark; f Department of Physiology, University of South Alabama, Mobile, AL, USA; g William Harvey Research Institute, Queen Mary University of London, Heart Centre, Charterhouse Square, London, United Kingdom; h Department of Pharmacology and Pharmacotherapy, Semmelweis University, Budapest, Hungary; i Department of Cardiovascular Medicine, Mayo Clinic College of Medicine and Science, Rochester, MN, USA; j School of Cardiovascular and Metabolic Medicine & Sciences, King's College, London, United Kingdom; k Cardiovascular & Metabolic Disorders Program, Duke-National University of Singapore Medical School, National Heart Research Institute Singapore, National Heart Centre, Yong Loo Lin School of Medicine, National University Singapore, Singapore; l Centro Nacional de Investigaciones Cardiovasculares (CNIC), IIS-Fundación Jiménez Díaz University Hospital, and CIBERCV, Madrid, Spain; m Department of Medicine, University of Cambridge, Cambridge, United Kingdom; n Department of Translational Research, Comprehensive Heart Failure Center, University Clinic Würzburg, Würzburg, Germany; o Institute for Physiology, Justus-Liebig -Universität, Giessen, Germany; p Division of Cardiothoracic Surgery, Alpert Medical School of Brown University and Rhode Island Hospital, Providence, RI, USA; q King's College London British Heart Foundation Centre of Excellence, London, United Kingdom; r Heart Center Leipzig at University of Leipzig and Leipzig Heart Science, Leipzig, Germany; s Dipartimento di Scienze Biomediche, Università degli studi di Padova, Padova, Italy; t Pharmahungary Group, Szeged, Hungary

**Keywords:** Cardioprotection, Infarct size, Ischemic conditioning, Mitochondrion, Myocardial ischemia, Myocardial infarction, Reperfusion

## Abstract

The present review summarizes the beneficial and detrimental roles of reactive oxygen species in myocardial ischemia/reperfusion injury and cardioprotection. In the first part, the continued need for cardioprotection beyond that by rapid reperfusion of acute myocardial infarction is emphasized. Then, pathomechanisms of myocardial ischemia/reperfusion to the myocardium and the coronary circulation and the different modes of cell death in myocardial infarction are characterized. Different mechanical and pharmacological interventions to protect the ischemic/reperfused myocardium in elective percutaneous coronary interventions and coronary artery bypass grafting, in acute myocardial infarction and in cardiotoxicity from cancer therapy are detailed. The second part keeps the focus on ROS providing a comprehensive overview of molecular and cellular mechanisms involved in ischemia/reperfusion injury. Starting from mitochondria as the main sources and targets of ROS in ischemic/reperfused myocardium, a complex network of cellular and extracellular processes is discussed, including relationships with Ca^2+^ homeostasis, thiol group redox balance, hydrogen sulfide modulation, cross-talk with NAPDH oxidases, exosomes, cytokines and growth factors. While mechanistic insights are needed to improve our current therapeutic approaches, advancements in knowledge of ROS-mediated processes indicate that detrimental facets of oxidative stress are opposed by ROS requirement for physiological and protective reactions. This inevitable contrast is likely to underlie unsuccessful clinical trials and limits the development of novel cardioprotective interventions simply based upon ROS removal.

## Highlights

• Ischemic heart disease is the most frequent cause of death worldwide.
• Ischemia/reperfusion is the substrate of ischemic heart disease.
• Cardioprotection reduces infarct size and coronary microvascular obstruction.
• ROS are signals of cardioprotection and executors of injury.
• Translation of cardioprotection to clinical benefits has been difficult so far.
• The role of ROS in cardioprotection remains ambivalent.

## Graphical abstract

**Figure:**
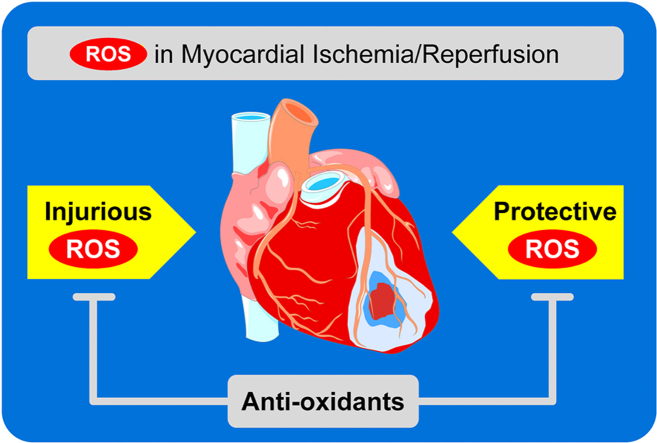

## Abbreviations

Δp: proton motive force
ABCB8: ATP binding cassette protein 8
ADP: adenosine diphosphate
ADSCs: adipose-derived stem cells
AIF: apoptosis initiating factor
Akt: protein kinase B
ALG-CHO: partially oxidized alginate
ALRs: absent in melanoma-2-like receptors
AMI: acute myocardial infarction
AMPK: adenosine monophosphate-activated protein kinase
Apaf-1: apoptotic protease activating factor 1
ATG: autophagy-related gene protein
ATP: adenosine triphosphate
Bax/Bak: Bcl-2 associated X protein/Bcl-2 homologous antagonist
BMP: bone morphogenetic protein
CABG: coronary artery bypass grafting
CaMKII: Ca^2+^/calmodulin-dependent protein kinase II
CCS: chronic coronary syndrome
CMR: cardiac magnetic resonance
CoQ: coenzyme Q
CPB: cardiopulmonary bypass
CPK: creatine phosphokinase
CSE: cystathionine γ-lyase
CTO: chronic total occlusion
cTn: cardiac troponin
CypD: cyclophilin D
DAMP: damage-associated molecular pattern
DIC: dicarboxylate transporter
DCM: diabetic cardiomyopathy
EC: excitation-contraction
ET-1: endothelin-1
EV: extracellular vesicle
FGF-2: fibroblast growth factor-2
GFAT1: glutamine-fructose-6-phosphate transaminase 1
GLUT: glucose transporter
GPCR: G-protein coupled receptor
GPX4: glutathione peroxidase-4
GSDMD: gasdermin-D
GSK3β: glycogen synthase kinase 3β
GSSG: oxidized glutathione
HBP: hexosamine biosynthetic pathway
HGF: hepatocyte growth factor
HIF-1: hypoxia-inducible factor 1
HNO: Nitroxyl
IGF-1R: insulin-like growth factor 1 receptor
IMAC: inner mitochondrial anion channels
IPC: ischemic preconditioning
I/R: ischemia/reperfusion
ISR: integrated stress response
LC3: light chain 3
LV: left ventricular
NLR: leucine-rich repeat
MACE: major adverse cardiovascular events
MAM: mitochondrial-associated membrane
MAO: monoamine oxidases
MAPKs: mitogen-activated protein kinases
MCT1: monocarboxylate transporter 1
MCU: mitochondrial Ca^2+^ uniporter
MIF: migration inhibitory factor
MitoPQ: mito paraquat
MLKL: mixed-lineage kinase domain-like pseudokinase
MYDGF: myeloid-derived growth factor
mTOR: mammalian target of rapamycin
NaHS: sodium hydrosulfide
Na_2_S: sodium sulfide
NCX: Na^+^/Ca^2+^ exchanger
NFkB: nuclear factor kappa B
NHE: Na^+^/H^+^ exchanger
NNT: nicotinamide nucleotide transhydrogenase
NOX: NADPH oxidase
NRF2: nuclear factor erythroid 2 related factor 2
NT-proBNP: N-terminal pro-brain natriuretic peptide
PAMP: pathogen associated molecular pattern
PCI: percutaneous coronary intervention
PCr: phosphocreatine
PI3K: phosphatidylinositol-3-kinase
PMI: peri-procedural myocardial injury
PostC: postconditioning
PP1: protein phosphatase 1
PPCI: primary percutaneous coronary intervention
PRAS40: proline-rich Akt substrate of 40 kDa
PTP: permeability transition pore
Raptor: regulator-associated protein of mTOR
RET: reverse electron transport
RIC: remote ischemic conditioning
RIPK: receptor-interacting serine/threonine-protein kinase
RISK: reperfusion injury salvage kinases
RNS: reactive nitrogen species
ROS: reactive oxygen species
SDH: succinate dehydrogenase
SERCA: sarcoplasmic Ca^2+^ ATPase
sEV: small EVs
SGLT: sodium-glucose -linked transporter
SOD: superoxide dismutase
SPECT: single photon emission computed tomography
SPRC: *S*-propyl-l-cysteine
SR: sarcoplasmic reticulum
SSH: *S*-sulfhydration
STAT3: signal transducer and activator of transcription 3
STEMI: ST segment elevation myocardial infarction
STS: sodium thiosulfate
SUCNR1: succinate receptor 1
TCA: tricarboxylic acid
TGF-β: transforming growth factor-β
TRP: transient receptor potential
TRADD: tumor necrosis factor receptor 1 -associated death domain protein
TRAILR: tumor necrosis factor-related apoptosis inducing ligand receptor
UCPs: uncoupling proteins
UDMI: universal definition of myocardial infarction
ULK-1: Unc-51-like kinase
URL: upper reference limit
VEGF: vascular endothelial growth factor

## 1 Cardioprotection – attenuation of myocardial ischemia/reperfusion injury

### 1.1 Cardioprotection - what does it mean?

Cardioprotection has been defined as “all mechanisms and means that contribute to the preservation of the heart by reducing or even preventing myocardial damage” [1]. This is a very broad definition which encompasses primary and secondary prevention, non-pharmacological and pharmacological conservative therapy, and interventional and surgical invasive therapy of all cardiac diseases from arrhythmias to coronary artery disease to valve disease and finally heart failure. While this definition is reasonable, it is conceptually very broad and pragmatically not very helpful. In a stricter sense, cardioprotection is therefore defined as prevention or reduction of myocardial injury from myocardial ischemia/reperfusion (I/R) [2]. Myocardial I/R is the pathophysiological substrate of ischemic heart disease which is still the most frequent cause of death worldwide [3]. In this sense, cardioprotection comprises the reduction of I/R injury not only to cardiomyocytes, but also to other cellular compartments, notably the coronary circulation. Also, cardioprotection may not only refer to reduction of acute injury to cardiomyocytes and the coronary circulation, i.e., infarct size and coronary microvascular obstruction, but also to inflammation, healing, repair and remodeling after such myocardial I/R injury, in particular when clinical outcome, notably mortality and heart failure, after follow-up is considered as endpoint. There is in fact a compelling medical need to develop therapies that protect the heart from cardiomyocyte loss. Death of cardiomyocytes is dramatic after myocardial infarction, when it can affect up to 25% of the approximately 4 billion cells in the left ventricle [4], but also accompanies most other cardiac conditions. These include disorders of cardiac overload (such as hypertension [5] or aortic stenosis [6]), viral myocarditis [7], Takotsubo syndrome [8] and peri-partum cardiomyopathy [9]. Cardiomyocyte death also accompanies virtually all forms of inherited cardiomyopathies, including Duchenne muscular dystrophy [10], Danon [11] and desmin [12] cardiomyopathies. There is evidence of cardiomyocyte loss in both dilated and hypertrophic cardiomyopathy [13] and in arrhythmogenic cardiomyopathy [14]. Finally, cardiac cell loss occurs during perioperative myocardial injury and reperfusion [15] and following cancer therapy, in particular using anthracyclines [16].

### 1.2 The clinical need for cardioprotection

**Fig. 1:**
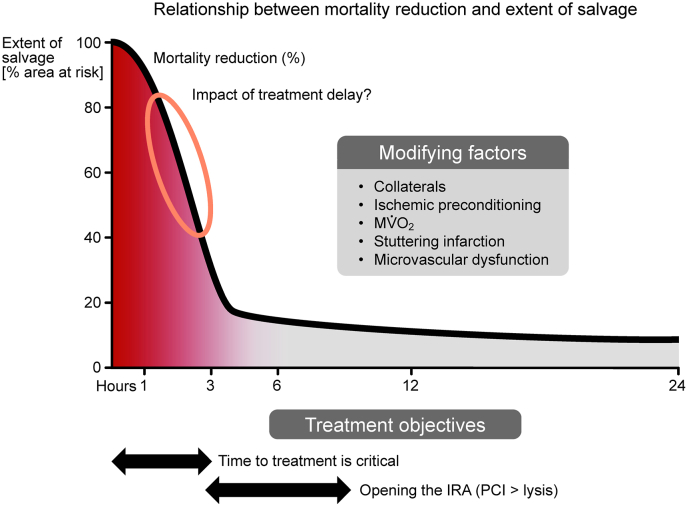
Time course of myocardial salvage. With short duration of ischemia, reperfusion alone salvages almost all myocardium at risk. With a long duration of ischemia and late reperfusion, there remains little myocardium to be salvaged. There is a narrow time window for cardioprotective interventions beyond reperfusion (encircled). The time scale as such is influenced by a number of intervening variables (inserted box). IRA, infarct-related artery. From [25].

Cardiovascular disease is still the greatest health burden worldwide, and ischemic heart disease is still the most frequent cause of death worldwide [17]. The mortality in the first year following an acute myocardial infarction (AMI) remains at 15–21% in large European registries [18,19]. The modern era of reperfusion therapy, propelled in part by the seminal studies of Ross et al. [20,21] and Reimer and Jennings [22,23], demonstrating infarct size reduction by timely reperfusion and illustrating the gradual progression of necrosis after coronary occlusion in dogs, has resulted in a therapeutic revolution. Subsequent experimental studies demonstrated the significant benefits of standard reperfusion therapy on myocardial salvage but only an incremental benefit from the addition of physical interventions and pharmacologic agents which limit reperfusion injury [24]. A hypothetical construct illustrating the relationship between mortality reduction, myocardial salvage, and the duration of ischemia prior to reperfusion emphasized the narrow time window in which restoration of reperfusion could achieve significant salvage [25] (Fig. 1). This relationship between the slope of the curve and the extent of salvage could be altered by modifying factors such as ischemic conditioning interventions, the extent of the collateral circulation, myocardial oxygen consumption, stuttering infarction and microvascular dysfunction. A subsequent meta-analysis of 10 randomized control trials comprising 2632 patients emphasized the critical importance of infarct size for one-year prognosis after primary percutaneous coronary intervention (PPCI) [26].

In regard to the blueprint for optimal care of the patients with ST segment elevation myocardial infarction (STEMI), we know what to do and the process begins with the establishment of a treatment protocol and a network [27]. Nonetheless there are many variables than can have a major impact upon the outcome, from delivery of reperfusion therapy to as many people and as quickly as possible. These include physical constraints such as geography, travel distance, weather, and access to ambulance transport as well as the coordination and availability of services both institutionally and regionally [28]. Despite a widespread understanding of the pivotal importance of time-to-reperfusion on the extent of myocardial salvage and the relationship between final infarct size and clinical outcomes the delivery of reperfusion therapy is subject to limitations and as such there remains an ongoing need for cardioprotection [29]. Several recent studies have drawn attention to difficulties in adhering to guidelines-directed times of treatment and the detrimental impact of treatment delay upon clinical outcomes [30,31]. A large study from a USA National Cardiovascular Data Registry of 22481 patients undergoing transfer to a PPCI-capable hospital with the median estimated inter-hospital drive time of 57 min emphasized that the majority had a first door-to-balloon time much longer than the guideline-recommended goal of less than or equal to 120 min, and the use of fibrinolytics as a pharmaco-invasive strategy in patients with longer drive times was disappointingly low [30]. These studies in high-income countries emphasize the logistical constraints imposed by geography, weather, and socio-economic disparities in healthcare. When these issues are faced in the context of low-income countries like Sub-Saharan Africa, the magnitude of the task and the obstacles to optimal reperfusion therapy are exceptionally challenging, and potentially the role of the cardioprotection is much greater [32] in that the cardioprotective effects of the pharmacological and non-pharmacological approaches may increase with the duration of ischemia. An example of the dramatic adverse impact of treatment delay on the outcomes of an invasive strategy for STEMI was highlighted by a prospective registry of patients in 55 interventional centers primarily in the United Kingdom and Europe during the initial days of the COVID pandemic [33].

From the perspective of cardioprotection, the road from the experimental laboratory to the clinical arena has been difficult [34,35]. Although the agenda for both pharmacologic and non-pharmacologic approaches has been extensive, the results overall have been unimpressive. The pathophysiology of I/R injury and microvascular dysfunction is complex and involves both cardiomyocyte and coronary vascular compartments [36]. Multiple molecular pathways are involved, and what is encouraging is the plethora of potential therapeutic targets, but a theoretical disadvantage is the potential for redundancy and alternative pathways. Only one clinical outcome trial (RIC-STEMI) has achieved its primary endpoint of reduced mortality and hospitalization for heart failure [37] although some studies have demonstrated positive signals in regard to surrogate endpoints including cardiac magnetic resonance (CMR) measurements of infarct size and microvascular function [2].

Obviously, there are differences between the experimental models and clinical studies [35]. Nonetheless, we believe we need to look further into the changing natural and unnatural history of STEMI and its impact on hard clinical endpoints. One example is provided by recent trials of remote ischemic conditioning (RIC). The basic science is well studied, logical and indeed exciting and cell-signaling pathways have been well defined as has evidence for multi-organ protection during cardiac and non-cardiac surgery [[38], [39], [40]]. Surrogate endpoints such as ST segment resolution, creatine phosphokinase (CPK) and cardiac troponin (cTn) levels, CMR estimates of infarct size, salvage and myocardial edema, N-terminal pro-brain natriuretic peptide (NT-proBNP) and admissions for heart failure have been positive in favor of RIC. This led to the large CONDI 2-ERIC/PPCI trial of 4637 patients which was neutral in regard to the 12-month outcome of cardiac death or heart failure hospitalization (9.4% with RIC vs. 8.6% in controls p = 0.32) [41]. This well-conducted trial was in a way a victim of its own success and provides an impressive example of what can be achieved in high-income countries in the contemporary era of PPCI. Cardiovascular mortality was less than 3%, and 96% of patients had no signs or symptoms of heart failure. This was an excellent trial, but it is easy to understand that irrespective of whether RIC improves myocardial salvage, the magnitude of the impact of this intervention in this population with a very low event rate is probably insufficient to change prognosis [28].

Nonetheless, there remains an unmet need for adjunctive cardioprotection, and particularly in sicker patients with hemodynamic complications, i.e. higher Killip classes, and less than optimal reperfusion and in patients in low- and middle-income countries among whom delayed presentation to hospital is frequent, access to invasive care is limited and there is a high incidence of untreated associated comorbidities. In this setting, RIC remains a highly promising, innovative, and biologically plausible strategy which needs to be tested in different clinical settings. Much needed trials and ongoing trials include the RIP-HIGH trial in patients with Killip class 2 or higher in Germany (NCT 04844931) and the RIC-AFRICA trial in South Africa, Sudan, Uganda, and Kenya [42].

Fig. 1 illustrates the critical interactions between the duration of ischemia and time to reperfusion and emphasizes that the window of opportunity for an intervention to exert a clinically significant prognostic impact is limited. For patients treated early in the course of AMI, it will be difficult to demonstrate a prognostic difference, and if treated late on the “flat” part of the curve it will be too late to make a difference. Perhaps ischemic conditioning can move the graph to the right and widen the window. The concepts are sound, the need for cardioprotection remains in many different clinical settings but the logistical constraints are formidable [43]. Ongoing trials will hopefully resolve many unanswered questions.

### 1.3 Cardiomyocyte ischemia/reperfusion injury – pathophysiology and targets for cardioprotection

**Fig. 2:**
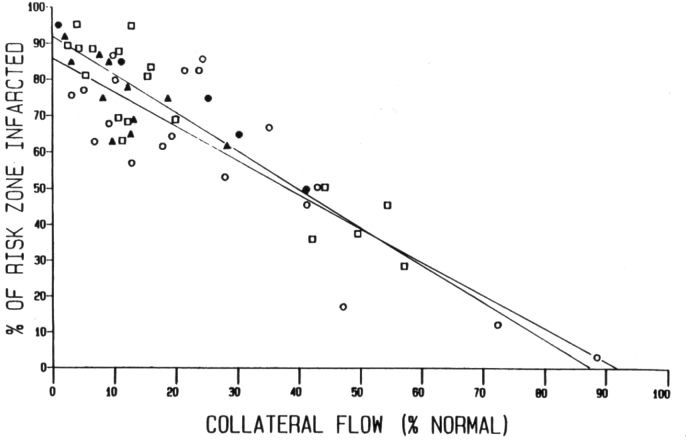
Inverse relationship between infarct size and ischemic myocardial blood flow. Open symbols depict infarct sizes in dog hearts following 24 (circles) or 48 h (squares) of coronary artery branch occlusion. Closed symbols are from published studies by Reimer and Jennings with 96 h (triangles) or 4 h (circles) of ischemia. Infarct size varied widely because of a high degree of variability in collateral flow among the canine hearts [329].

The myocardium is elegantly designed to deal with cyclical transient hypoxia of short duration that occurs with high intra-myocardial pressure during the systolic phase of the cardiac cycle. However, prolonged myocardial ischemia over many tens of minutes is non-physiological, for which there is no evolutionary adaptation in the mammalian heart. Four factors effectively define the final infarct size after coronary occlusion. First is the anatomical location of the coronary occlusion. Proximal left coronary occlusion is associated with both large myocardial infarct size and high lethality if left untreated. Second is the presence or absence of collateralization: the greater the collateralization of the ischemic zone, the more resistant the myocardium will be to coronary occlusion (Fig. 2). Third is the severity of the coronary occlusion: critical myocardial ischemia may occur even when there is some residual coronary flow (Thrombolysis in Myocardial Infarction (TIMI) flow greater than 0). And fourth, the ischemic time before restoration of coronary flow to the ischemic myocardium. Without reperfusion, the ischemic zone will progress to irreversible death, but reperfusion itself is not a benign process [2,36,44]. Up to 50% of the myocardial injury that occurs following reperfusion may be secondary to the reperfusion process itself [45]. Reperfusion can be divided temporally into three phases: (1) hyper-acute (the first 10–15 min), (2) acute (first 24 h) and (3) subacute (first 3 days). The hyper-acute phase of I/R injury is key to the final injury: the smaller the initial reperfusion injury, the less there will be of consequent acute and subacute inflammatory pathways.

Classically, myocardial infarction is described as unregulated cellular degradation and hypercontracture, that has disastrous consequences upon adjoining cells. Classically, necrosis is considered as a non-regulated form of cellular death, and this view is still true except for necroptosis which is described separately below. Contraction band necrosis will drive up local intramyocardial pressure, which in combination with myocyte swelling, will impact local coronary perfusion within the ischemic zone, serving to further intensify local myocardial ischemia. Concomitantly, necrotic cellular rupture will release cellular proteins into the extracellular milieu to propagate cell death via receptor-mediated pathways. However, necrotic cell death is not the sole mechanism of cellular injury response, but rather one bookend of cell damage that ranges from reversible injury through to unregulated necrotic cell death. The ischemic myocardium has a gradient of injury, dominated by necrosis at its core. The necrotic core is surrounded by a mantle of cells undergoing a stress response that may lead to a programmed form of cell death, with or without inflammatory consequences, intended to facilitate tissue repair. The pathophysiology of AMI is thus characterized by repurposed physiological processes designed to deal with cellular stress, trauma and pathogen exposure.

#### 1.3.1 Ischemic stress signaling-enzymatic and mitochondrial

Following acute coronary occlusion, the myocardium is subjected initially to hypoxia and recruitment of acute stress responses that are initially cardioprotective. Hypoxia-inducible factor 1 (HIF-1) is the prototypical sensor of hypoxia that has both acute responses through promoting glycolytic and anaerobic metabolic changes that assist to reduce mitochondrial stress [46,47] and more chronically, differential gene expression through nuclear factor kappa B (NFkB). Another hypoxia/metabolic sensor is adenosine monophosphate-activated protein kinase (AMPK), which responds primarily to the prevailing AMP:ATP ratio but also to the intracellular calcium concentration via calcium calmodulin kinase kinase 2 (CaMKK2) [48]. There is cross -talk between AMPK and HIF-1 pathways that promote gene transcription of glucose transport via glucose transporters (GLUT) [49] and sodium-glucose -linked transporter 1 (SGLT1) [50], both of which are beneficial as the myocardium switches towards glucose metabolism. Such changes would be highly beneficial in the border zone of the ischemic myocardium, where hypoxia likely persists rather than progressing through to necrosis as found towards the center of the ischemic zone. As ischemic duration increases, more maladaptive processes will occur, through the generation of reactive oxygen species (ROS), either from enzyme systems such as NADPH oxidase (NOX) [51] or from mitochondria, such as reversed mitochondrial electron transport following succinate accumulation during ischemia, leading to ROS generation via complex I [52]. In addition to being an important source of ROS, the mitochondria are particularly vulnerable to ischemic stress, leading to organelle swelling and rupture and mediated through ROS- and mitochondrial calcium-triggered opening of the mitochondrial permeability transition pore (PTP). Rupture of the outer mitochondrial membrane then releases cytochrome-c into the cytosol. Mitochondrial fission may be an important step in the acute management of the hypoxic/ischemic stress response, breaking up mitochondrial tubule networks within cardiomyocytes to prevent rapid progression of mitochondrial rupture throughout a whole chain of coupled mitochondria [53]. Mitochondrial release of cytochrome-c is a key step in apoptotic cell signaling. Once wholesale cellular homeostasis collapses and cytosolic membranes are ruptured, the release of cellular contents into the extracellular milieu are recognized by adjacent cells as damage-associated molecular patterns (DAMPs) – that in turn may activate cell death pathways, apoptosis and pyroptosis.

Targeting these initiators of the hyperacute I/R response may therefore provide useful targets for promoting protection against I/R injury, such has adaptive signaling activation through HIF-1 α or AMPK [54] or though inhibiting injury processes, such as ROS formation from enzymatic sources and mitochondria [55], or through metabolic modification to prevent succinate accumulation and mitochondrial ROS generation [52]. Prevention of PTP formation is also a key final pathway to cellular death, with close interaction with programmed cell death pathways, and cyclophilin-D is one regulatory target to prevent PTP opening [56]. However, a multimodal approach may be more appropriate to optimize cellular survival, by regulating down-stream programmed forms of cellular injury [57].

#### 1.3.2 Cell-stress response and cell-death mechanisms

**Fig. 3:**
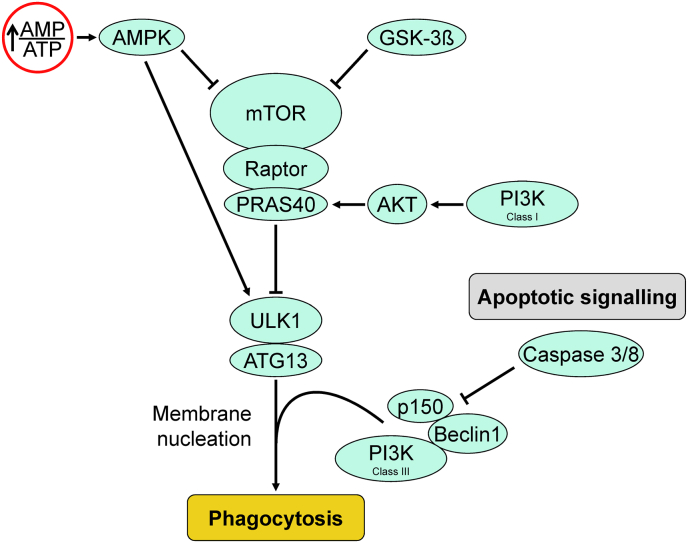
**Autophagic signaling cascade.** The autophagic process is predominantly regulated by the Unc-51-like kinase-1 (ULK1) and is facilitated through phosphorylation via class III PI3K and Beclin-1. Mammalian target of rapamycin (mTOR) inhibits autophagy and is itself inhibited by adenosine monophosphate kinase (AMPK) and glycogen synthase kinase 3β (GSK3β). PRAS40 = proline-rich Akt (protein kinase B) substrate of 40 kDa. ATG13 = autophagy related gene protein 13.

***Autophagy*** is a component of cellular homeostasis and maintenance, recycling degraded proteins and cytoplasmic organelles, performing cellular hygiene tasks. Following an ischemic insult, autophagy is initiated through the inhibition of mammalian target of rapamycin (mTOR), which-in turn-is inhibited through phosphorylation by AMPK and glycogen synthase kinase 3β (GSK3β). Inhibition of mTOR releases its brake on the autophagic signal transduction and activates autophagy [58]. Downstream signaling leads to formation of double-membrane autophagic vesicles. Breakdown of the autophagic vesicles is through recruitment of lysosomal activity and forms complexes with beclin-1 and autophagy-related gene protein (ATG) and lead to P62 degradation (Fig. 3). This process is largely anti-inflammatory, safely compartmentalizing and removing damaged proteins and organelles such as mitochondria, before they activate cell death pathways (for example with cytochrome-c release and activation of the apoptosome through caspase-8 and apoptotic protease activating factor 1 (Apaf-1)) [2,59].

Autophagy, however, can be a double-edged sword. Excessive autophagy will lead to unnecessary self-digestion and cell death [60]. Vice versa, autophagic inhibition, either by apoptotic or necroptotic signaling (Fig. 3) or increased lysosomal zinc during I/R [61] may commit a potentially reversibly injured cell to an untimely programmed cell death. Thus the challenge is to optimize autophagic activity to balance the benefit of damaged organelle and protein removal against excessive premature removal that endangers the survival of a recoverable cell. Autophagy is a homeostatic mechanism to remove and recycle senescent cellular material and contributes to infarction only when excessive (autosis) [62].

**Fig. 4:**
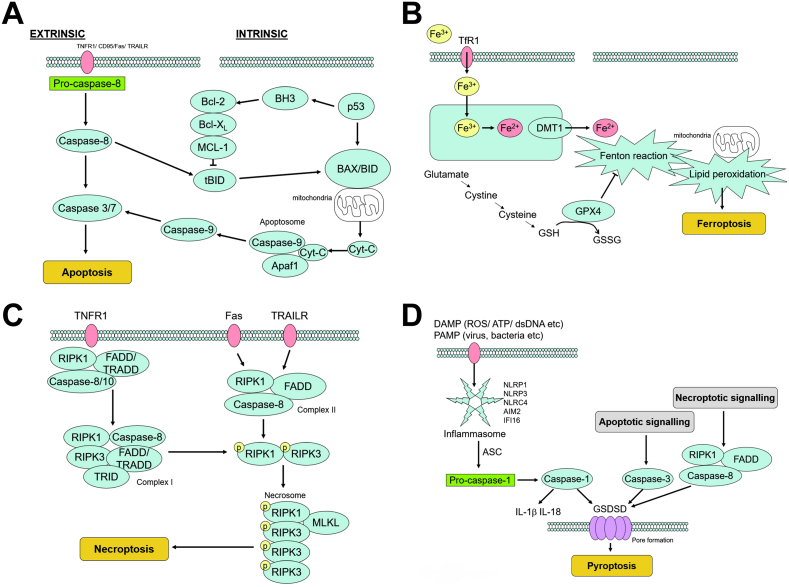
**A: Apoptosis signaling.** Apoptosis can be triggered by either via either extrinsic or intrinsic pathways. The former is typically triggered by the tumor necrosis factor receptor (TNFR), CD95/Fas or the tumor necrosis factor-related apoptosis inducing ligand receptor (TRAILR). The intrinsic pathway utilizes the tumor suppressing protein, p53, that may activate pro-apoptotic Bcl-2 family members, such as Bax and Bid. **B: Ferroptosis signaling.** Ferroptosis in myocardial ischemia-reperfusion is a consequence of ROS generation resulting from the Fenton reaction of ferric iron and mediated through down-regulation of glutathione peroxidase-4 (GPX4) during ischemia and reperfusion. The resulting lipid peroxidation leads to severe mitochondrial damage. **C: Necroptosis signaling.** Extrinsic activation of necroptotic signaling pathway occurs via similar receptors that also result in apoptosis: TNFR, Fas and TRAILR. However, the key difference is the recruitment of receptor-interacting serine/threonine-protein kinase (RIPK) RIPK1 and RIPK3 and facilitated through mixed lineage kinase domain like pseudo kinase (MLKL). **D: Pyroptosis signaling.** Pyroptosis is a response to damage associated molecular patterns (DAMPs) and pathogen associated molecular patterns (PAMPs) that signal to lead to the formation of the inflammasome that consists of sensor proteins and through association with apoptosis-associated spec-like protein containing a caspase activation and recruitment domain (ASC) to form a focus within the cell, and then recruit and activate caspase-1, ultimately leading to cytosolic perforation by gasdermin-D (GSDMD). Interestingly, apoptosis, through caspase-3, and necroptosis, through complex II (RIPK1/FADD/caspase-8), also lead to oligomerization of GSDM.

***Apoptosis*** is characterized by cell shrinkage, chromatin condensation, plasma membrane blebbing without rupture, formation of apoptotic bodies and cytoskeletal disintegration. The apoptotic pathway can be initiated either extrinsically, through activation of cell surface receptors, tumor necrosis factor receptor 1 (TNFR1), CD95/FS-7-associated surface (Fas) antigen receptor and tumor necrosis factor-related apoptosis inducing ligand receptor (TRAILR), through cleavage of pro-caspase-8 and activation of caspase 3 (Fig. 4 A). The intrinsic pathway is initiated by the assembly of the apoptosome – a protein complex consisting of caspase-9, Apaf-1 and mitochondrial released cytochrome-c. This leads to the activation of the distal caspases in the final common pathway of apoptosis: caspase 3 (Fig. 4 A). The apoptotic pathway is itself regulated by the Bcl-2 family of proteins, that alternately lead to activation of apoptosis, through permeabilization of the mitochondrial outer membrane by oligomerization of Bax/Bak [63]. Anti-apoptotic Bcl-2 family members are up-regulated through their phosphorylation by salvage kinases, such as PI3K and Akt [64]. Apoptotic initiator protein p53 is another target for phosphorylation, through the mitogen-activated protein kinase (MAPK)/extracellular signal-regulated kinase (ERK1/2) pathway, which can also function to attenuate downstream caspase activation pivotal to apoptotic signaling; this signaling pathway is complex [65] and depending on the stress that the cell has been subjected to can be either pro- or anti-apoptotic.

Importantly, apoptosis is largely “non-inflammatory” – the apoptotic cell is presented for phagocytosis by macrophages without inducing an inflammatory response, a process that contrasts with the pro-inflammatory phagocytosis of necrotic cells [66]. However, apoptotic signaling may interfere with helpful autophagy and commit a potentially reversibly injured cell to unnecessary death. Given this mixed picture it is useful to note that broad caspase inhibition attenuates I/R injury [67]. Thus targeting caspase may be beneficial in attenuating apoptosis and other cell death pathways, such as pyroptosis, to promote survival. Apoptosis is a “silent” form of cell death without an inflammatory reaction.

***Ferroptosis*** is a relatively novel mechanism of cell death that is distinct from the other modes of cell death discussed here, leading to a unique histological sequence of mitochondrial shrinkage, increased mitochondria membrane density, cristae destruction and outer mitochondrial membrane rupture. These changes however occur in the absence of nuclear morphological change, whereas mitochondrial rupture is likely to lead to apoptotic or pyroptotic signaling activation. In I/R injury, ferroptosis is largely driven by down-regulation of glutathione peroxidase-4 (GPX4) [68], oxidizing glutathione (GSH) and generating ferric (Fe^2+^) iron from the Fenton reaction. This will lead to lipid peroxidation and ferroptotic mitochondrial injury (Fig. 4 B). Ferroptotic ROS generation may, therefore, contribute to myocardial cell death, and interestingly, iron chelators have been shown to improve myocardial survival [69]. Thus, cardiac iron may be a target for novel cardioprotective strategies, attenuating ROS burden of I/R injury [70]. Ferroptosis derives its name from iron-catalyzed reactions but is essentially characterized by a defect of glutathione peroxidase-4 and consequent lipid peroxidation.

***Necroptosis*** has some signaling similarities with apoptosis, but the cellular fate is quite different, leading to cytoplasmic and organelle swelling, formation of the necrosome, plasma membrane rupture and release of cellular constituent histological ends that appear more similar to necrotic cell death. The involvement of death receptor activation (TNFR1, Fas and TAILR) leads to activation of receptor-interacting serine/threonine-protein kinase (RIPK) RIPK1, FAS-associated death domain protein (FADD)/TNF receptor type 1-associated death domain protein (TRADD) and caspases 8/10. This protein complex in turn leads to formation of the necrosome complex to induce necroptosis (Fig. 4C) [71]. Necroptosis is characterized by ROS formation, random degradation of DNA and DAMP release. Class 1A isoforms of PI3K are likely involved in RIPK1/RIPK3 signaling pathway activation [72], but the specific isoforms involved with this signaling are not yet known. In addition to the canonical activation pathway, necroptosis can also be instigated by the recruitment of Ca^2+^/calmodulin-dependent protein kinase II (CaMKII) that itself may be a target for phosphorylation and activation by RIPK3 [73].

Necroptosis is etymologically a cellular response to microbial infection and intended to drive a host immune response, but within the context of the pathophysiology of I/R injury, this may be a less helpful response; the release of DAMPs will extend the wave of cell death within the necrotic core of the ischemic zone. Inhibition of RIP1 signaling by necrostatin-1 has been shown to attenuate I/R injury [74], and phase 2 safety and efficacy trials in chronic inflammation have been undertaken of a small-molecule RIP1/RIP1K inhibitor, GSK2982772 [75] – but thus far, this inhibitor has not tested in the cardiovascular system. Necroptosis shares most features with necrosis but is regulated with involvement of RIP1 and can be specifically inhibited.

***Pyroptosis*** is the prototypical response to DAMP and pathogen associated molecular pattern (PAMP) signaling, characterized histologically by cytoplasmic swelling, formation of pyroptotic bodies, plasma membrane rupture, release of cell contents and without loss of mitochondrial integrity. The pyroptotic inflammasome is a heterodimer of sensor molecules, that cleaves pro-caspase-1 (Fig. 4 D). Caspase-1 leads to the release of inflammatory interleukins IL-1β and IL-18, by polymerizing gasdermin-D (GSDMD) to form a pyroptotic perforating pore in the cytoplasm, leading to cellular lysis [76]. The pyroptotic GSDMD pore can also be induced through alternate pathways, involving capase-3 activity and via RIPK1/FADD/capase-8 that are involved in apoptotic and pyroptotic pathways, respectively. These proteolytic pathways illustrate the interplay of death pathways during I/R injury, and like necroptosis, pyroptosis is a pathogen response pathway to microbial infection that is not ideal in the context of I/R injury. Inhibiting proteases and the formation of the GSDMD pore are the most obvious targets for therapeutic intervention and indeed, GSDMD knockout has been shown to reduce I/R injury in mouse models [77]. Pyroptosis in an inflammatory form of cell death and interacts with inflammatory cells possibly to progressively extend the borders of the infarcting myocardium.

#### 1.3.3 Targets for intervention

Identifying the characteristics of cell death during the hyperacute phase of I/R injury is a vital step in identifying potential targets for therapeutic intervention. To date, much of the research interest has concentrated on the canonical conditioning pathway, via the reperfusion injury salvage kinase (RISK) group of signaling pathways that include PI3K/Akt, MAPK/ERK(1/2) and downstream targets inclusive of mTOR and the mitochondria, specifically the mitochondrial PTP via cyclophilin-D regulation [78]. The survivor activating factor enhancement (SAFE) pathway involving the transcription factor, signal transducer and activator of transcription 3 (STAT3) is an alternate pathway that also targets mitochondrial viability [[79], [80], [81], [82], [83], [84], [85]].

Traditional single occasion RIC administered by 4 times of 5 min intermittent limb occlusion acts through the RISK pathway, which includes the PI3K-Akt-PKC-ERK signalling cascade, as documented in a variety of species, including rodents and pigs [86,87] and even with transferability across species [82]. Studies in isolated mice hearts have demonstrated that RIC also initiates autophagy [88]. The initial activation of the autophagosome during the first window of protection alleviates the damage of I/R injury and supports cell function by clearing damaged protein aggregates, by removing damaged ROS-producing mitochondria and through the recycling of macromolecules for use in cell repair [88]. A study of exosomes from rats has shown that this response may appear as long as 48 h after the RIC stimulus [89]. Preservation of post-ischemic cardiac function, measured by post-ischemic LV end-diastolic and developed pressure, is similar by acute, delayed and repeated RIC in experimental studies of rats and mice [88,90,91].

PI3K is an obvious upstream target for cardioprotection, involved in cellular survival pathways. However, its role is complex: class I PI3K is intrinsically linked to activation of pyroptotic mechanisms, whereas class III PI3K is associated with autophagy. The key to unlocking the potential of PI3K as a target for cardioprotection is the recognition of not only the different classes of PI3K, but also the different isoforms within each class. For example, there are a number of PI3K class I p110 subunit isoforms (α, β, ꝩ), which have the potential for differential regulation of cell-survival pathways. The α isoform has been linked to acute cardioprotection [92], and Gong et al. have reported on the discovery of UCL-TRO-1938, a small molecule activator of the PI3Kα isoform that provides significant cardioprotection against I/R injury [93]. Similarly, ROS are intrinsic to hyperacute injury, and managing ROS generation from enzymatic processes, such as from NOX, mitochondrial complex I or from dysregulated ferric iron Fenton reactions will also represent important therapeutic targets for cardioprotection. Beyond these initiator processes, targeting and modifying programmed cell death provide additional targets for protection. Pyroptotic cell death and cells destined for necroptosis are likely to be key in these instances – thus, preventing the formation of the GSDMD pore or inhibiting RIPK1/RIPK3 activation are likely key for the attenuation of acute inflammatory myocardial injury.

However, it may be too simplistic to target just one pathway and expect this to translate to significantly reduced myocardial injury in man given human heterogeneity, comorbidities and concomitant drug use [57,94]. However, a multi-modal approach to inhibit cellular rupture pathways and promote cellular survival in both the hyperacute phase and through secondary acute genomic transcription of pro-survival proteins would seem to be the optimal approach to future clinical translation.

#### 1.3.4 Confounders of myocardial ischemia/reperfusion injury and cardioprotection

Confounders of myocardial I/R injury and cardioprotection have just recently been reviewed in great detail [95], therefore only briefly in here. Aging [96] and the classical risk factors of hypertension [97], hyperlipidemia [98] and diabetes [99] not only predispose to the development and progression of coronary atherosclerosis, but also sensitize the myocardium to I/R injury and interfere with cardioprotective signal transduction. Female sex before menopause appears to protect from I/R injury in most rodent models [100], but in pigs there is no difference in I/R injury per se and in protection by ischemic preconditioning (IPC) [101]. Patients with coronary artery disease and acute myocardial I/R typically have a number of medications which can interfere with cardioprotection; some of them protect per se and may limit the potential for further protection, e.g. nitroglycerine, morphine, P2Y_12_ inhibitors [102], some of them interfere with cardioprotective signaling, e.g. sulfonylureas [103]. Whether or not such interference with cardioprotection is really of clinical importance, is still largely uncertain [104].

### 1.4 The coronary circulation as culprit and target of myocardial ischemia/reperfusion injury

**Fig. 5:**
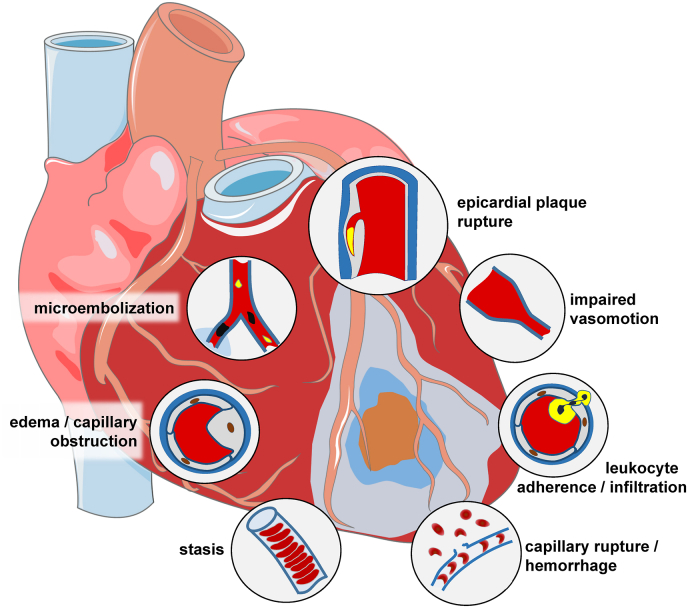
Schematic presentation of mechanisms contributing to coronary microvascular injury with ischemia/reperfusion. From [113].

Atherosclerosis of the coronary circulation is the cause of myocardial ischemia which is best defined as a critical reduction of coronary blood flow such that the physiological electrical and contractile processes and ultimately cellular integrity and viability are no longer maintained [105]. The coronary circulation is both a culprit and a target of myocardial I/R. In acute myocardial infarction, the rupture of an epicardial coronary atherosclerotic plaque initiates immediate intravascular thrombosis and occlusion of the affected coronary artery at the atherosclerotic lesion site [106]. More recently, particularly with the increasing use of statins, plaque erosion is becoming more frequent [107], and plaque erosion more often causes coronary microembolization [108] and non-STEMI [109]. With underlying atherosclerosis and acute plaque rupture or erosion, the epicardial coronary circulation is clearly a culprit of myocardial ischemia. More recently, awareness has increased that also coronary microvascular disease in the absence of significant epicardial coronary obstruction can initiate acute myocardial infarction, and this occurs more frequently in women than in men [110,111]. Such coronary microvascular disease is characterized by endothelial dysfunction, enhanced vasoconstrictor responsiveness and reduced coronary dilator reserve on an adenosine challenge, and it is diagnosed by a combination of imaging procedures [111,112]. The role of the coronary circulation as a victim of myocardial I/R has long been neglected but is now clear and receiving increasing attention [113,114]. I/R injury to the coronary circulation affects predominantly the microcirculation, both endothelial and vascular smooth muscle cells, and has multiple mechanisms. Preclinical experimental studies have elaborated on increased vascular permeability and edema formation [115], impaired vasomotion notably as a consequence of endothelial dysfunction [116] and release of soluble vasoconstrictor substances from epicardial coronary lesions [117], adherence of platelets and leukocytes to the endothelium [118], formation of platelet aggregates [119] and erythrocyte stasis [120], microembolization of particulate atherosclerotic and thrombotic material from the epicardial coronary lesion [108], and ultimately capillary rupture [121] with hemorrhage into the interstitium [122]. The manifestations of coronary microvascular injury can be reversible (edema, impaired vasomotion, intravascular cell aggregates) or irreversible (particulate embolization, capillary rupture, hemorrhage) (Fig. 5). Clinically, the impairment of the coronary circulation during reperfusion following myocardial ischemia is known for a long time from interventional procedures for stable coronary artery disease and from thrombolytic and interventional reperfusion for AMI. The diagnosis and quantification of coronary vascular impairment in these settings has traditionally been made from angiographic indices (TIMI flow grade, TIMI frame count, myocardial blush grade) or intravascular Doppler flow velocity and flow velocity reserve [123,124]. The reference standard for the assessment of coronary microvascular impairment is now CMR, which – along with infarct size and contractile function – can quantify magnitude and spatial extent of edema, no-reflow, and hemorrhage [125]. It is now evident, that in fact coronary microvascular obstruction – beyond infarct size – is a major determinant of the prognosis for patients with reperfused AMI [126], as is intramyocardial hemorrhage [127]. These recent data make coronary microvascular obstruction a worthwhile target for clinical attempts of cardioprotection.

The role of ROS in the coronary circulation displays the typical concentration-dependent ambivalence - signaling function at lower concentrations and injury at excessive concentrations [128,129]. ROS, notably hydrogen peroxide (H_2_O_2_), are released from human coronary arterioles and mediate flow-dependent dilation [130], thus exhibiting a physiological function; NOX and endothelial mitochondria contribute to this ROS release [131,132]. However, in myocardial I/R ROS contribute to coronary microvascular injury [133]. The specific contribution of ROS to coronary vascular vs. cardiomyocyte injury from I/R cannot be distinguished at this point [113]. Specific targeting of mitochondrial ROS formation by use of transgenic mice with endothelial manganese-dependent superoxide dismutase improved angiogenesis, decreased scar size and preserved left ventricular (LV) function after permanent coronary occlusion [134].

In preclinical experiments, all of the above mechanisms of coronary microvascular injury could be successfully targeted, and the respective injury could be attenuated, typically by mechanical interventions of ischemic pre- and post-conditioning and RIC, but also by drugs relating to the signal transduction of ischemic conditioning procedures [2,135]. In fact, IPC reduced edema, endothelial dysfunction, leukocyte adherence and coronary microvascular obstruction, and it improved coronary vasomotion. Ischemic postconditioning attenuated edema and endothelial dysfunction, and it reduced no-reflow. RIC reduced no-reflow along with infarct size [101]. Some drugs also attenuated no-reflow along with infarct size, and angiopoietin-like peptide 4 appears to have a specific protective action on the coronary microcirculation [136]. Ischemic preconditioning is not feasible in patients with AMI, but a number of studies using CMR in patients with reperfused AMI have demonstrated that ischemic postconditioning [[137], [138], [139]] and RIC [140,141] attenuated edema, no-reflow and intramyocardial hemorrhage, sometimes along with infarct size reduction, but sometimes not [114]. In a post-hoc analysis of METOCARD, metoprolol also reduced coronary microvascular obstruction, as assessed by CMR, in 106 vs. 114 control patients with reperfused STEMI [142]. It has been suggested that the consideration of the coronary microcirculation as a site of I/R injury and consequently as a target of cardioprotection will improve the translation from preclinical studies to clinical practice [2,36,143]. The remodeling process following reperfusion of myocardial infarction and eventually leading to heart failure [24] also involves the coronary circulation, notably through angiogenesis [144], and coronary blood flow again impacts on heart failure and *vice versa* [145].

### 1.5 Percutaneous coronary intervention in chronic coronary syndrome

For many patients with obstructive coronary artery disease, percutaneous coronary intervention (PCI) remains the major revascularization strategy of choice, with an estimated 5 million procedures performed worldwide each year [146]. A substantial number of chronic coronary syndrome (CCS) patients undergoing elective PCI experience procedural-related myocardial injury and infarction, the occurrences of which are associated with an increased risk of future major adverse cardiovascular events (MACE) such as death, re-infarction, and revascularization [147,148]. As such, PCI-related myocardial injury and infarction are important targets for cardioprotection especially for those undergoing complex higher-risk PCI procedures.

PCI-related myocardial infarction or type 4a MI has been defined by the Fourth Universal Definition of MI (UDMI) [149] as a post-PCI elevation of cTn >5 × 99th percentile upper reference limit (URL) within 48 h of the PCI procedure in patients with normal baseline (pre-PCI) values and associated ECG/imaging/angiographic evidence of new myocardial ischemia. However, post-PCI elevations in cTn in the absence of new evidence of myocardial ischemia are indicative of peri-procedural myocardial injury (PMI). The Fourth UDMI has defined PMI as any post-PCI elevation of cTn >1 × 99th URL in patients with normal baseline values [149]. The prognostic relevance of such definition has been questioned, although recent data suggest that a post-PCI cTn cut-off elevation of >5 × 99th percentile URL is the optimum threshold for independently predicting all-cause mortality at one year in terms of sensitivity and specificity [147].

The cause of PMI and type 4a MI is multifactorial and may be due to side-branch occlusions, distal coronary embolization of intracoronary thrombus and atheromatous material [108], coronary vasospasm due to neuro-hormonal activation, and other PCI-related factors such as pre-dilation, partially occlusive devices (such as catheter extension devices, retrograde chronic total occlusion (CTO) procedures, atherectomy devices), which can result in prolonged total vessel occlusion times [147].

Several cardioprotective strategies have been evaluated for their ability to reduce PMI and type 4a MI in CCS patients when administered prior to PCI although the endpoints for cardioprotection which have been used have varied from study to study (Table 1, Table 4) [[150], [151], [152], [153]]. One of the most promising interventions are high-dose statins (e.g., atorvastatin 80 mg or rosuvastatin 40 mg) which when administered prior to PCI have been reported to reduce the risk of PMI, type 4a MI, and MACE in CCS patients [[154], [155], [156], [157], [158]], although not all studies have been positive [159,160]. The mechanisms of cardioprotection as identified from pre-clinical animal studies appear to be pleiotropic, involving the upregulation of cytoprotective pathways including RISK, decreased inflammation, inhibition of platelet aggregation, improvement of endothelial function, and plaque stabilization [161,162].

**Table 1:** Therapeutic strategies to prevent periprocedural myocardial injury and type 4a myocardial infarction in chronic coronary syndrome patients.

Agent	Timing of administration	Potential mechanism of action	Findings
High-dose Statins	Pre-PCI	Pleiotropic effect on inflammation [161,162] Production of endothelial progenitor cells [162]	Several RCTs have reported ↓ incidence of periprocedural myocardial injury and type 4a MI [[154], [155], [156], [157]]. A meta-analysis of 14 trials reported ↓ incidence of periprocedural myocardial injury, type 4a MI and MACE (death, re-infarction and revascularization [158]). However, neutral effects in some studies [159,160].
Cangrelor	At the time of PCI (intravenous)	Intravenous P2Y12 platelet inhibitor	Large multicentre RCT (CHAMPION PHOENIX) of 11,145 CCS patients reported ↓ incidence of periprocedural myocardial injury and type 4a MI when compared to clopidogrel [150]
Remote ischemic conditioning	Pre-PCI (3–4 5-min cycles of limb ischemia/reperfusion)	Reduces acute myocardial ischemia-reperfusion injury	Mixed results with some positive studies reporting ↓ incidence of periprocedural myocardial injury and type 4a MI [[163], [164], [165]], with other studies showing no reduction in PMI [166] One follow-up RCT of 225 CCS patients showing ↓ incidence of MACE (not powered for clinical outcomes) [167] Meta-analysis of 11 studies showed no overall benefit with RIC [168]
Vitamin C	Pre-PCI (IV infusion)	Anti-oxidant effects	RCT of 532 CCS patients showing ↓incidence of periprocedural myocardial injury [151] RCT of 56 CCS patients showed ↑microcirculatory reperfusion [152].
Enalaprilat	At the time of PCI (intracoronary)	Endothelium-dependent epicardial coronary vasodilation mediated by endogenous bradykinin activity	RCT of 40 CCS patients showing ↓ incidence of periprocedural myocardial injury [153]
Colchicine	At the time of PCI	Anti-inflammatory effects	RCT [172] of 400 CCS did not show any impact on PCI-related myocardial injury. RCT of 5545 CCS patients showed 31% reduction in cardiovascular death, spontaneous (nonprocedural) myocardial infarction, ischemic stroke, or ischemia-driven coronary revascularization. However, the incidence of death from noncardiovascular causes was higher in the colchicine group than in the placebo group (HR 1.51) [171].

Other cardioprotective interventions which have been investigated in CCS patients undergoing PCI include RIC although the results have been mixed [[163], [164], [165], [166], [167]], and a recent meta-analysis of 11 studies found no overall beneficial effects in terms of reduced PMI as assessed by elevated circulating cTn levels [168]. A small clinical study has investigated the combined effects of high-dose atorvastatin and RIC and reported additive cardioprotective effects with reduced PMI when compared to high-dose statin alone [169]. A recent study has reported beneficial effects with RIC reducing PMI in patients undergoing PCI with drug-coated balloons, an intervention associated with prolonged angioplasty inflation times and reduced risk of restenosis [170].

More recently, low-dose treatment with the anti-inflammatory agent, colchicine, has been reported to reduce mainly ischemia-driven clinical events in CCS patients [171]. However, pre-treatment of CCS patients with high-dose colchicine prior to PCI failed to reduce the incidence of PMI, type 4a MI, or Society of CardiovascuIar Angiography and Intervention-defined PMI, when compared to placebo [172]. Whether or not post-PCI treatment with low-dose colchicine can reduce major adverse cardiovascular events in CCS patients experiencing type 4a MI post-PCI is not known. It must be noted that the incidence of death from non-cardiovascular causes was higher in the low-dose colchicine group than in the placebo group (hazard ratio 1.51) [171].

Since the occurrences of PCI-related myocardial injury and type 4a MI in CCS patients are associated with worse clinical outcomes, this form of injury is an obvious therapeutic target for cardioprotection and improving patient outcomes especially in patients undergoing complex PCI procedures. However, the multi-factorial nature of PCI-related myocardial injury and type 4a MI may, in part, explain why it has been challenging to demonstrate effective cardioprotection against this form of injury.

### 1.6 Cardioprotection in coronary artery bypass grafting

With the advent of the cardiopulmonary bypass (CPB) [173], surgeons were able to perform more complex operations such as those on the aorta and coronary artery bypass grafting (CABG). Surgeons generally need to arrest the heart in order to perform the operation. Cardiac arrest is performed with the delivery of a hyperkalemic solution (cardioplegia) to reduce its metabolic rate after commencing CPB in order to maintain perfusion to the body while the heart and lungs are not moving. Despite its routine use and safety in most cases, cardioplegia and CPB are not without potential adverse consequences. Cardioplegia protects the heart from ischemic injury, reperfusion injury and inflammation, but myocardial injury or even infarction occur in some cases despite the surgeon's best efforts to prevent it. CPB and cardioplegia do not lessen surgical trauma and do not prevent graft failure, valve repair failure, intraoperative aortic dissection or other catastrophic events that sometimes occur and carry a poor outcome.

CPB and cardioplegia and the subsequent myocardial reperfusion have been associated with intraoperative and postoperative myocardial and microvascular dysfunction [[174], [175], [176]]. Hypothermia was introduced to further decrease myocardial metabolism during cardiac arrest and further improve outcomes [173]. Numerous studies in animal models and subsequent clinical trials have validated the principles of depolarizing hypothermic potassium cardioplegia [[177], [178], [179], [180]].

Since its invention [181], there have been many refinements of CPB to accompany cardioplegic arrest, including coatings of the bypass circuit to reduce cellular activation and thrombosis, filters, and more efficient oxygenators. Today, hypothermic, hyperkalemic cardioplegia under conditions of CPB is a routine, integral tool for cardiac surgeons performing a variety of cardiovascular operations. Despite its effective use in most cases, myocardial stunning [182] and injury [183,184] occur to some extent in most cardiovascular operations. The administration of hyperkalemic cardioplegia solution decreases myocardial oxidative stress [180]. Increased oxidative stress by myocardial ischemia, cardioplegia and CPB may affect levels of nitric oxide and other oxidative moieties and affect tissue integrity and enzyme function [[184], [185], [186]]. The restoration of blood flow to ischemic tissue after termination of cardioplegia and CPB further stimulates pathways involving oxidative processes, leading to the further generation of ROS [[184], [185], [186]].

Despite variability in composition, delivery and temperature, most cardioplegic solutions in use today involve some level of potassium chloride as the main inducer of cardiac arrest, along with ions such as magnesium, low-dose calcium and a pH buffer such as bicarbonate. After an initial delivery to arrest the heart, the cardioplegia solution is then given every 15–20 min due to “wash out” of the solution. Blood is often used as a buffer in a 4:1 blood to cardioplegia ratio, but the optimal cardioplegia remains debated [187,188]. The standard “modern” cardioplegic solution consists of a hyperkalemic (15–25 mmol/l), hypothermic (4–8 °C) crystalloid solution mixed in a 1:4 ratio with blood from the patient. A low concentration of magnesium is often added to limit calcium sequestration in the myocytes. The hemoglobin probably does not provide much oxygen delivery to the heart during arrest, but may act as a buffer to acidosis. Despite the theoretical advantage of adding blood or another buffer to the cardioplegic solution, few studies have definitively demonstrated a significant clinical benefit of using one solution over another [187,188].

#### 1.6.1 Adjuvants to cardioplegia for myocardial protection

There have been many attempts to improve myocardial protection during cardiac surgery including drugs to reduce oxidative stress, neutrophil infiltration and sequestration, and complement activation, but none of these adjuvant drugs have been found to provide a clinically significant improvement in outcomes after surgery. IPC initially provided much enthusiasm for the development of novel methods to diminish the effects of myocardial ischemia during coronary occlusion and cardiac surgery. Intermittent aortic cross-clamping has been used clinically, especially prior to the refinement of methods of cardioplegic arrest. Improved cardioprotection has been demonstrated in animal models with lessened release of biomarkers [189]. Similar protection was seen in patients undergoing coronary artery bypass grafting with aortic cross-clamping [190,191] or a modified preconditioning protocol by intermittent hypoxic perfusion of the unloaded heart [192]. RIC has been examined in several clinical trials and the results have been mixed [[193], [194], [195], [196], [197], [198], [199], [200]]. A study by Thielmann et al. [201] found improved biomarker evidence of cardioprotection and early outcomes including a slight mortality benefit and less repeat revascularization with RIC. However, two prospective, phase III trials [ERICCA [193] and RIPHeart [198]] of RIC in patients undergoing cardiovascular surgery found no difference in cTn release or clinical outcome after 1 year [199]. In both studies, patients were anesthetized with propofol, which may diminish the effects of RIC (Table 2, Table 4) [[202], [203], [204]]. In a meta-analysis of 15 clinical trials, inhalation agents and beta-blockers were also found to attenuate the effects of RIC [205]. Interestingly, in this meta-analysis, valve surgery patients seemed to derive more benefit than CABG patients from RIC.

**Table 2:** Selected clinical trials of ischemic conditioning in cardiac surgery.

Study	n	Population/Treatment	Findings
Hausenloy et al. [193]	57	CABG patients, randomized to RIPC vs. control	Less troponin release with RIPC
Xie JJ et al. [194]	73	Patients with valve surgery, randomized to RIPC vs control	Less troponin release and better cardiac function with RIPC
Kottenberg et al. [203]	27 230	CABG, diabetic patients treated with sulphonylureas vs. non-diabetics, RIPC	Non-diabetic patients had less troponin release with RIPC, diabetic patients treated with sulphonylureas had no change in troponin with RIPC.
Thielmann et al. [201]	329	CABG patients, randomized to RIPC vs. sham	Less troponin release, less MACCE
Zhou et al. [205]		Meta-analysis of 15 randomized trials	Benefit of RIPC shown but β-blockers and volatile anesthetics attenuated benefits of RIPC in adult cardiac surgery
Zhang et al. [196]		Meta-analysis of 9 randomized trials, RIPC vs. control in CABG patients	No benefit of RIPC on troponin release or clinical outcomes
Hong et al. [195]	1280	Cardiac surgery patients, randomized to RIPC with RIPostC vs. control	No effect on clinical outcome
Zangrillo et al. [204]		Meta-analysis of 55 randomized trials in cardiac surgery of RIPC with and without volatile anesthetics	Both RIPC and volatile anesthetics improved outcome, combination was best.
Kurapeev et al. [192]	90	CABG patients, preconditioning induced by ischemia and reperfusion on CPB prior to CP, perfusion alone or control. All patients had blood CP.	Less troponin release and better clinical outcome with preconditioning
Hausenloy et al. [197]	1612	Multi-center randomized trial, randomized to RIPC vs. sham (anesthesia not controlled)	No difference in combined endpoint of MI, stroke, CV death, revascularization, kidney injury at 12 months, no early differences in troponin release, acute kidney injury or quality of life
Meybohm et al. [198]	1403	Cardiac surgery patients, RIPC or sham (propofol used)	No benefit of RIPC demonstrated in composite outcome of death, MI, stroke, renal failure early or at 90 days follow up
Kleinbongard et al. [199]	329	CABG patients, randomized to RIPC vs. control	Improved short term recovery and survival, persistent benefit for up to 9 years
Moscarelli et al. [200]	124	2 center randomized trial with heart surgery patients (CABG and AVR) RIPC vs. sham control	No difference in troponin release or release of inflammatory cytokines, serum creatinine or lactate, myocardial ATP

AVR, aortic valve replacement; CABG, coronary artery bypass grafting; CP, cardioplegia; MACCE, major adverse cardiac and cerebrovascular events; MI, myocardial infarction; RIPC, remote ischemic preconditioning; RIPostC= Remote ischemic postconditioning.

Because several clinical trials demonstrated little if any clinically significant benefit of ischemic conditioning, it is currently rarely used as an adjuvant to hypothermic cardioplegia. In fact, the act of providing intermittent coronary or peripheral vascular occlusion is time consuming, awkward at times, and disrupts the flow of the time-sensitive operation. An improvement in myocardial contractile function with ischemic conditioning is generally not as clearly demonstrated in larger animal models such as the pig or human [206]. This may in part explain the improvement in cTn release without a benefit in cardiac function or other clinical outcomes in clinical trials.

As opposed to when a patient arrives to the emergency room with an AMI requiring emergent treatment, the condition of a patient undergoing elective or even urgent cardiac surgery can often be optimized prior to the operation. This may include controlling blood glucose and hypertension, treating infections, inflammatory disorders and renal insufficiency and optimizing preoperative cardiac function. These interventions can have a marked effect on improving the outcomes of cardiac operations. Patients with coronary artery disease have pre-existing endothelial dysfunction which contributes to postoperative microvascular dysfunction. Microvascular changes in reactivity and permeability are well documented after cardiac surgery in the coronary circulation and in the circulation of brain, skeletal muscle and many other vascular beds [184]. In clinical studies, poorly controlled hypertension [176] or diabetes [[207], [208], [209]] are associated with a marked increase in post-operative changes in both vascular reactivity and permeability. Preoperative hypercholesterolemia has been shown to increase microvascular endothelial injury, oxidative stress and infarct size in a porcine model of acute myocardial I/R [210]. In order to best understand the effects of cardioprotective procedures in the human heart, it may be prudent to examine human myocardial tissue [176,[207], [208], [209],^211^,^212^]. Cardiovascular operations are the ideal source for this human atrial and ventricular myocardium and other tissues such as skeletal muscle.

### 1.7 Myocardial infarction and cardioprotection

The implementation of PPCI resulted in a marked decrease of morbidity and mortality in STEMI patients [[213], [214], [215]]. Improvements of clinical outcome are closely linked to a reduction of infarct size [26]. However, during the last years mortality rates have plateaued [18]. Therefore, additional approaches are needed to further improve clinical outcome. Over the past 3 to 4 decades, many cardioprotective strategies against myocardial I/R injury have been proposed in AMI. In general, these can be divided into several categories based on the protective modality, time of application, cellular and also the intracellular target.

#### 1.7.1 Ischemic conditioning

**Fig. 6:**
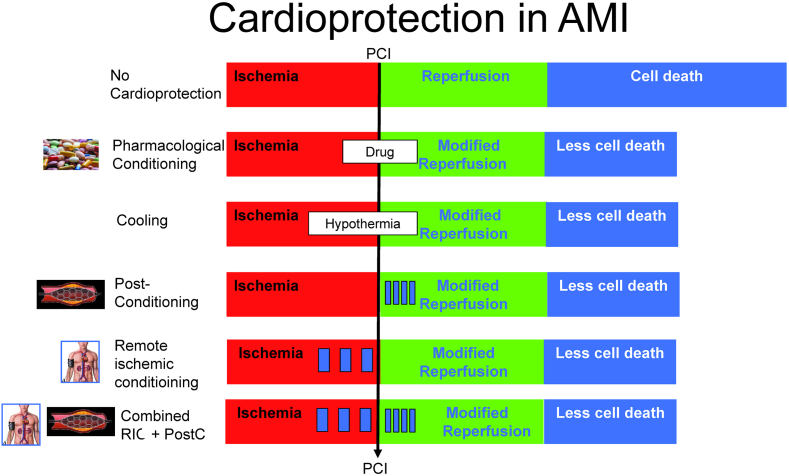
Schematic diagram of ischemic conditioning interventions and their impact on infarct size.

The best studied cardioprotective modalities that have garnered significant attention are RIC and local ischemic postconditioning (PostC). These techniques aim to protect the heart against I/R injury through distinct mechanisms and offer potential benefits in terms of reduced infarct size with possible subsequent improvement in cardiac function and clinical outcome such as reduced mortality or reduction in heart failure hospitalization. The most studied intervention is RIC. In the majority of trials RIC has been induced by 4 alternating cycles of 5 min inflation to 200 mmHg followed by 5 min deflation of an upper arm blood pressure cuff (Fig. 6) [41]. Numerous trials showed a reduction of enzymatic infarct size with RIC in STEMI patients. All these studies used remote ischemic per- or postconditioning by means of limb ischemia using a pneumatic cuff [140,141,[216], [217], [218]]. Other studies, including the large CONDI-2/ERIC-PPCI trial, failed to show a significant reduction of enzymatic infarct size with RIC [41,219,220]. White et al. reported a reduction of CMR imaging-derived infarct size with RIC, whereas other studies did not show a significant effect on CMR-derived infarct size [141,220]. Botker et al. demonstrated improved myocardial salvage index, measured by single photon emission computed tomography (SPECT), with RIC compared to standard PCI [219]. PostC involves the application of brief cycles of myocardial ischemia and reperfusion immediately after the restoration of blood flow following sustained ischemia. Usually, repeated balloon inflation is used [217,221]. Within 1 min of re-opening of the infarct-related artery the angioplasty balloon is positioned at the site of the index lesion and re-inflated 4 times at 4–6 atm. Each inflation usually lasts 1 min followed by 1 min of reperfusion (Fig. 6). To ensure re-occlusion of the coronary artery a small dose of contrast agent is usually injected during balloon inflation. In case of incomplete occlusion an increase of inflation pressure is recommended. Experimental studies have provided compelling evidence for the cardioprotective effects of PostC, with reduced infarct size, improved myocardial function, and preservation of endothelial function observed in animal models. However, clinical studies investigating PostC in STEMI have yielded mixed results, with some trials reporting positive effects on infarct size while others have shown no significant benefit [57,202]. The LIPSIA CONDITIONING trial showed higher CMR-derived myocardial salvage index with a combined strategy of RIC and PostC which is currently the only clinical trial testing this combined approach [222]. Another study showed reduced CMR-determined myocardial edema following remote ischemic postconditioning, whereas no significant differences in other CMR parameters were detected [137].

Data supporting a clinical benefit with RIC in STEMI patients are scarce. Post-hoc analyses from the LIPSIA-CONDITIONING trial suggested improved long-term prognosis with combined RIC and PostC compared to conventional treatment [223]. This finding was driven by a significant reduction of new congestive heart failure at long-term follow-up. The long-term outcome analysis of the CONDI 1 trial suggested a lower rate of the combined endpoint as well as all-cause mortality in the RIC group [224].

The implementation of STEMI networks during the last two decades resulted in a marked decrease of door-to-balloon time in STEMI patients [225,226]. Consequently, STEMI patients present in improved hemodynamic condition on hospital admission, which is associated with improved outcome. In the recent European CONDI-2/ERIC-PPCI trial approximately 96% of patients presented with no hemodynamic complications, i.e. Killip class I, resulting in low cardiac mortality rates of about 3% at 1 year [41]. Not surprisingly, in such an ideal setting of patient care a potential further reduction of myocardial damage by ischemic conditioning interventions will not translate in additional prognostic benefit [28].

However, RIC and PostC might be of clinical benefit in higher risk STEMI patients with hemodynamic complications, i.e. Killip class ≥2, where mortality rates are much higher, offering more space for potential prognostic benefit [227]. Indeed, the cardioprotective effects of RIC increase with ischemia time in STEMI patients [228]. In the LIPSIA-CONDITIONING trial, a trend towards improved myocardial salvage index with RIC plus PostC compared to conventional treatment was observed in patients with Killip class ≥2 [222]. Furthermore, a recent observational study showed improved clinical outcome with RIC at 90 days in STEMI presenting with cardiogenic shock or cardiac arrest [229].

Accordingly, ischemic conditioning might be of particular benefit in the setting of less well developed STEMI networks, where transport delays are longer and reperfusion therapy is often not optimal [28]. Therefore, the effect of ischemic conditioning should finally be tested in a randomized controlled trial in a high-risk STEMI population [230]. Currently, the ongoing RIP-HIGH randomized multi-center trial in Germany and Austria investigates the effect of combined RIC plus PostC compared to standard treatment on clinical outcome and myocardial damage in a high-risk STEMI population. The trial uses an adaptive design, and currently >100 patients have been randomized. Trial results are expected by the end of 2024 (clinicaltrials.gov: NCT 04844931).

#### 1.7.2 Pharmacological protection

A variety of pharmacological approaches to cardioprotection have been tried, many of them attempting to recruit signalling steps of local or remote ischemic pre-, per-, and postconditioning. The results are for the most part disappointing. They can all be summarized in that most experimental studies which had shown a benefit could also be transferred into human studies with a reduction in infarct size. However, subsequent large-scale clinical randomized trials all failed to show a benefit in clinical endpoints.

Selected studies on cardioprotection by pharmacological agents can be found in Table 3, Table 4 and have been summarized in multiple reviews [36,44,57], including cyclosporine A [[231], [232], [233]] and other mitochondria-targeting agents [234,235], nitrite [236,237], inhaled nitric oxide [238], the ROS scavenger acetylcysteine without [239] or with nitroglycerine [240], anti-inflammatory interventions [241,242], inhibition of the protein kinase C delta isoform with delcasertib [243], and beta blockade with metoprolol [244,245]. Cyclosporine reduced infarct size, as measured by CPK release and CMR in a small trial [231], but in a subsequent larger trial neither reduced CPK release nor improved clinical outcome after 1 year [232]. Other trials targeting the mitochondria also did not report reduced infarct size or better clinical outcome [246]. Reflecting lack of robust preclinical data [247], initial promising results with metoprolol in the METOCARD trial [244] were not confirmed in the much larger EARLY-BAMI trial [245]. Other agents which have reduced infarct size but did not result in improved clinical outcome include adenosine [248], atrial natriuretic peptide [249], and exenatide [250].

**Table 3:** Selected clinical trials of pharmacological cardioprotection in patients with reperfused acute myocardial infarction.

Study	n	Patient criteria	Treatment protocol	Main outcome
**Cyclosporin-A**				
Piot et al. [231]	58	All STEMI	Intravenous bolus of CsA administered 10 min prior to PPCI	Reduced MI size assessed by AUC CK. No difference in troponin I. Subset of 37 patient reduce MI size on MRI at day 5 post-PPCI
Cung et al. [232] CIRCUS	970	Anterior STEMI Pre-PPCI TIMI 0/1	Intravenous bolus of CsA administered prior to PPCI	No difference in primary outcome worsening in-pt heart failure, HHF, or adverse LV remodeling at 1 year
Ottani et al. [233] CYCLE	410	All STEMI	Intravenous bolus of CsA administered prior to PPCI	No difference in primary endpoint of ≥70% ST-segment resolution 60 min After TIMI flow grade 3, or MI size (day 4 hs-cTnT) or LV remodeling at 6 months
**MTP-131**				
Gibson et al. [234] EMBRACE-STEMI	118	Anterior STEMI Pre-PPCI TIMI 0/1	Intravenous 60 min infusion of MTP-131 started prior to PPCI	No difference in primary endpoint of MI size (72 h AUC CK). No difference in MI size or LV remodeling on MRI at 4 ad 30 days
**TRO40303**				
Atar et al. [235] MITOCARE	163	All STEMI within 6 h chest pain Pre-PPCI TIMI 0/1	Intravenous bolus of TRO40303 administered prior to PPCI	No difference in primary endpoint of MI size (72 h AUC CK or hs-cTnI), significant increase in major adverse events
**Nitrite**				
Siddiqi et al. [236] NIAMI	229	All STEMI TIMI 0/1	Intravenous bolus of nitrite administered prior to PPCI	No difference in primary endpoint of MI size on MRI at day 6–8. No difference in LV remodeling or MI size by (72 h AUC CK or cTnI)
Jones et al. [237]	198	All STEMI	Intracoronary bolus of nitrite administered prior to PPCI	No difference in primary endpoint of MI size (72 h AUC CK or hs-cTnI)
***N*-acetylcysteine**				
Thiele et al. [239] LIPSIA-*N*-ACC	251	All STEMI	2 × 1200 mg/day *N*-ACC for 48 h	No difference in myocardial salvage index measured by CMR at day 3–4 (43.5; IQR 25.4 to 71.9 vs. 51.5; IQR 29.5 to 75.3; p = 0.36)
***N*-acetylcysteine + nitroglycerin**				
Pasupathy et al. [240] NACIAM	75	All STEMI	Intravenous infusion of NAC for 48 h initiated prior to PPCI. On background of IV GTN infusion.	Reduction (by 33%) in primary endpoint of MI size by CMR at day 2–3 post-PPCI
**Inhaled nitric oxide**				
Janssens et al. [238] NOMI	250	All STEMI	Inhaled oxygen with NO started 10 min prior to PPCI and continued for 4 h.	No difference in primary endpoint of MI size by MRI at day 2–3
Kleveland et al. [241]	117	NSTEMI	Intravenous 60 min infusion started prior to PPCI	Reduced hsCRP levels. Reduced median AUC for hs-cTnT by 30%
Broch et al. [242] ASSAIL-MI	199	All STEMI	Intravenous 60 min infusion started during PPCI	Increased myocardial salvage by 5.6% on CMR (2–7 days) and less MVO but no difference in MI size
**PKC-δ inhibition (delcasertib)**				
Lincoff et al. [191] PROTECION-AMI	1010 166	Anterior STEMI Inferior STEMI	Intravenous infusion prior to PPCI for 2.5 h	No difference in CK-MB, troponin, and ST segment resolution
**β-blocker (metoprolol)**				
Ibanez et al. [244] METOCARD-CNIC	220	Anterior STEMI Killip ≤2	Intravenous infusion before PPCI	Infarct size by CMR reduced from 32 to 25.6 g
Roolvink et al. [245] EARLY-BAMI	342	STEMI Killip ≤2	Intravenous infusion before PPCI	Infarct size by CMR not reduced

AUC, area under curve; CMR: cardiac magnetic resonance; CK-MB, creatine kinase MB isoenzyme; GTN, glyceryl trinitrate; hs-cTnT/I, high-sensitive cardiac troponin T/I; HHF, hospitalization for heart failure; LAD, left anterior descending coronary artery; MI, myocardial infarction; MVO, microvascular obstruction; NAC, *N*-acetylcysteine; NSTEMI, non-ST-segment elevation myocardial infarction; PPCI, primary percutaneous coronary intervention; STEMI, ST-segment elevation myocardial infarction.

**Table 4:** Clinical trial acronyms.

AMI HOT I, II [[263], [264]]	Acute myocardial infarction with hyperoxemic therapy
ASSAIL-MI [242]	Assessing the effect of anti-IL6 treatment in myocardial infarction
CHAMPION PHOENIX [150]	A clinical trial comparing cangrelor to clopidogrel standard of care therapy in subjects who require PCI
CHILL-MI [257]	Efficacy of endovascular catheter cooling combined with cold saline for treatment of acute myocardial infarction
CIRCUS [232]	Cyclosporine A in reperfused acute myocardial infarction
CONDI 1 [219]	Effect of remote ischemic conditioning during evolving ST-elevation myocardial infarction
CONDI 2 ERIC/PPCI [41]	Effect of remote ischemic conditioning on clinical outcome in patients with acute myocardial infarction
COOL AMI [259]	Trial to assess cooling as an adjunctive therapy to percutaneous intervention in patients with acute myocardial infarction
CYCLE [233]	Cyclosporine A in reperfused acute myocardial infarction
EARLY-BAMI [245]	Early intravenous beta blocker in patients with ST-segment elevation myocardial infarction before PPCI
EMBRACE-STEMI [234]	Trial to evaluate safety, tolerability and efficacy of intravenous bendavia on reperfusion injury in patients treated with standard therapy including PCI and stenting for STEMI
ERICCA [193]	Effect of remote ischemic preconditioning on clinical outcomes in patients undergoing CABG surgery
GIPS-IV (NCT 02899364)	Groningen intervention study for the preservation of cardiac function with sodium thiosulfate after STEMI
LIPSIA-CONDITIONING [222]	Leipzig cardioprotection by combined intrahospital remote ischemic preconditioning and postconditioning in ST-elevation myocardial infarction
LIPSIA-*N*-ACC [239]	Leipzig immediate PCI acute myocardial infarction *N*-ACC trial
METOCARD-CNIC [244]	Effect of metoprolol on infarct size in ST-segment elevation myocardial infarction undergoing PPCI
MITOCARE [235]	Mitochondrial care in acute myocardial infarction
NACIAM [240]	*N*-acetylcysteine in acute myocardial infarction
NIAMI [236]	Intravenous sodium nitrite in acute ST-elevation myocardial infarction
NOMI [238]	Nitric oxide inhalation in ST-elevation myocardial infarction
PROTECTION-AMI [231]	Inhibition of delta protein kinase C for the reduction of infarct size in acute myocardial infarction
RAPID-MI-ICE [258]	Rapid intravascular cooling in myocardial infarction as adjunct to PCI
RESILIENCE (NCT05223413)	Remote ischemic conditioning in lymphoma patients receiving anthracyclines
RIC-AFRICA [42]	Remote ischemic conditioning in African patients with myocardial infarction
RIC-STEMI [37]	Remote ischemic conditioning in ST-segment elevation myocardial infarction as adjuvant to primary angioplasty
RIP-HEART [198]	Remote ischemic preconditioning in heart surgery
RIP-HIGH (NCT04844931)	Remote ischemic preconditioning in high risk myocardial infarction
SHOCK-COOL [261]	Mild hypothermia in cardiogenic shock complicating myocardial infarction
STEMI-DTU (NCT03947619)	Door to unloading with Impella system in acute myocardial infarction
STOP-CA (NCT 02943590)	Statins to prevent the cardiotoxicity from anthracyclines

#### 1.7.3 Physical intervention

Physical interventions are numerous and even aspiration thrombectomy targeting thrombus and possible peripheral embolization and reperfusion injury may be summarized under physical interventions [108,251]. However, in general, physical interventions aiming at reducing the classical reperfusion injury such as LV unloading, hypothermia or hyperoxemia are considered treatment strategies. LV unloading reduced infarct size in a pig model of myocardial infarction and in preliminary studies in STEMI patients, even when the duration of ischemia was prolonged by the unloading procedure [252], but the mechanism [253] and clinical benefit from LV unloading are still unclear; the STEMI-DTU (NCT03947619) will provide further answers. Experimental studies in different animal species have consistently shown that mild hypothermia, induced prior or during reperfusion in acute MI, reduces infarct size [254]. Cooling before reperfusion therefore appears to be a promising adjunct to PPCI in STEMI patients. Several randomized clinical trials have shown mixed results, although intravascular cooling appeared to be safe and well tolerated [[255], [256], [257]]. A combined analysis of RAPID MI-ICE and CHILL MI revealed a significant reduction in infarct size in a subgroup of early presenters with anterior STEMI who were cooled below 35 °C prior to reperfusion [258].

A similar favorable signal was also observed in the COOL AMI pilot randomized trial, which enrolled 50 patients with anterior STEMI and resulted in a numerical reduction in infarct size (23.8%–16.7% of the left ventricle; p = 0.31) [259]. In the COOL AMI EU Pivotal Trial, powered to demonstrate a clinically meaningful reduction in infarct size using rapid intravascular systemic hypothermia prior to PPCI compared to standard treatment with PPCI alone, hypothermia resulted in a longer ischemic delay and did not reduce infarct size. There was also a concern with associated increased adverse events [260]. In the randomized SHOCK-COOL trial, moderate hypothermia in cardiogenic shock patients with AMI did not result in improved hemodynamics in comparison to control. However, hypothermia was associated with higher lactate levels, possibly even indicating harm in this setting of cardiogenic shock [261]. Randomized clinical trials powered to show differences in clinical outcome are not available for hypothermia in AMI. Data on hyperoxemic reperfusion are controversial [262]. Final infarct size as measured by SPECT 2 weeks after PPCI in STEMI patients was not reduced in the AMIHOT I trial [263]. In contrast, infarct size was reduced in the AMIHOT II trial [264]. However, clinical outcome was not affected by hyperoxemic reperfusion.

In summary, the quest for novel strategies to protect the myocardium against I/R injury in acute MI continues to drive scientific research. Rigorous large-scale randomized controlled trials are needed to evaluate the safety, efficacy, and optimal protocols for these cardioprotective strategies. Investigations into the combination of these approaches with existing therapies and identification of specific patient populations that may benefit the most are also warranted.

### 1.8 Heart failure and cardioprotection

Although interventional approaches for coronary reperfusion and improvement in procedures and logistics have reduced the incidence of post-infarction heart failure [265], heart failure following AMI continues to develop with cumulative incidence rates between 12 and 22% after 2.9 and 3 years, respectively [266,267]. This adverse condition remains associated with a two to three times increased risk of subsequent death or hospital admissions [267]. Most cardioprotective interventions to improve survival and prevent subsequent chronic heart failure have focused on reducing the immediate I/R injury during AMI by mechanical ischemic conditioning strategies or pharmacological interventions. A more recent approach has aimed to explore the potential of applying repeated daily conditioning strategies as an adjunct to the standard recommended medication that modifies post-infarction remodeling in order to reverse the physical limitation in patients, when heart failure has developed [268,269]. The approach takes advantage of the unique feature of a biphasic pattern of cardioprotection by mechanical conditioning strategies such as RIC. The first window of protection initiates instantaneously and lasts for ∼2–3 h. Following a period without protection, the second window of protection reappears between 12 and 24 h after the original stimulus and lasts for up to 72–96 h [[270], [271], [272]]. Because RIC is easily and non-invasively applied, the intervention can be administered repeatedly for extended periods, such as once daily for weeks. Experimental studies have demonstrated that RIC repeated daily for 28 days after AMI protected against adverse left ventricular remodeling and increased survival in a rat model even though infarct size was not reduced further compared with the single-occasion treatment [90]. The improvement in LV chamber size, LV function and hemodynamic changes after AMI was largest in the group that received repeated RIC every day for 28 days compared to a control group and two groups receiving RIC either during ischemia or every third day following AMI [90]. A similar beneficial effect on remodeling was obtained even when RIC treatment in rats was commenced as long as 4 weeks after AMI, thus documenting favorable influence of repeated daily RIC in a more chronic phase of AMI [91]. Hence, the benefit appears to go beyond the initial improvement by reduction in infarct size, suggesting that a distinct mechanism of cardioprotection may act directly on remodeling.

The mechanisms underlying RIC involve an initial reduction in oxidative stress and blunting of the inflammatory response that downgrades cytokine signaling and reduces levels of neutrophil and macrophage infiltration in the myocardium in an in-vivo rat model of myocardial infarction [90]. Repeated RIC adds an attenuation of the expression of genes associated with fibrosis and hypertrophy [90]. In the rat study initiating repeated RIC 4 weeks after AMI, an association between reduced systemic oxidative stress and attenuation of AMI-induced left ventricular interstitial fibrosis in the boundary region of the infarct [91] provided direct mechanistic evidence for modulation of remodeling after infarct healing by delayed repeated RIC.

Beyond the interfering patterns of kinase and autophagy activity by acute single occasion RIC, repeated RIC in rats adds modulation of separate phagosomes that involve downregulation of mTOR and subsequent upregulation of pro-autophagy proteins [88], supporting that separate mechanisms mediate distinct benefits on LV remodeling as compared to a single occasion RIC stimulus given at the time of the infarction. Inter-organ signaling by single occasion RIC was initially shown to involve neuronal as well as humoral mediators [87]. More recent studies have shown that extracellular vesicles and exosomes [273], containing proteins, lipids, mRNAs and microRNAs, mediate cardioprotective inter-organ communication in rodents [274,275], and that this mediation is transferable between species [276]. In repeated RIC, the effects on the heart also involve mediation by exosomes as microRNA-29a (miR-29a), a key regulator for reduction of tissue fibrosis, is highly expressed in the exosomes and in the infarct border zone in the RIC group, when RIC is initiated in rats 4 weeks after AMI [91]. Moreover, insulin-like growth factor 1 receptor (IGF-1R) is highly expressed in the exosomes and remote non-infarcted myocardium of the RIC group [91]. A role for exosome mediation is supported by studies in mice, demonstrating that RIC induces release of skeletal muscle exosomes, in which proteome analyses related ∼20 dominating proteins to signaling pathways responsible for the synthesis, contraction, and relaxation of cardiac muscle [277].

So, experimental findings point towards a beneficial effect of repeated daily RIC on adverse remodeling during the phase of infarct healing and beyond. It remains to be determined how autophagy signaling specifically contributes to the remodeling process and to which extent remodeling can be modulated in a chronic condition of completed infarct healing. An additional limitation of the experimental studies is that studies so far have been conducted only in healthy animals that were not treated with the post-infarction medication usually administered to humans with heart failure and known to interfere with RIC efficacy [95].

In humans, repeated RIC initiated on day 3 following AMI in patients treated with PPCI and given once daily for 4 weeks did not affect LV ejection fraction, infarct size or left ventricular end-diastolic and systolic volumes after 4 month [278]. Reduction of infarct size achieved by single occasion RIC immediately prior to PPCI is a major determinant for improvement in LV ejection fraction and remodeling in particular among patients with extensive area at risk [279]. Even though a critical period, when inflammation and oxidative stress contribute to adverse LV remodeling, may be within the first 3 days post-infarction, a subsequent more comprehensive CMR study of the same patients demonstrated that repeated RIC, commenced 3 days after infarction, initiated improvement in cardiac remodeling after 4 months [280]. A similar beneficial modulation of cardiac remodeling, measured as global longitudinal shortening, by repeated RIC once daily for 4 weeks has been demonstrated among the most severely compromised patients with stable chronic ischemic congestive heart failure as defined by NT-proBNP plasma levels above the geometric mean of 372 ng/l [268]. Also, blood pressure and circulating NT-proBNP were lowered by RIC. Overall, LV ejection fraction, peak cardiopulmonary exercise capacity and disease-related quality of life did not significantly change in this outcome-assessor blinded study [268]. RIC applied twice daily for 7 days on both arms increased coronary flow reserve modestly in patients with chronic congestive heart failure due to ischemic or dilated cardiomyopathy [281]. An impressive effect was demonstrated in a single-blinded randomized trial without sham control that revealed improved LV ejection fraction (from 39.2% to 43.4%, assessed by echocardiography), increased exercise capacity (assessed by 6-min walk test), reduced NYHA class, and lower levels of plasma BNP with repeated RIC given as 4 × 5 min cycles of upper arm cuff inflation and deflation twice daily for 6 weeks in patients with chronic ischemic heart failure randomized to RIC or standard therapy [269]. Overall, the findings may indicate a potential for modifying further adverse cardiac remodeling in established heart failure.

The mechanisms underlying the beneficial effects of repeated RIC on NT-proBNP and remodeling are not clear but may relate to less myocardial wall stress, caused by reduction in afterload consistent with lowering of systemic blood pressure by repeated RIC. This may be mediated by release of known vasodilator mediators of RIC such as adenosine and nitric oxide [268] and a mild anti-inflammatory effect with modest reductions in C-reactive protein and calprotectin [282] and reduced neutrophil adhesion [283]. The upregulation of cardioprotective microRNAs, nitric oxide production, and oxidative stress reduction that facilitate reverse LV remodeling in the post-infarction healing period [284] may be analogous mechanisms in chronic heart failure patients. Also, the beneficial effects of repeated RIC in heart failure patients have been associated with correction of cardiac autonomic dysfunction by increased parasympathetic and reduced sympathetic activity, as assessed by heart rate variability [269]. Other specific signaling pathways have not yet been investigated specifically in heart failure patients. Exosome mediation of cardioprotection may be involved as translational studies indicate that labelled exosomes in plasma from healthy volunteers following single occasion RIC accumulate more intensely in the infarct area than in sham hearts, when transferred to a Langendorff rat model [276].

Beyond the effect on the heart, repeated RIC seems to enhance skeletal muscle strength in heart failure patients, so that muscle waste, known as an inherent component of heart failure, may be prevented and disease-related quality of life improved [268]. This is consistent with findings in trained athletes and non-athletes, in whom RIC improves physical performance [285]. Blood flow restricted exercise (BFRE) may represent a more potent performance-enhancing corollary to RIC in heart failure patients [286]. The concept may be of aid in optimizing physical rehabilitation in populations that are not be able to perform exercise practice at intensity levels required to promote optimal outcome through mechanisms mobilizing endogenous protective like by RIC [287]. Such mechanisms involve secretion of myokines, growth factors [288] and muscle stem cells [289,290] mediated by micro-RNAs delivered to remote locations via exosome transport [291].

### 1.9 Cardio-oncology and cardioprotection

Significant advances in cancer therapy have greatly reduced mortality, with non-malignant comorbid conditions becoming important determinants of their quality of life and overall survival. Among this heterogeneous group of comorbid conditions, cardiovascular diseases are major contributors to overall morbidity and mortality in cancer survivors [292,293]. Cardiovascular diseases and cancer share common risk factors in both the aged and the pediatric populations and are further linked through toxicities in the cardiovascular system effects of contemporary cancer treatment.

The first anticancer agents widely used were anthracyclines. Anthracyclines are a non-specific agent with highly effective anti-tumoral capacity. Still today, anthracyclines remain first line treatment (alone or in combination with other therapies) for many cancer types, including lymphomas, leukemias, sarcomas and breast cancer. The principal limitation to use of high-dose anthracyclines is their established cardiotoxic effect [294]. One in every 3 patients receiving anthracyclines develops some form of cardiotoxicity [295]. Fortunately, this cardiotoxicity is mild and transient most of the times, but in more than 5% of patients who receive anthracyclines, irreversible cardiac dysfunction with associated chronic heart failure occurs [295]. Given the high number of patients receiving anthracyclines in Europe every year, the prevalence of chronic heart failure in cancer survivors directly related to anthracycline cardiotoxicity is estimated in 1 million EU citizens. For cancer survivors, the trade-off between cancer and chronic heart failure is of massive psychological burden. For healthcare systems, the growing incidence of chronic heart failure has devastating economic consequences. Treatment for cancer has massively evolved in recent decades, and now there are new highly specific (targeted) therapies for specific types of cancers. These include human epidermal growth factor receptor 2 (HER2)-targeted interventions, tyrosine kinase inhibitors, immune checkpoint inhibitors, proteasome inhibitors, androgen deprivation therapy, and chimeric antigen receptor T cells. These therapies are used most often as part of combos, which include anthracyclines. The use of targeted therapies is not free from cardiovascular adverse events, and the development of cardiac dysfunction and heart failure are common side effects of several targeted therapies. Targeted therapies have also been associated with increased incidence of cardiovascular side effects different from cardiac dysfunction, such as myocarditis, accelerated atherosclerosis and arterial hypertension [294,296,297]. From all the above, it is obvious that there is a need to identify cardioprotective strategies that, when used in combination with anti-cancer therapies, can prevent the development of these unwanted cardiovascular side effects [297]. One major limitation for the identification of cardioprotective therapies in this setting is the incomplete understanding of the mechanisms leading to cardiovascular toxicities of these anticancer treatments. Despite considerable inter-individual vulnerability, anthracycline cardiotoxicity is strongly associated with the cumulative dose received throughout life. These figures are the basis for the recommendation for a maximum lifetime cumulative dose of 400 mg/m^2^ for all patients receiving anthracyclines [298]. The maximum recommended dose also depends on whether or not mediastinal radiation therapy is given, which increases the risk of cardiotoxicity. Patients with previous cardiovascular comorbidities (hypertension, diabetes, alcohol consumption, etc.) are at higher risk for developing cardiotoxicity [293]. There are important similarities between the mechanisms leading to cardiac injury from I/R and from anthracycline cardiotoxicity. These include mitochondrial damage with massive ROS production [299], and microvascular injury [300]. Early detection of anthracycline cardiotoxicity can be made by using multiparametric CMR imaging, since this can detect intra-cardiomyocyte edema as a result of mitochondrial vacuolization as part of quality control during injury [301].

Given its high clinical relevance, therapies that can prevent the development of anthracycline cardiotoxicity have been sought for several years at the experimental and clinical levels [293]. Several interventions have been tested with inconsistent results [294]. Most of the clinical trials testing cardioprotective strategies for this condition have included a very limited number of patients, precluding final conclusions. Beta-blockers [[302], [303], [304]], renin-angiotensin-aldosterone-system inhibitors [[305], [306], [307]], and statins [[308], [309], [310], [311]] are among the most frequently tested therapies. The STOP-CA trial compared atorvastatin 40 mg against placebo in 300 patients with lymphoma receiving moderately high dose anthracycline regime. The incidence of cardiotoxicity events (defined according to a pre-specified decline in LV systolic function) was significantly reduced in patients allocated to statin preventive treatment. In contrast, in another (smaller) recent trial including patients with several cancer types, atorvastatin 40 mg did not show cardioprotection as evaluated by CMR imaging [311,312].

**Fig. 7:**
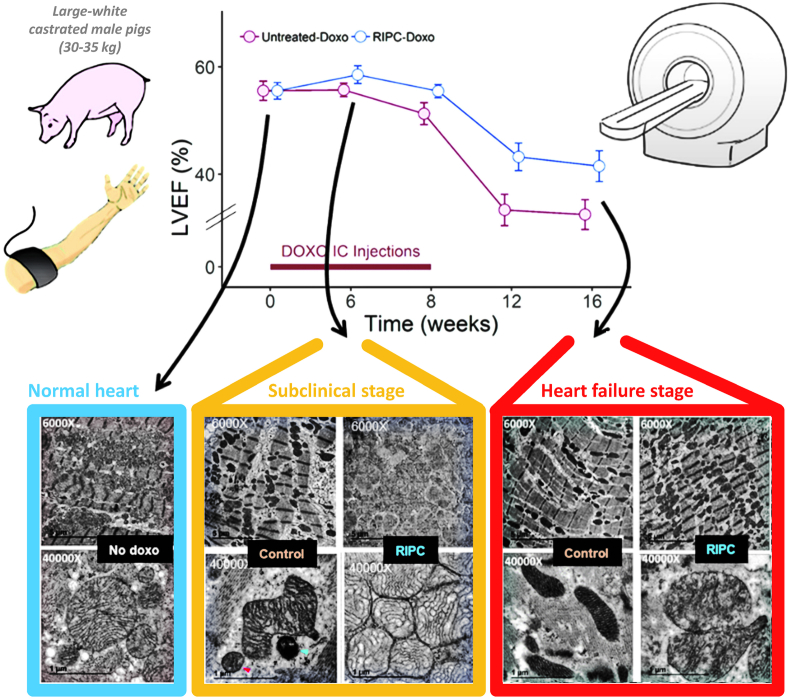
Effect of remote ischemic preconditioning (RIPC) on left ventricular ejection fraction (LVEF) and mitochondrial structure in a pig model of anthracycline cardiotoxicity. Pigs received five biweekly intracoronary doxorubicin injections with RIPC of control applied before each injection. Serial cardiac magnetic resonance imaging studies were performed during 4 months follow-up. Anthracycline-induced systolic dysfunction was significantly attenuated in pigs receiving RIPC. At the ultrastructural level, mitochondria were fragmented in pigs receiving doxorubicin since subclinical stages (i.e. a time where cardiac function was still normal). At late stages (i.e. when severe cardiac dysfunction was present), mitochondria were severely damaged (electrodense and ultra-fragmented). Mitochondria from pigs undergoing RIPC before each anthracycline administration were much more preserved at every timepoint. Figure adapted from [299].

Given the similarities between the mechanisms of damage of I/R injury and anthracycline cardiotoxicity, it is intuitive to argue that RIC is an ideal intervention. The fact that the exposure to the injury agent (anthracyclines) is a programmed intervention (administration of chemotherapy in a controlled hospital environment), it is in theory the ideal clinical setting to test RIC. By using a highly translatable pig model of anthracycline cardiotoxicity within long-term serial examinations by state-of-the-art CMR imaging, Galan-Arriola et al. recently demonstrated the strong cardioprotective effects of RIC when applied before each anthracycline administration [299]. Pigs randomized to RIC before each anthracycline injection displayed a significant preservation of cardiac systolic function. This was associated with structural and functional preservation of mitochondria, less ROS production, and attenuation of dysregulated autophagy. (Fig. 7). The effect of RIC to prevent anthracycline cardiotoxicity has been tested in 2 very recent very small pilot trials, one in an adult population [313], and another in a pediatric one [314]. Both trials were neutral for the primary endpoint (release of cTn), however cardiotoxicity (as defined by clinical practice guidelines [293] was not observed in patients in any of the trials. Of note, in the small trial in adult patients RIC increased cancer and total mortality over placebo [313], raising the concern that RIC might induce a systemic survival signal which benefits not only the heart, but also the cancer [315]. These considerations highlight that future trials should enroll patients at risk for anthracycline cardiotoxicity and get away from the all-comers design [316]. The ongoing ‘REmote iSchemic condItioning in Lymphoma PatIents REceiving ANthraCyclinEs’ (RESILIENCE) trial (NCT05223413) is testing the benefits of RIC in a population with one or more high-risk features for cardiotoxicity. In this European Commission-funded trial, 608 patients will be randomized to weekly RIC (4 × 5 min cycles) or sham during the period on chemotherapy (approx. 4 months). Primary outcome measure will be based on serial CMR imaging.

## 2 Sources and actions of reactive oxygen species

### 2.1 Reactive oxygen species in myocardial ischemia/reperfusion - the early days

A role for ROS in biology originated more than a century ago when investigators asked why obligate anaerobic bacteria were killed by the presence of oxygen. In 1907 it was found that oxygen toxicity in bacteria was correlated with the absence of catalase which was known to scavenge H_2_O_2_, a highly toxic oxygen derivative. Catalase was present in most aerobic bacteria tested and absent in all obligate anaerobes [317]. This led to the hypothesis that oxygen caused formation of H_2_O_2_, a ROS, in bacteria that was lethal if they lacked the scavenger catalase. But where did the H_2_O_2_ come from? In 1968 Joe McCord and Irwin Fridovich [318] were studying the reduction of cytochrome *c* by milk xanthine oxidase. They found this reaction could be blocked by carbonic anhydrase even though it does not bind to cytochrome *c*. They subsequently found that the xanthine oxidase was generating the free radical superoxide which was reducing the cytochrome *c* and that there was a contaminant in their carbonic anhydrase that was dismutating superoxide to H_2_O_2_ thus halting the reduction reaction. In a follow-up study they purified the contaminant and named it superoxide dismutase (SOD). They found that it, like catalase, is widely distributed among all mammalian species tested, including man [319].

Three ROS received particular attention in the 1980s: superoxide radical (O_2_*^-^), H_2_O_2_, and hydroxyl radical (*OH). Radicals are molecules with unpaired electrons in one of the shells which make them highly reactive. H_2_O_2_, while not a radical is still very reactive. All three of these ROS have the potential to disrupt vital molecules like enzymes and nucleic acids through redox reactions. H_2_O_2_ produced by SOD is reduced to water by catalase. H_2_O_2_ in the presence of free Fe^2+^ or Cu^2+^ can be catalyzed into the highly reactive hydroxyl radical. Thus a combination of SOD plus catalase should eliminate all three ROS.

Following the discovery of SOD and the apparent importance of the body's ROS scavenging systems, ROS quickly moved to the forefront of scientific research. Popular culture also picked up on the ROS hypothesis. Foods were advertised as being chock-full of anti-oxidants. Even James Bond, in a conversation with miss Moneypenny, likened members of his arch enemy SPECTOR to “free radicals” (Never Say Never Again, 1983). Among the proposed ROS targets was the ischemic heart. It seemed a likely setting for such a ROS disaster.

In 1973 David Hearse and colleagues [320] looked at the effect of hypoxia-reoxygenation in isolated rat hearts and noted that reoxygenation was accompanied by an abrupt release of cytosolic enzymes into the venous effluent, a marker of cell death by membrane failure. This was termed the “oxygen paradox”. The reason why oxygen, which is obviously needed for the hypoxic heart to recover, also appeared to causes a reoxygenation injury was unknown, but ROS were mentioned among other possible explanations. The first paper claiming that SOD could reduce infarct size appeared in 1984 using canine hearts experiencing I/R [321]. SOD and catalase are both large proteins that would not easily enter cells. However, it was known that the reperfused myocardium is soon flooded with neutrophils. It was proposed that neutrophils inappropriately directed a ROS attack on injured but still viable cardiomyocytes and that SOD in the extracellular space prevented that attack [322].

**Fig. 8:**
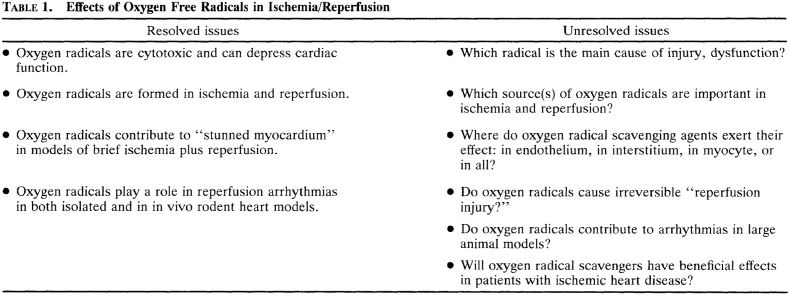
The view in 1989 on pathomechanisms of I/R injury and the open questions on the role of ROS in it. From Ref. [327] with permission.

What followed was a cascade of studies in which infarct size was measured in animal hearts subjected to an ischemic insult followed by reperfusion. A search of PubMed today for SOD and infarct size brings up more than 1000 hits. SOD with or without catalase was tested in these models, but the results were very mixed. Some labs reported robust protection while others saw little or none. Except for a very early canine study [323], Downey's lab reported mostly negative SOD results [324,325]. In 1989 two seminal papers were published on the role of ROS in the ischemic heart, one by Keith Reimer [326] and the other by Robert Kloner [327]. Central concepts and open questions at that time are shown in Fig. 8.

Both reviews took a critical look at the many published reports and came to the conclusion that the more sophisticated the animal models became the less protective the SOD was. They also seriously challenged the neutrophil hypothesis. Today most investigators think neutrophils act to debride the infarcted myocardium to promote healing rather that contribute to its infarction, since infarct size is amazingly similar in blood-free Langendorff hearts and in situ hearts [328].

Looking back, many of the positive reports on SOD's protection were probably the result of artifacts in the infarct size measurement itself. Unfortunately, there are numerous potential pitfalls in the methods used to measure infarct size in animal hearts that can lead to erroneous findings. These include poor histological techniques for identifying infarcted tissue and failure to accurately measure the infarct's volume. Failure to accurately account for the factors that affect infarct size including risk zone size, collateral flow and temperature of the heart during ischemia can all lead to uncontrolled variability in the measurement of infarct size. Most of the early studies used dogs which were very expensive so group sizes were usually small. Miura et al. [329] reported a study of 54 untreated dogs subjected to 4, 24, 48 or 96 h of permanent coronary occlusion. Fig. 2 reveals that infarct size ranged from 4% to 95% of the risk zone and was inversely related to the level of collateral flow to the ischemic zone which ranged from as high as 88% of the pre-occlusion flow to as low as 2%. Thus without measuring collateral flow, as was the case in many of the early canine studies, it would be virtually impossible to detect any effect of an intervention on infarct size because of the extreme variability in collateralization among canine hearts. Note that the terminal infarct size was already apparent after 4 h of ischemia since further prolongation of the ischemic time did not increase the infarct size.

Accordingly, in those early canine studies significance often hinged on the presence of one or two key “responders” or on the exclusion of one or two key “outliers”. Additionally, failure to maintain the temperature of the heart during ischemia can introduce large variations of infarct size in isolated, small animal hearts. Van Winkle's group found the infarct size following I/R increases by 7% per degree C during ischemia [330]. Immersing the isolated hearts in 38ᵒC Krebs solution during ischemia greatly reduced variability in infarct size. Today the infarct size model can be quite powerful and robust, but only if all of the factors that affect infarct size are controlled and sufficient numbers of hearts are studied.

Much research was directed at the source of ROS in the reperfused heart. Neil Granger found that xanthine oxidase was responsible for superoxide-induced injury in the reperfused intestine [331], and Downey's lab found similar results in canine hearts. However, unlike canine hearts, human hearts lack xanthine oxidase as do rabbit hearts where xanthine oxidase inhibitors have no effect on infarction [332]. Finally, ROS are not always injurious. IPC was the first intervention found to unambiguously protect the ischemic heart. Its protection involves a complex signal transduction cascade that includes one step that uses a ROS signal. Blocking it eliminates preconditioning's protection [333].

Although SOD failed to salvage the ischemic myocardium, ROS still are thought to be involved in myocardial infarction as in the case of ferroptosis where iron toxicity causes formation of hydroxides and hydroxyl radicals through the reaction of Fe^2+^ and hydrogen peroxide [334]. The toxic ROS are also active in micro-compartments such as the mitochondria [52] that would be unreachable by an extracellular scavenger like SOD or catalase.

### 2.2 Mitochondrial ROS and cardioprotection

As discussed in the section above, ROS play a crucial role in cardiac injury induced by I/R. The question is then where ROS are generated and which are the most relevant targets of oxidative stress in the ischemic heart. Even limiting the discussion just to cardiomyocytes, the question of sources and targets keeps generating a steady flow of debate and controversies, yet is far from being conclusively solved.

Regarding intracellular sites, a consensus exists that mitochondria are responsible for the largest amount of ROS generated especially during post-ischemic reperfusion [335]. Therefore, ROS accumulation adds to other detrimental processes, such as a decrease in ATP synthesis and an increase in [Ca^2+^], by which mitochondria made dysfunctional by the lack of oxygen change from vital organelles into the main executioners of I/R injury. The involvement of mitochondria in I/R injury was introduced more than 50 years ago [336] by associating the loss of cardiomyocyte viability with calcium accumulation within the mitochondrial matrix. Soon thereafter, mitochondrial function was shown to be required for cell death upon reperfusion, since both inhibition of respiratory chain and uncoupling of oxidative phosphorylation decreased enzyme release [337,338]. These seminal observations created a tight link between mitochondrial alterations and necrosis that was extended to apoptosis during the nineties [339]. Furthermore, two elements required for contraction and viability, such as Ca^2+^ and oxygen, were established as main determinants of the loss of function and viability in hearts with I/R [340]. This concept was further elaborated by demonstrating that both Ca^2+^ and ROS accumulation promote the opening of the mitochondrial PTP in a synergistic manner [341]. On this basis, it is hardly surprising that protective strategies were developed to prevent I/R-induced increases in Ca^2+^ and/or ROS, or inhibit PTP opening. However, although in controlled experimental protocols of severe and prolonged ischemia this rationale might prove valid, the failure of attempts to translate that approach into clinical settings [246] is likely to depend on the notion that mitochondrial Ca^2+^ and ROS overload, as well as PTP opening, are involved also in self-defense mechanisms, such as those underlying conditioning-induced cardioprotection [333,342]. The elucidation of mechanistic issues, some of which are discussed below, should allow the improvement of therapeutic strategies.

#### 2.2.1 Pathways of mitochondrial ROS formation and removal

**Fig. 9:**
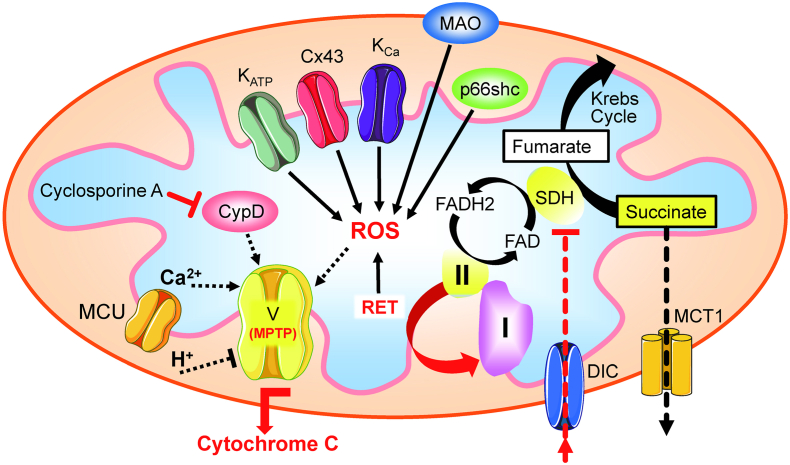
Schematic diagram of ROS-generating systems in mitochondria. Big calcium-activated potassium channel (K_Ca_); connexin 43 (Cx43); cyclophilin D (Cyp D); dicarboxylate carrier (DIC); mitochondrial calcium (Ca^2+^) uniporter (MCU); mitochondrial ATP-dependent potassium channel (K_ATP_); mitochondrial permeability transition pore (MPTP); monoamino oxidase (MAO); p66shc, p66 Src homologous and collagen; reverse electron transport (RET); sarcolemmal monocarboxylate transporter 1 (MCT1); succinate dehydrogenase (SDH). Modified from [379].

The fact that mitochondria are accepeted as the prevailing cellular site for ROS formation does not tell us which mitochondrial sources are relevant in I/R, which one of them is involved in injury or protection, and which targets are more relevant. The answer to those relevant questions is complicated by the involvement of several mitochondrial processes which generate ROS as a role alternative to their vital functions [335]. This is especially the case with the electron transport chain subunits or flavin-containing dehyrogenases. Genetic or pharmacological interventions on those enzymes would affect not only ROS formation, but also bioenergetics and cell viability. Nevertheless, the fact that mitochondria generate ROS in vivo is proven unambiguously by enzymes that generate ROS as their natural products and are not involved directly in bioenergetics. In this respect, the study of monoamine oxidases (MAO) and p66Shc provided a clear demonstration of the detrimental role of ROS generated within mitochondria in I/R, as well as in various cardiac pathologies [343,344] (Fig. 9). Indeed, MAO and p66Shc inhibition or downregulation elicit a high degree of protection against cardiomyocyte and vascular abnormalities [344,345]. Notably, the protective effects were not increased by combining MAO and p66Shc inhibition [346]. A major advantage with MAO inhibition is that it is obtained with clinically available compounds, a feature that is not shared by any other mitochondrial reaction generating ROS.

Besides H_2_O_2_, the oxidative deamination catalyzed by MAO generates ammonia and aldehydes that are likely to synergize in causing mitochondrial and cellular alterations. Of note, the decrease in I/R injury elicited by stimulation of aldehyde dehydrogenase 2 [347], another mitochondrial enzyme, demonstrates the high toxicity of aldehyde moieties, especially those generated from aromatic amines. Indeed, aldehydes that are generated also by means of lipid peroxidation might play a deleterious role similar to or larger than that caused directly by ROS.

Pathways involved in ROS removal lend a further support to the deleterious role of oxidative stress generated in mitochondria. Indeed, SOD down-regulation exacerbated I/R injury [348], whereas cardioprotection was obtained by expressing catalase in mitochondria [349]. Furthermore, mitochondrial membrane potential is related directly to antioxidant defenses by means of uncoupling proteins (UCPs) and nicotinamide nucleotide transhydrogenase (NNT). Overexpression of UCP2, the most abundant cardiac isoform, resulted in cardioprotection, although the underlying mechanism remains controversial [350]. On the other hand, NNT-catalyzed conversion of NADH(H^+^) into NADPH(H^+^) is necessary to maintain peroxidase activities [351].

#### 2.2.2 Mitochondrial ROS and cytosolic processes, back and forth

It is worth pointing out that mitochondrial ROS formation can be triggered by cytosolic events generated in response to physiological or pathological stimuli, such as NOX stimulation or an elevation in intracellular [Ca^2+^]. NOX-induced ROS formation has been suggested to represent an initial trigger for a more substantial ROS generation in mitochondria [352]. Regarding Ca^2+^, although linking mechanisms are still matter of debate [353], an increase in cytosolic and/or mitochondrial [Ca^2+^], such as that associated with an increased beating rate, is paralleled by ROS accumulation [354]. The link could result from Ca^2+^-dependent stimulation of various enzymes. For instance, arachidonic acid generated by Ca^2+^-activated phospholipase A_2_ is a powerful agonist of PTP opening also associated with mitochondrial ROS formation [355]. More recently, as a possible mechanism underlying arrhythmogenic cardiomyopathy, Ca^2+^-activated calpain has been shown to cause mitochondrial ROS formation leading to oxiation of cleaved apoptosis initiating factor that upon its translocation into nuclei triggers cell death [356].

Since mitochondrial ROS formation is mostly the consequence of an initial alteration, such as an elevation in intracellular [Ca^2+^], uncertainities might remain on whether injury is caused by ROS or by a triggering event. The question whether a primary increase in mitochondrial ROS can affect cardiomyocyte function and viability was addressed by using the mitochondria-targeted compound MitoParaquat (MitoPQ) [357]. Acting as a redox cycler at the level of complex I, MitoPQ generates superoxide directly into mitochondria. Submicromolar concentrations of MitoPQ caused mitochondrial dysfunction due to PTP opening and loss of cell viability. At concentrations <0.1 mM viability was slightly affected, but intracellular Ca^2+^ transients were profoundly altered, suggesting that oxidative stress produced in mitochondria can disrupt Ca^2+^ homeostasis and consequently contractile function. Interestingly, at concentrations <10 nM both *in vitro* and in vivo, MitoPQ elicited a robust protection against I/R injury. The similarity with preconditioning-induced protection was supported by showing that an antioxidant abrogated MitoPQ-induced maintenance of cell viability. While these findings indicate that mitochondrial ROS formation is sufficient to generate the entire spectrum of cardiomyocyte responses to I/R, the mitohormesis elicited by MitoPQ appears to further explain the lack of success of antioxidant treatment in clinical settings.

### 2.3 Mitochondrial metabolism and cardioprotection

It is widely accepted that the mitochondrial tricarboxyl acid (TCA) cycle metabolite succinate significantly accumulates during tissue ischemia and is now seen as a hallmark of ischemia [52]. This phenomenon is not limited to acute myocardial infarction in the heart; it is observed in various conditions characterized by I/R injury as a core mechanism of tissue damage, including organ transplantation [358], stroke [359], and resuscitation [360]. Furthermore, succinate accumulation is not exclusive to ischemic conditions but also occurs during less specific stressors like exercise, hypothermia, and inflammation [[361], [362], [363]]. Therefore, mitochondrial metabolites are central to pathophysiology and represent a potentially useful therapeutic target.

**Fig. 10:**
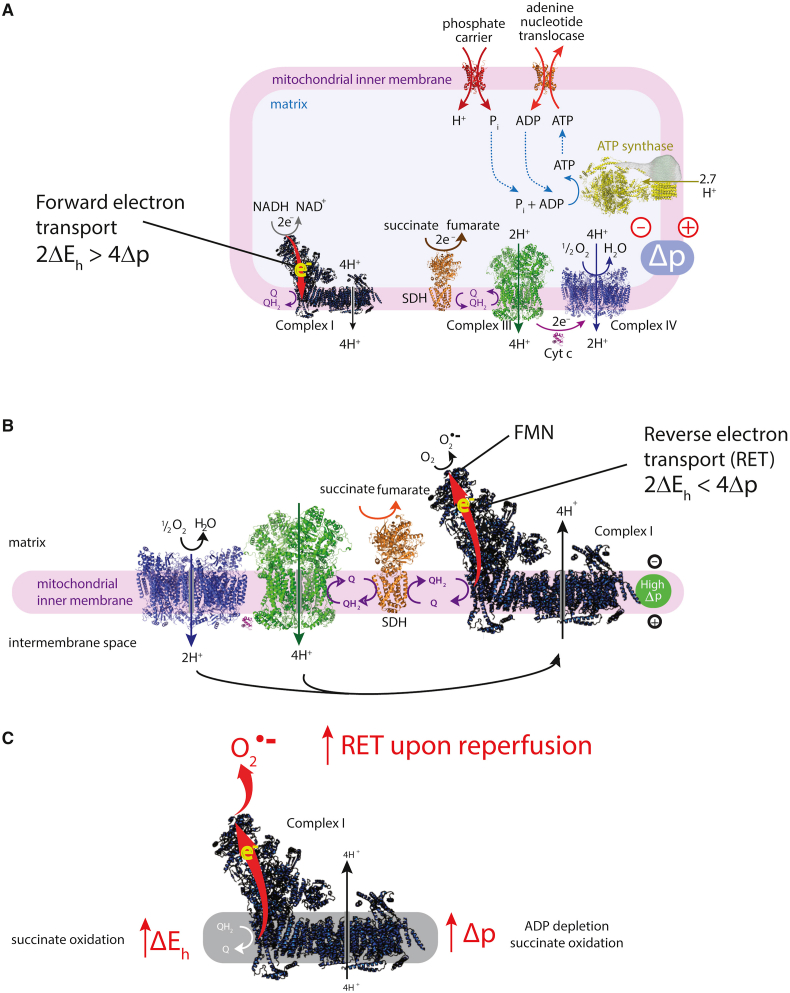
Superoxide Production by Complex I during Reperfusion Injury. (A) Operation of complex I in the forward direction oxidizing NADH in order to generate a protonmotive force (Δp) to be used to synthesize ATP. For forward electron transport to occur the difference in reduction potential between the NAD^+^/NADH and the Coenzyme Q (CoQ) pool across complex I (ΔEh) has to be sufficient to pump protons across the mitochondrial inner membrane against the Δp. As four protons are pumped for every two electrons that pass through complex I, 2ΔEh > 4Δp is the requirement for the forward reaction to occur. The red arrow in complex I indicates forward electron transport. SDH, succinate dehydrogenase. (B) RET by complex I. When the Δp is large and/or the ΔEh across complex I is low such that 4Δp > 2ΔEh, electrons can be driven backward from the CoQ pool onto the FMN of complex I, reducing the FMN which can donate a pair of electrons to NAD^+^ to form NADH, or pass one electron to oxygen to generate superoxide. The red arrow in complex I indicates RET. (C) The factors that favor RET at complex I during reperfusion. The condition to be met for RET to occur is that 4Δp > 2ΔEh. The rapid oxidation of the succinate that accumulates during ischemia favors reduction of the CoQ pool, thereby maintaining a large ΔEh. The reduced CoQ pool also favors proton pumping by complexes III and IV helping maintain a large Δp upon reperfusion. In addition, the degradation of adenine nucleotides during ischemia limits ADP availability upon reperfusion that would otherwise diminish Δp by stimulating ATP synthesis. From Ref. [367] with permission.

The lack of oxygen, the terminal electron acceptor, coupled with the low tissue pH in ischemic conditions, leads to a reduced coenzyme Q (CoQ) pool and the generation of large amounts of succinate. During ischemia, succinate dehydrogenase (SDH) operates in reverse mode, utilizing electrons from the CoQ pool to reduce fumarate into succinate [364,365]. The fumarate may be generated by multiple sources including the degradation of AMP, which accumulates during ischemic events and is provided via the purine nucleotide cycle in the cytosol [366]. However, there is no identified transporter of fumarate, thus it is likely that the fumarate is first converted to malate and transported into mitochondria by the dicarboxylate transporter (DIC). Furthermore, cytosolic malate may also be generated by the transamination of aspartate [367]. To achieve high tissue succinate levels, succinate is transported into the cytosol by the DIC, where it can exchange for malate, bringing in further substrates to maintain the production of succinate [367]. The detailed sequence of events is displayed in Fig. 10.

When reperfusion is initiated by unblocking the culprit artery through interventions such as primary angioplasty in AMI or mechanical thrombectomy in stroke, the accumulated succinate is rapidly transported from the cytosol into the mitochondria by the DIC and oxidized by SDH and reaches normoxic levels within a few minutes [52,359]. Simultaneously, the electron transport chain resumes its function, and together with succinate oxidation maintaining a reduced CoQ pool, the initiation of proton pumping by complexes III and IV generates a high proton motive force (Δp) [368]. These two conditions, the reduced CoQ pool and high Δp, give rise to a phenomenon known as reverse electron transport (RET). RET is known to produce the proximal ROS superoxide at the flavin site of mitochondrial complex I [369]. The mechanism of RET produces the highest amount of superoxide in mitochondria. Although RET was initially believed to occur only *in vitro*, the drastic conditions in ischemic tissue enable its occurrence in vivo, establishing RET as the primary source of mitochondrial ROS production during I/R injury (Fig. 10).

Downstream, likely in conjunction with increased calcium levels, ROS are ultimately responsible for tissue damage through the mitochondrial PTP. When the PTP modulator Cyclosporin A is administered in vivo, the succinate increase during ischemia remains unaffected, suggesting that the RET/ROS mechanism at complex I indeed precedes the detrimental PTP opening [370]. Therefore, ischemia-accumulated succinate drives superoxide production by RET upon reperfusion which initiates the damage in I/R injury.

Blocking various stages of the aforementioned mechanism has shown profound protection against I/R injury in various models, diseases, and species. Inhibiting superoxide production by RET by targeting complex I with rotenone or MitoSNO has demonstrated cardioprotective effects [371,372]. However, prolonged inhibition of complex I disrupts electron flow through the electron transport chain, and studies have shown that mice with genetically reduced complex I activity develop severe cardiomyopathy [373].

Therefore, a more promising approach is to target succinate metabolism [374]. By inhibiting SDH, this can prevent succinate accumulation during ischemia or its oxidation during reperfusion. Malonate, a potent, naturally occurring competitive SDH inhibitor, can effectively block succinate accumulation when administered before or during the ischemic event [375]. While this is less useful in AMI where the ischemic window is unpredictable, this may be particular useful in situations such as organ transplantation or elective surgery where the onset of ischemia is known. More relevant for cardioprotection, malonate can also be given on reperfusion where it slows succinate oxidation. By slowing succinate oxidation, this prevents succinate maintaining a reduced CoQ pool and removes a driving force for RET to occur, preventing I/R injury [52].

In addition to there being sufficient succinate oxidized to produce superoxide by RET on reperfusion, some of the accumulated succinate effluxes from cardiomyocytes. In the heart, approximately 50% of the accumulated succinate is released into the circulation [366]. Patients with STEMI exhibit a significant increase in succinate release from the heart immediately upon reopening of the culprit artery through primary angioplasty [376].

Succinate export into the circulation occurs through the monocarboxylate transporter 1 (MCT1) [377]. Intriguingly, MCT1 is the same transporter responsible for the uptake of exogenous malonate when used as a cardioprotective agent during reperfusion. While malonate is a dicarboxylate at physiological pH and thus poorly diffuses across cell membranes, its monocarboxylic form is favored under low pH conditions [378,379]. As heart tissue reaches pH as low as pH 6 during ischemia, malonate uptake via MCT1 is highly efficient under these conditions. Additionally, an acidic formulation can further enhance this uptake, making acidic malonate an ideal candidate for cardioprotection against I/R injury at reperfusion [378]. In fact, infarct size reduction by intracoronary malonate has been demonstrated in pigs [380,381].

Once in the circulation, succinate represents the intriguing possibility of mitochondrial metabolites acting as a signal of ischemic events. Furthermore, the succinate may be involved in the subsequent mechanisms downstream or distal from the injury site, such as inflammation, or even remote organs. The precise function of the succinate receptor (SUCNR1) is not yet fully understood but may serve as the missing link in understanding these effects [382,383].

Thus, the example of succinate during I/R injury highlights the significance of mitochondrial metabolites as regulators of tissue damage and protection. Further research is necessary to unravel the regulation of mitochondrial metabolites in cardioprotection and to identify promising drug targets.

### 2.4 Mitochondrial reactive oxygen species, calcium and cardioprotection

#### 2.4.1 Mechanisms

**Fig. 11:**
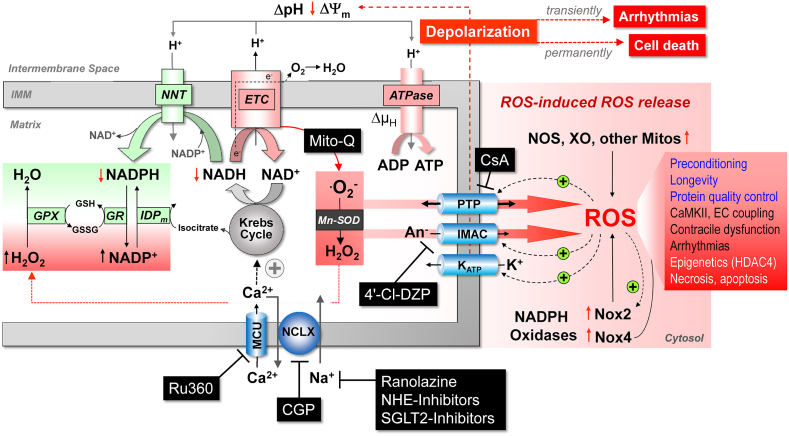
Regulation of mitochondrial respiration and redox state by ion handling. The Krebs cycle is stimulated by Ca^2+^ that enters mitochondria via the mitochondrial Ca^2+^ uniporter (MCU) and is exported by the mitochondrial Na^+^/Ca^2+^-exchanger (NCLX). The Krebs cycle produces NADH, which donates electrons to the electron transport chain (ETC). Sequential redox reactions along the ETC establish a proton gradient (ΔpH) across the inner mitochondrial membrane (IMM) which together with the electrical potential (ΔΨ_m_) constitutes the proton motive force (Δμ_H_), which is harnessed by the F_1_/F_o_-ATP synthase (ATPase) to regenerate ATP via oxidative phosphorylation of ADP. During respiration, superoxide (O_2_^−^) is generated at complexes I and III, which are dismutated to hydrogen peroxide (H_2_O_2_) by the Mn^2+^-dependent superoxide dismutase (MnSOD). H_2_O_2_ is then eliminated by glutathione peroxidase (GPX) and the thioredoxin/peroxiredoxin system (not shown). GPX is regenerated by reduced glutathione (GSH), which in turn is reduced by the glutathione reductase (GR), which uses NADPH that is produced by NADP^+^-dependent isocitrate dehydrogenase (IDP_m_) and the nicotinamide nucleotide transhydrogenase (NNT). Reactive oxygen species (ROS) from NADPH oxidases (Nox) 2 and 4, but also xanthine/xanthine oxidase (XO), nitric oxide synthase (NOS) or other mitochondria (Mitos) can activate redox-sensitive ion channels in the IMM, such as the permeability transition pore (PTP), the inner mitochondrial membrane anion channel (IMAC) or the ATP-sensitive K^+^-channel (K_ATP_). Opening of these channels dissipates ΔΨ_m_, requiring accelerated electron flux along the ETC to maintain ΔΨ_m_. This oxidizes NADH and (via reverse-mode NNT) NADPH and thereby, the antioxidative capacity, limiting H_2_O_2_ elimination. ROS can leave mitochondria through the IMAC or PTP and trigger ROS release from neighboring mitochondria. Depending on the concentrations and durations of ROS elevations, ROS can serve protective roles, such as ischemic preconditioning, longevity and/or protein quality control, but at higher concentrations can deteriorate excitation-contraction coupling and induce epigenetic signaling, apoptosis and/or necrosis. When ΔΨ_m_ (transiently or permanently) dissipates, ATP production ceases, which activates sarcolemmal K_ATP_ channels, making the cell inexcitable. Heterogeneities of ΔΨ_m_ in different cardiac myocytes within the myocardium resemble “metabolic sinks” which can induce re-entry mechanisms to induce arrhythmias. In heart failure, elevated cytosolic [Na^+^]_i_ accelerates mitochondrial Ca^2+^ extrusion, which can be ameliorated by inhibiting the NCLX with CGP-37157 (CGP) or lowering [Na^+^]_i_ by inhibitors of the Na^+^/H^+^-exchanger (NHE), of late Na^+^ current (i.e., ranolazine) and as observed for Sodium/Glucose Co-transporter 2 (SGLT2)-inhibitors via inhibiting NHE and/or late Na^+^ current. CsA, cyclosporine A. GSSG, oxidized glutathione. CaMKII, Ca^2+^/calmodulin-dependent protein kinase II; HDAC4, histone deacetylase 4; EC coupling, excitation-contraction coupling. Modified from [398].

The redox state of cardiac mitochondria, that is, the ratio of reduced over oxidized pyridine nucleotides in the mitochondrial matrix, is continuously modulated by the availability of substrates and oxygen for oxidative phosphorylation, intracellular ion movements, and the intrinsic fitness of the organelle. Reducing equivalents derived from the oxidation of glucose and fatty acids via the TCA cycle maintain the proton-motive force that drives ATP production and sustain the regeneration of mitochondrial antioxidant enzymes required for ROS elimination (Fig. 11). Under physiological conditions, these processes are tightly coupled with the ATP demand imposed by excitation-contraction (EC) coupling via calcium (Ca^2+^) and adenosine diphosphate (ADP), which act in parallel to stimulate the TCA cycle and oxidative phosphorylation, respectively, thereby continuously adapting the rate of oxidative metabolism to the rate of ATP turnover in the cytosol [384]. In particular, mitochondrial Ca^2+^ uptake via the mitochondrial Ca^2+^ uniporter (MCU) complex is essential for matching energy supply and demand in cardiac myocytes, since Ca^2+^ activates rate-limiting dehydrogenases, i.e., pyruvate-, isocitrate- and α-ketoglutarate dehydrogenases of the TCA cycle (Fig. 11).

During ischemia, interruption of the supply of nutrients and oxygen to cardiac myocytes stops oxidative metabolism. The consequent shortage of ATP and phosphocreatine (PCr) hinders cellular ion handling and EC coupling. Hydrolysis of ATP derived from glycolysis leads to accumulation of protons (H^+^) and intracellular acidosis [385], which decreases myofilament Ca^2+^ affinity [386]. The acidic intracellular pH is partly compensated by extrusion of H^+^ in the form of weak acids (lactate) and via the sarcolemmal sodium (Na^+^)/H^+^ exchanger (NHE) [387]) but this, together with the decreased activity of the sarcolemmal Na^+^/K^+^ ATPase and Na^+^ influx via non-inactivating Na^+^ channels, elevates intracellular Na^+^ concentration ([Na^+^]_i_) [388]. In turn, high [Na^+^]_i_ increases cytosolic Ca^2+^ concentrations ([Ca^2+^]_c_) because of the decreased *trans*-sarcolemmal Na ^+^ gradient that drives Ca^2+^ extrusion via the Na^+^/Ca^2+^ exchanger (NCX) [389]. Further, elevation of [Ca^2+^]_c_ can be attributed to Ca^2+^ entry via L-type Ca^2+^ channels and impaired Ca^2+^ reuptake into the sarcoplasmic reticulum (SR) due to decreased activity of the SR Ca^2+^ ATPase (SERCA) [390]. Upon reperfusion, physiological pH is restored within a few minutes as the NHE and Na^+^-dependent bicarbonate exchange extrude excess H^+^ to the extracellular space. The increased NHE activity during the first seconds of reperfusion might further aggravate [Na^+^]_i_ overload [391] and lead to even more Ca^2+^ influx via the reverse mode of the NCX.

Mitochondrial Ca^2+^ overload is considered one of the main mechanisms of irreversible mitochondrial damage and subsequent cardiac myocyte death on myocardial reperfusion [392,393]. It is still debated whether Ca^2+^ overload is mediated by Ca^2+^ uptake via the MCU or by the mitochondrial NCX, which represents the primary Ca^2+^ efflux pathway under physiological conditions (Fig. 11), operating in reverse mode during ischemia and the early phase of reperfusion [394]. Mitochondrial Ca^2+^ overload partly dissipates the proton motive force and, above a certain threshold, triggers PTP opening, in particular in combination with oxidative stress [392,395]. Mitochondrial permeability transition is inhibited during ischemia by the acidic cytosolic environment; therefore, PTP opening happens in the early phase of reperfusion, when the intracellular pH is restored [387]. On these grounds, the cardioprotective effects of PostC can be traced to the delayed recovery of intracellular pH [396].

Reperfusion is accompanied by burst-like release of ROS from mitochondria, which is considered an early driver of cardiac myocyte death. Breakdown of purine nucleotides during ischemia produces fumarate, which is converted to succinate by the reversal of SDH (complex II of the electron transport chain) reaction. Upon reperfusion, succinate is rapidly oxidized by SDH, driving reverse electron transport and extensive superoxide production at complex I [52]. Mitochondrial ROS production induces mitochondrial membrane instability and sensitizes to PTP opening [394]. Furthermore, the burst of ROS during reperfusion of ischemic myocardium causes cytosolic Ca^2+^ overload and myocardial “stunning”, which is characterized by diastolic and systolic dysfunction that is - at least to some extent – reversible [182,397].

#### 2.4.2 Redox-optimized ROS balance

Mitochondrial superoxide is efficiently dismutated to H_2_O_2_ by the Mn^2+^-dependent SOD (MnSOD), while elimination of H_2_O_2_ is governed by glutathione peroxidase and the thioredoxin/peroxiredoxin systems, which all require NADPH for regeneration (Fig. 11) [398]. NADPH, in turn, is produced by enzymes that derive their substrates from the TCA cycle, i.e., malic enzyme, isocitrate dehydrogenase and NNT. Therefore, during physiological increases in cardiac workload, mitochondrial Ca^2+^ uptake is required not only to maintain NADH for ATP production, but also of NADPH to detoxify ROS (Fig. 11) [384]. While the primary formation of superoxide is highest when the mitochondrial redox state (and therefore, the ETC) is highly reduced [399] elimination of ROS is compromised when the mitochondrial redox state (and in particular, of NADPH) is strongly oxidized. Therefore, in working cardiac myocytes, the lowest net H_2_O_2_ emission occurs at an intermediate redox state, as expressed by the concept of “redox-optimized ROS balance” [400].

#### 2.4.3 ROS-induced ROS release

A feedforward mechanism to boost mitochondrial ROS emission is the concept of “ROS-induced ROS-release”, where ROS activate redox-sensitive ion channels in the inner mitochondrial membrane, such as the PTP [401], inner mitochondrial anion channels (IMAC) [402,403] and ATP-dependent K^+^ channels (mK_ATP_) [404,405] of the same or neighboring mitochondria(Fig. 11). Activation of these channels dissipates the mitochondrial membrane potential (ΔΨ_m_) and thereby accelerates oxidation of NADH and consequently, of NADPH through the reverse-mode of the NNT [406], depleting the anti-oxidative capacity and provoking ROS emission (Fig. 11). Such ROS-induced ROS release with IMAC activation underlies synchronous oscillations of the mitochondrial membrane potential, creating “metabolic sinks” which makes those areas of the myocardium unexcitable and thereby provides a substrate for re-entrant arrhythmias during cardiac I/R [403]. While opening of the IMAC occurs at low amounts of oxidative stress and is often reversible, activation of the PTP typically occurs at higher amounts of ROS and is rather irreversible, often inducing necrotic cell death [407]. ROS-induced ROS release was also observed through communication between different sources of ROS, such that ROS from NOX2 can increase mitochondrial ROS [405,408,409] and *vice versa*, mitochondrial ROS can increase NOX2-related ROS (Fig. 11) [410].

#### 2.4.4 ROS and mitochondrial K_ATP_ channels

Cardioprotection through IPC requires low amounts of mitochondrial ROS, which can be induced by pharmacological opening of mK_ATP_ channels [333,[411], [412], [413]], Many signaling pathways that induce preconditioning converge onto mK_ATP_ channels [414]. These ROS then trigger protective pathways that eventually reduce the amounts of mitochondrial ROS released during a larger I/R injury [411]. The mere existence and the molecular composition of the mK_ATP_ have been enigmatic for decades; recently, CCDC51 was identified to form a channel with mK_ATP_-like properties when associating with the ATP Binding Cassette protein 8 (ABCB8) [415], which had already been shown earlier to modulate mK_ATP_ activity [416]. Alternatively, mK_ATP_ channel activity has been proposed for F_1_F_0_-ATP synthase based upon its K^+^ conductance [417]. Notably F_1_F_0-_ATPase has been also described as a structural component of the PTP [392,395]. Additional studies are necessary to clarify the role of F_1_F_0_-ATPase in cardioprotection related to mK_ATP_ and PTP (Fig. 11) [418].

Also beyond I/R, mitochondria are considered the major source of ROS in heart failure [384]. In failing hearts, superoxide is generated at complex I and is converted to hydroxyl radical [419,420]. Furthermore, dysregulated cytosolic Ca^2+^ and Na^+^ handling deteriorate mitochondrial Ca^2+^ accumulation and thereby, regeneration of NADH and NADPH in failing cardiac myocytes, which contributes to energetic deficit, oxidative stress, contractile dysfunction and arrhythmias [384]. Furthermore, the mere increase in afterload, which is typical for heart failure, drains anti-oxidative NADPH towards NADH and ATP production, but at the cost of the anti-oxidative capacity, thereby further increasing the emission of H_2_O_2_ from mitochondria [406]. This increase in ROS is causal for the development of necrosis, progressive heart failure and death [404,406,421,422].

#### 2.4.5 Interventions

Based on this mechanistic framework, several therapeutic strategies have been developed and tested in animal models and, in some instances, clinical trials to mitigate cardiac I/R injury. Mitochondrial permeability transition is considered the final common pathway through which dysfunctional mitochondria induce cardiac myocyte death; indeed, PTP inhibition with cyclosporine or genetic ablation of cyclophilin D substantially reduce the sensitivity to I/R injury in rodents [423]. Despite these promising results in animal models and in one phase 2 trial [231], intravenous infusion of cyclosporine immediately prior to reperfusion did not improve 1-year cardiovascular outcomes nor affected left ventricular remodeling in patients with anterior STEMI in a phase 3 randomized controlled trial [232].

Therapeutic strategies targeting processes upstream of PTP opening, i.e. preventing mitochondrial Ca^2+^ overload or dampening mitochondrial ROS, yielded conflicting results. While antioxidant therapy after reperfusion with mitochondria-targeted coenzyme Q (Mito-Q, Fig. 11) reduced infarct size [424], also the inhibition of respiratory chain complexes I [425] or II [426] as well as mild mitochondrial uncoupling [427] were cardioprotective in I/R injury by inhibiting succinate-mediated ROS production. Inhibiting IMAC during I/R with 4′-chlorodiazepam prevented postischemic ventricular arrhythmias [403].

In principle, interventions that reduce the rise in cytosolic Ca^2+^ during I/R also reduce the amount of myocardial injury [428]. Genetic deletion or pharmacological inhibition of the NHE reduced infarct size in mice [429], but did not improve outcome in patients with myocardial infarction [430]. Sodium-glucose transporter 2 (SGLT2) inhibitors reduce hospitalization for heart failure and cardiovascular death in patients with diabetes, chronic kidney disease and heart failure [431,432]. Since the benefits in patients with heart failure are independent of the presence of diabetes, it has been suggested that SGLT2-inibitors directly target the heart. In fact, SGLT2-inhibitors block the NHE [433,434], but also the late sodium current (Fig. 11) [[435], [436], [437]], which is considered the main source of elevated [Na^+^]_i_ in heart failure [438]. In support of such concepts, SGLT2-inhibitors reduced infarct size in vivo in mice with genetic global deletion of SGLT2 [439], which may be related to lowering of [Na^+^]_i_ and consequently, cytosolic Ca^2+^ to prevent mitochondrial Ca^2+^ overload (Fig. 11).

In mice, cardiac myocyte-specific, inducible (but not global constitutive [440] knock-out of the MCU protected against mitochondrial Ca^2+^ overload and PTP opening, reduced infarct size and improved postischemic cardiac function [441]. Also in rats, pharmacological inhibition of the MCU with Ru360 (Fig. 11) improved cardiac post-ischemic functional recovery [442], however, it is unclear whether this effect is indeed related to a reduction in mitochondrial Ca^2+^ accumulation. Since the main uptake of Ca^2+^ into mitochondria during I/R is still controversial, it is relevant to mention that inhibiting the NCX did not reduce infarct size, but efficiently prevented arrhythmias in pigs after myocardial I/R. In guinea pigs with heart failure, NCX inhibition protected from contractile dysfunction, cardiac remodeling and ventricular arrhythmias [443].

### 2.5 Thiol-oxidant signaling in myocardial health and disease

#### 2.5.1 Oxidants are produced by cells and are implicated in disease progression

Cells produce a great variety of molecules capable of oxidatively modifying other components in the system. Although these oxidant molecules are conveniently grouped together under the umbrella terms ROS or nitrogen species (RNS), this is not always helpful as it does not consider the enormously variable chemistry between these entities. This is important to remember because these biochemical reactions underlie the cellular impact of these species, both in the context of oxidative damage as well as the sensing and signalling they can initiate. This illustrates the importance of defining the species of oxidant that is measured and using absolute quantitation, which cannot be readily achieved with fluorescence reporter probes [444]. Measuring specific oxidants is notoriously difficult, not least because their reactivity often makes their existence fleeting, although their lifetime varies enormously between molecular species, cellular location and environmental conditions. Nevertheless, the anticipated high reactivity of oxidants is a cornerstone of the paradigm that they causally mediate disease by oxidizing and thus damaging the fabric of the cell. Of course, as we will come to below, oxidation events may not be solely synonymous with damage and instead may represent passive or indeed oxidant sensing events that mediate homeostatic signal transduction.

A common scenario is for a study to report that ROS, RNS or markers of their presence are increased in samples from patients or disease models compared with healthy controls. However, these measurements of oxidant abundance or indirect markers of oxidative stress could simply reflect epiphenomena that are not causative in the pathogenesis. Indeed, it has been questioned how useful it is for our community to keep measuring oxidants levels [445], especially when it is often done in a way that does not define the molecular species and does not use absolute quantification with reference standards. Although oxidants can mediate homeostatic or protective signaling, they continue to be routinely implicated as important, causal mediators of all manner of diseases, including those of the cardiovascular system. Causality has mostly been established through the use of antioxidants, with a plethora of preclinical studies showing these interventions alleviate both the oxidative stress and improve function. Studies with transgenic models in which oxidant producing or scavenging enzymes were modulated provided more evidence that oxidants were pathogenic. Furthermore, tool compounds or drugs that inhibit oxidant-generating enzymes have proven therapeutic. Thus, it seems abundantly clear that ROS or RNS can causally mediate cardiovascular disease at multiple levels, including inflammation, atherosclerosis, hypertension and heart failure [446]. Cardiac ischemia and hypoxia are often associated with increased ROS, but there is complexity and controversy as there is evidence that the decrease in oxygen availability decreases oxidant levels [447]. This may relate to reductive stress, which is recognized in the pathogenesis of cardiovascular pathologies. The pentose phosphate pathway generates the reductant NADPH used by multiple antioxidant enzyme systems to scavenge oxidants, but it is also used for anabolic membrane and nucleic acid synthesis and is causatively upregulated during pathological cardiac hypertrophy [448].

ROS contribute to LV dysfunction [449] in mice with transgenic overexpression of ROS formation [450], in rabbits with pacing-induced heart failure [451], in pigs with coronary microembolization [452] and in dogs with postischemic contractile dysfunction (stunning) [453,454]. Post-ischemic reperfusion, which is essential for tissue survival, resupplies oxygen and is associated with oxidant-induced reperfusion injury that in countless pre-clinical studies has been attenuated by antioxidants.

#### 2.5.2 Sources of ROS and the susceptibility of sulfur containing targets to oxidation

ROS and RNS-induced damage were often considered indiscriminate because they oxidise most biomolecules in their path. However, we now understand oxidant production can be localized - constrained to specific organelles or signalling complexes. Sources of oxidants include, but are not limited to, mitochondria [52], NOX [455], xanthine oxidoreductase, MAOs or uncoupled nitric oxide synthase (NOS) or inducible NOS generating nitrosating entities [446]. Localized production is anticipated to constrain and therefore limit the number of targets that are oxidatively modified, a scenario that is more compatible with regulated oxidant signalling. Further potential for regulation comes from some targets being preferentially susceptible to oxidation, which together with the selective reactivity of oxidants enables fidelity and regulation.

Many amino acids in proteins can be oxidized, but the sulfur containing amino acids methionine and especially cysteine are preferentially susceptible. The cysteine thiol (−SH) is not intrinsically reactive with oxidants. However, in some proteins the proton can be donated to an acceptor (e.g., the side chain of lysine, arginine or histidine) to electrostatically stabilize the thiolate (-S^-^) state, which is very reactive and enables them to form oxidative post-translational modifications. There is substantial diversity in the post-translational modifications that may occur on protein thiols, varying with the oxidant encountered and its abundance and duration of exposure.

#### 2.5.3 Redox sensitive proteins cysteines in cardiac disease and protection

Vast numbers of proteins are now known to be regulated by various oxidative post-translational modifications of cysteines, which can impact cellular homeostasis and disease pathogenesis. Indeed, the Oximouse study reported tens of thousands of reversibly modified cysteines with significant oxidation stoichiometry that was enabled by their proximal amino acids [456]. Ageing, as with major diseases, has been repeatedly touted to be mediated by oxidative damage. However, the Oximouse study found no widespread increase in protein cysteine oxidation with age and notably, the sites with a high modification stoichiometry were more reduced to the thiol state in older mice. This finding resonates with a continually emerging literature, together with the considerations above, that oxidants are not simple perpetrators of disease but also facilitate signalling that enable homeostasis during health or enable protective adaptation during disease.

Whilst oxidation of proteins, including their sensitive cysteine residues, has been implicated in I/R injury [457], it is notable that oxidants are important triggers of IPC and this cardioprotective process is blocked by antioxidants [458]. In addition to ROS or RNS production during the protection phase initiating signaling that limits injury, they induce *S*-nitrosation [459] as well as disulfides [458] of protein cysteines that may protect them from irreversible, harmful over-oxidations during subsequent longer damaging periods of I/R. *S*-nitrosation of mitochondrial complex I at a specific cysteine has been shown to be cardioprotective against IR by decreasing their production of ROS [425]. The redox state of specific cysteine residues in proteins including protein kinase A RIα, protein kinase GIα, soluble epoxide hydrolase and optic atrophy 1 impact broadly on cardiovascular physiology and disease outcomes [457,460,461].

#### 2.5.4 Cardiovascular protection by pro-oxidants and electrophilic drugs

It is clear that the redox state of cardiovascular tissues is perturbed both during health and disease-related scenarios. Although most of the focus has been on oxidants causing pathologies, it is notable as considered above, in some scenarios, tissues instead become more reduced, that antioxidants are not therapeutic against human cardiovascular disease and that IPC protection is associated with induction of protein oxidation. Furthermore, relatively unselective pro-oxidant interventions that are anticipated to induce widespread oxidation of protein thiols can attenuate infarction during IR [372,458]. Nitroxyl (HNO), a reduced derivative of nitric oxide, is a potent unselective thiol oxidizer generated endogenously and donors of which are showing therapeutic promise against heart failure, improving systolic and diastolic function, and as an anti-hypertensive [460]. This cardioprotection afforded by a drug that donates an RNS is a notable example of how such species do not simply mediate dysfunction. However, the complexity is highlighted by endogenous *S*-nitrosating species derived from inducible NOS causing heart failure with preserved ejection fraction by inducing the *S*-nitrosation of endonuclease inositol-requiring protein 1α [446]. Endogenous production of hydrogen peroxide by NOX4 protects the heart from hypertrophic growth [455]. There are other classes of electrophilic drugs that induce protein cysteine oxidation that are used clinically (e.g., dimethyl fumarate) or have regulatory approval for human clinical trials such as the stabilized sulforaphane derivative and SFX-01 and nitro-alkenes, consistent with a growth and significant advances in the development of drugs that target and covalently modify cysteines in proteins [462].

How to reconcile protection from these oxidant interventions from the robust contradictory evidence that they mediate disease is not straight forward. However, if we consider there are tens of thousands of redox active protein cysteines [456]. We can understand that the net outcome of an oxidative stress will be scenario-specific, with different profiles and outcomes from protein oxidation depending on the source and species of oxidant, as well as their abundance and duration of supply. Some protein oxidation events will be neutral, some beneficial, and others detrimental, and the net outcome will have to be considered on a case-by-case basis. Early studies with pan-specific kinase inhibitors, have now given way to molecules with high target precision and we do not typically consider global tissues phosphorylation or dephosphorylation to be ‘good or bad’, whereas historically oxidation is mostly synonymous with ‘bad’. We understand phosphorylation to be an important and complex regulatory mechanism that depending on the specific situation can mediate both health maintenance as well as disease progression, and the same can be applied to oxidative post-translational modifications. Clearly, we already have electrophilic drugs, mentioned above, in clinical use or development that are not very selective and modify many targets, including those that do not mediate the therapy and may cause side effects. In the same way as highly specific kinase inhibitors have been developed that overcome the off-target effects of broad-spectrum drugs, this can be achieved for specific cysteine residues in a protein [462]. Indeed, FMK was engineered to selectively inactivate p90 RSK1/2, a kinase that can mediate myocardial dysfunction, by covalently adducting a conserved cysteine thiol in their C-terminal kinase domain to prevent ATP utilization [463]. Furthermore, thiol-reactive drug G1 was developed that selectively induces the oxidation of protein kinase G Iα cysteine 42, targeting and activating the kinase to lower blood pressure in hypertensive mice [464]. It is anticipated that a variety of new drugs will eventually emerge that selectively target a specific regulatory cysteine residue in a protein to provide new therapies, including for diseases of the cardiovascular system.

### 2.6 Hydrogen sulfide and cardioprotection

Hydrogen sulfide (H_2_S), an endogenous signaling molecule, has been recognized, in the last decades as an important endogenous gasotransmitter that influences several important (patho-)physiological processes related to cardiovascular health and disease [465,466]. Endogenous H_2_S is as an important regulator of the cardiovascular system, particularly of myocardial function [467] and its upregulation may reduce ischemic injury [468]. Findings on the endogenous synthesis of H_2_S and its protective role in cellular necrosis, apoptosis, oxidative stress and inflammation in myocardial I/R injury have encouraged interest in the development of new therapies based on facilitation of endogenous H_2_S for cardioprotection [469].

H_2_S is a second messenger implicated in protection from oxidative stress by direct scavenging of ROS and indirect antioxidant effects [470]. H_2_S is generated mainly by cystathionine γ-lyase (CSE) in cardiovascular tissues [468]. In CSE knockout rats subjected to I/R injury, oxidative stress was aggravated, whereas increased expression of H_2_S and CSE in aortic tissues resulted in alleviation of oxidative stress accompanied by reduced expression of apoptosis-related proteins [471]. H_2_S produced by CSE improves contractile function in heart failure, and treatment with *S*-propyl-l-cysteine (SPRC) or sodium hydrosulfide (NaHS), modulators of blood H_2_S levels, attenuated the development of heart failure, reduced lipid peroxidation, and preserved mitochondrial function in mouse models [472]. However, CSE gene deletion does not substantially exacerbate the long-term response to myocardial infarction, and the H_2_S donor GYY4137 when administered after the onset of myocardial infarction preserved cardiac function and protected against adverse cardiac remodeling in both WT and CSE-deficient mice [468].

Conditioning-like infarct limitation by enhanced level of H_2_S has been demonstrated in many animal models of myocardial I/R injury in vivo [473]. Numerous studies have shown that H_2_S has a significant protective role in myocardial I/R injury and is considered an important mediator of IPC, through different mechanisms which include: i) activation of mitochondrial potassium channels, ii) reduction of oxidative stress and activation of endogenous antioxidant mechanisms, iii) limitation of inflammatory responses, iv) preservation of mitochondrial function, v) angiogenic actions, vi) interaction with NO, and vii) *S*-sulfhydration [467,[474], [475], [476]]. H_2_S is also involved in postconditioning-induced cardioprotection through multiple mechanisms, including redox-based post-translational modification on protein cysteine residue(s), i.e. *S*-sulfhydration (SSH) and preservation of mitochondria [477,478]. Since endogenous concentrations of H_2_S are generally low, the necessity for an exogenous source of H_2_S provides a unique challenge for the development of chemical tools that facilitate the study of H_2_S under biological conditions. H_2_S donors include a wide variety of functional groups and delivery systems, some of which mimic the tightly controlled endogenous production in response to specific, biologically relevant conditions [reviewed in] [479]. The most common class of H_2_S donors employed in biological studies are the sulfide salts, sodium hydrosulfide (NaSH) and sodium sulfide (Na_2_S) which in many in vivo studies reduced myocardial infarct size and conferred cardioprotection when administered prior or during reperfusion [470,[480], [481], [482]]. A mitochondria-specific H_2_S compound, AP39, protects against I/R injury, an effect mediated through inhibition of PTP opening via a cyclophilin D-independent mechanism [483]. Τhioester-based H_2_S donors release H_2_S in a slow and controllable manner that could mimic its slow-releasing process in vivo. The donor 5e reduced myocardial infarct size and cardiomyocyte apoptosis in mice [484]. A macromolecular H_2_S prodrug grafts 2-aminopyridine-5-thiocarboxamide (a small-molecule H_2_S donor) on partially oxidized alginate (ALG-CHO). This drug formulation then mimics the slow and continuous release of endogenous H_2_S and tetra-aniline (a conductive oligomer) and adipose-derived stem cells (ADSCs) and forms a stem cell-loaded conductive H_2_S-releasing hydrogel. After myocardial injection, longer ADSCs retention and elevated sulfide concentration in rat myocardium were demonstrated, accompanied an increased ejection fraction and smaller infarct size [485]. Isothiocyanate-based H_2_S-releasing drugs decreased I/R-induced tissue injury in an in vivo model of acute myocardial infarction in rats [467]. The newly designed H_2_S-releasing ibuprofen derivative, BM-88, reduced infarct size in the ischemic/reperfused myocardium in isolated rat hearts [486]. Furthermore, reactive sulfur species, including RSSH and polysulfides, exhibit cardioprotective actions [487].

Since multitarget cardioprotective therapies can possibly more effectively translate cardioprotection to patients, there are some hybrid molecules that may have 2 or more structural domains and act as 2 distinct pharmacophores to provide additive cardioprotection [57]. A hybrid compound that combines the adenine nucleus with a moiety that slowly releases H_2_S induced additive infarct size reduction in anesthetized rabbits [488]. H_2_S and NO share a wide range of physical properties and physiological functions that not only affect each other's biosynthesis but also produce novel species through chemical interaction and play a regulatory role in the cardiovascular system through similar signaling mechanisms or molecular targets [489]. Based on the above, therapeutic strategies that increase both H_2_S and NO have emerged such as ZYZ-803, a hybrid molecule of a dual donor for H_2_S and NO which preserved cardiac function and reduced infarct size significantly after 24 h left coronary artery ligation through reversing an H_2_S and NO imbalance in mice [490].

**Fig. 12:**
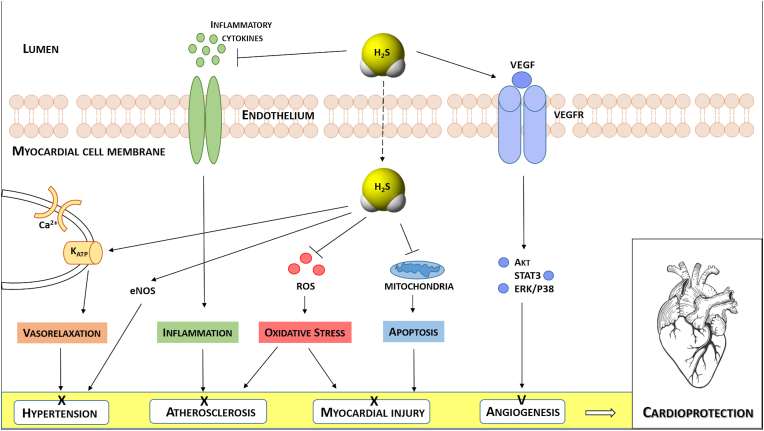
Schematic illustration of the effects of H_2_S in different heart diseases and the molecular mechanisms underlying H_2_S-induced cardioprotection. From Ref. [492] with permission.

Multiple targets for H_2_S-releasing drugs have been identified for pre- and postconditioning, such as PTP, K_ATP_, PKC, eNOS/cGMP, ERK/GSK3β, VEGF/JAK2/STAT-3/iNOS signaling, attenuation of ROS, SSH on protein cysteine residue(s) [477], and mitochondrial preservation, indicating that H_2_S may attenuate irreversible I/R injury [491,492]. A summary of these major cardioprotective pathways initiated by H_2_S is provided in Fig. 12. In a meta-analysis, preconditioning with H_2_S caused an infarct limitation of - 20-25% and postconditioning limited infarct size by - 21-61% and this infarct-sparing effect was robust and consistent when H_2_S was applied before ischemia or at reperfusion, independently of animal species or sulfide source [473].

However, most of the studies investigating the cardioprotective effects of H_2_S have been performed in small animal I/R injury models. For translation to the clinical situation of large animal I/R injury models are important [493], but only few studies have evaluated the cardioprotective effects of H_2_S in large animal models. H_2_S infusion initiated at the onset of ischemia and continued into the reperfusion period, but not bolus administration over 10 s at the start of reperfusion markedly reduced myocardial infarct size, improved regional left ventricular function, as well as endothelium-dependent and endothelium-independent microvascular reactivity in Yorkshire pigs [494]. Treatment with sodium sulfide 10 min prior to the onset of reperfusion improved myocardial function, reduced infarct size and improved coronary microvascular reactivity potentially through anti-inflammatory properties in Yorkshire pigs [495]. Zofenopril, a sulfhydrylated angiotensin-converting enzyme inhibitor increased H_2_S and NO bioavailability via bradykinin-dependent signaling, reduced myocardial infarct size and cardiac troponin I levels after IRI when administered 7 days before 75 min ischemia and 48 h reperfusion in pigs [496].

More recently, several studies also focused on the cardioprotective effects of H_2_S in the presence of co-morbidities. H_2_S ameliorated I/R injury in different animal models of diabetic cardiomyopathy (DM) through different pathways that regulate apoptotic, autophagic, necroptotic, and pyroptotic cell death [497,498]. Preconditioning with H_2_S reduced infarct size in isolated rat hearts with diabetes and DM [499]. Divergent results exist on H_2_S postconditioning in diabetes; H_2_S reduced infarct size in isolated diabetic rat hearts [500], whereas in other studies H_2_S post-conditioning failed to induce cardioprotection in diabetic rat hearts, mainly due to their altered myocardial architecture along with exacerbated oxidative stress and mitochondrial dysfunction [500]. Obesity increases the risk of developing diabetes and subsequently DM. Reduced cardioprotective and antioxidant H_2_S and increased pyroptotic cell death contribute to adverse cardiac remodeling and DM, and exercise training prevented the development of DM possibly by promoting H₂S-mediated cardioprotection and attenuating pyroptosis in mice [501]. Cardioprotection is effective in young hearts but is lost in aged hearts [96]. Exogenous H_2_S restored postconditioning-induced cardioprotection through decreasing infarct size and apoptosis, improving cardiac function, and increasing cell viability and autophagy in aged hearts and cardiomyocytes. Additional mechanisms involved up-regulation of HB-EGF/EGFR signaling, which activates the ERK1/2-*c*-myc and PI3K-Akt- GSK-3β pathways in aged cardiomyocytes of rats and in H9C2 cardiomyocytes [502,503].

All the above observations have inspired the rapid development of H_2_S-releasing compounds for clinical translation to patients with cardiovascular disease. The H_2_S prodrug SG-1002 has been evaluated in a phase I clinical trial [ID: NCT01989208] in healthy and heart failure subjects, showing attenuation in the increases in BNP in heart failure patients [504], but it is not used in the treatment of heart failure patients because of its lack of a stable, controllable H_2_S booster [505]. Despite its promises from preclinical studies, H_2_S has not been evaluated in humans. The Groningen Intervention study for the Preservation of cardiac function with sodium thiosulfate (STS) after STEMI (GIPS-IV) used a double-blind, randomized, placebo-controlled, multicenter trial design and enrolled patients with a first STEMI who received the H_2_S-donor sodium thiosulfate in addition to standard care immediately at arrival in the catheterization laboratory. Unfortunately, this trial was not completed according to the original power-analysis based study design, and all results including the primary endpoint infarct size after 4 months by CMR were neutral [506].

In summary, H_2_S has emerged as an important cardioprotective molecule with potential for clinical applications. H_2_S-donors as pro-drugs able to generate exogenous H_2_S, are viewed as promising therapeutic agents for a number of cardiovascular diseases. However, the influence of co-morbidities and co-medication has not been really studied in most preclinical studies, and data from humans are limited or absent.

### 2.7 Protective effects of NADPH oxidases on the heart

NOXs are a family of multi-subunit transmembrane enzymes that generate ROS (either superoxide anion [*O_2_^−^] and/or [H_2_O_2_] through NADPH-dependent reduction of molecular oxygen. ROS generation is a primary function of the NOXs, in contrast to most biological enzymes (e.g. the complexes of the mitochondrial electron transport chain or nitric oxide synthases) which have other primary functions. NOXs are now recognized to be especially important in mediating specific redox signaling in diverse physiological and pathophysiological contexts, a property that relates to NOX-mediated ROS production typically being physiologically regulated and spatially compartmentalized within or outside cells [[507], [508], [509]]. The combination of finely regulated ROS production (on a temporal and quantitative level) and proximity between ROS source and target(s) underpins highly specific molecular signaling in many cases. Of particular relevance to cardioprotection, NOXs have significant potential to mediate adaptive or protective responses through such signaling, in stark contrast to non-specific detrimental effects associated with high level ROS production and oxidative stress. The NOX family of enzymes comprises 7 isoforms (NOX1-5 and DUOX1-2) among which NOX2 and NOX4 are significantly expressed in the heart and are the focus of this section.

NOX2 was initially identified more than 30 years ago as the phagocytic oxidase responsible for high-level ROS production (the “oxidative burst”) during neutrophil phagocytosis. It is a complex enzyme that when inactive is formed of a membrane-bound heterodimer between the catalytic subunit NOX2 (also known as gp91^phox^) and a smaller p22^phox^ subunit. Activation of the enzyme involves post-translational modifications of several cytosolic subunits (p67^phox^, p47^phox^, Rac1 or Rac2, and p40^phox^) induced by intracellular signaling cascades, followed by their translocation to the membrane to associate with the NOX2-p22phox heterodimer and the triggering of electron transfer from NADPH bound to NOX2 to molecular oxygen on the other side of the membrane. We now know that NOX2 is expressed in numerous non-phagocytic cells including cardiomyocytes, endothelial cells and fibroblasts where it is involved in intracellular redox signaling [[510], [511], [512]]. Cardiomyocyte NOX2 is located predominantly on the plasma membrane and T-tubules and is activated by G-protein coupled receptor (GPCR) agonists (such as angiotensin II, endothelin-1), cytokines, growth factors and mechanical forces [513]. NOX2-mediated redox signaling in cardiomyocytes (and other non-phagocytic cells) appears to be underpinned by endosomal signaling and a much lower level of ROS production than in neutrophils.

**Fig. 13:**
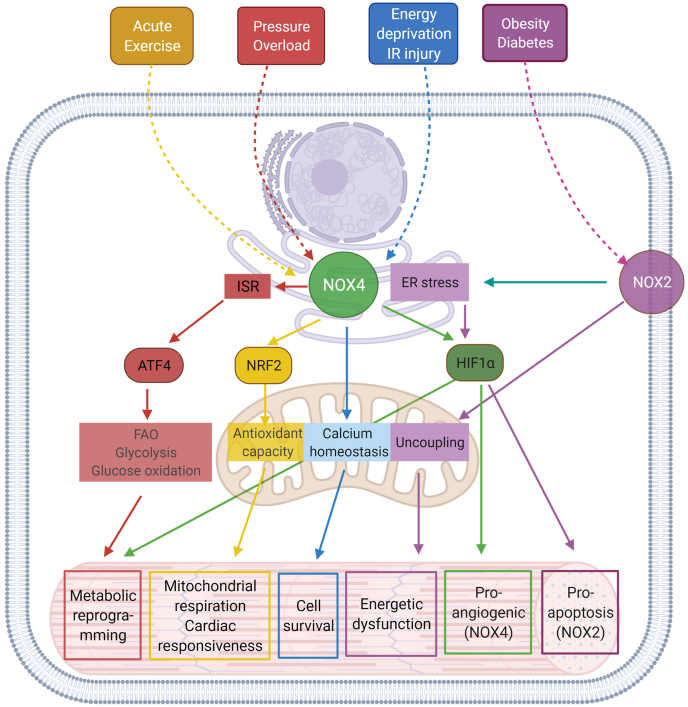
Roles of NADPH oxidases (NOX) in physiology and pathophysiology. Both NOX2 and NOX4, the main NOX isoforms in cardiomyocytes, can regulate intermediary metabolism in response to a variety of stresses. Several mechanisms are involved, including activation of transcription factors or in the case of NOX4 location at the MAM, targeted ROS signaling to influence mitochondrial function and cell viability. ATF4, activating transcription factor 4; NRF2, nuclear factor erythroid factor 2-related factor 2; HIF1α, hypoxia-inducible factor 1-alpha; ISR, integrated stress response. From Ref. [516] under the Creative Commons license.

NOX4 is a deceptively simple isoform that like NOX2 exists as a membrane-bound heterodimer with a p22^phox^ subunit but exhibits constitutive low-level ROS production and does not require cytosolic subunits for activation. Other striking differences from NOX2 are its intracellular location at the endoplasmic reticulum (ER) and the production of H_2_O_2_ rather than *O_2_^−^ as its main ROS product [514]. NOX4 has a very wide (possibly ubiquitous) tissue distribution and appears to be regulated predominantly by its protein abundance [507]. It is highly inducible by numerous stressful stimuli such as ischemic, metabolic, neurohumoral and mechanical cues, secondary to both transcriptional activation (notably by ATF4) and post-transcriptional mechanisms [513,515]. NOX4 has attracted considerable attention because of its substantial potential to mediate adaptive (protective) signaling in the heart and elsewhere, underpinned by its involvement in several fundamental stress pathways [513] A summary of these adaptive and maladaptive pathways initiated by NOX isoforms is provided in Fig. 13 [516].

#### 2.7.1 NOX2-dependent adaptive and maladaptive pathways

The vast majority of studies on NOX2 in the heart have reported that it augments pathological cardiac remodeling in response to stresses such as chronic neurohumoral activation, chronic hemodynamic overload and acute myocardial infarction [513]. Many different components of the pathological remodeling phenotype are enhanced by NOX2, including cardiomyocyte hypertrophy, cardiomyocyte cell death, abnormal excitation-contraction coupling, arrhythmia, contractile dysfunction, inflammation, interstitial fibrosis and ventricular dilatation. These effects typically involve the NOX/redox-mediated amplification of responses such as kinase signaling, transcription factor activation, aberrant Ca^2+^ release from the SR, and/or matrix metalloprotease activation. Genetic studies that employed cell-specific manipulation of NOX2 levels in mice have also established that different NOX2-expressing cells in the heart – including cardiomyocytes, endothelial cells, fibroblasts and immune/inflammatory cells – make distinct contributions to these responses [513].

More recently, it has become evident that NOX2 also has a number of physiological actions. Prosser et al. [517] showed that cardiomyocyte NOX2 physiologically tunes stretch-induced release of Ca^2+^ from the SR and thereby optimizes length-dependent augmentation of contractile performance. Physiological increases in contractile performance in response to acute angiotensin II stimulation are also amplified by NOX2, this time through an enhancement of SR Ca^2+^ uptake [518]. Another adaptive role for NOX2 is reported to be an involvement in myocardial ischemic preconditioning which was found to be blunted in mice globally deficient in NOX2 [519]. NOX2 is capable of activating hypoxia-inducible factor 1α (HIF1α) [520], which might also contribute to adaptive responses although direct investigation of this remains limited.

#### 2.7.2 NOX4-dependent adaptive pathways

Initial studies that investigated the role of NOX4 in the heart using gene-modified mouse models generated controversy because opposite effects (protective or detrimental) in the setting of chronic pressure overload were reported by the 2 laboratories undertaking independent studies [521,522]. With time, however, compelling evidence has accumulated that NOX4 mediates protective effects in the heart under diverse stress situations; even the group that initially reported detrimental effects subsequently observed protective features [513,523]. Moreover, protective effects of NOX4 against disease stress are also observed in many other tissues. It has become evident that part of the explanation for detrimental effects of NOX4 in some disease models relates to very high levels of induced NOX4 overexpression and/or the disruption of coupling to signaling pathways essential for its protective effects [524].

##### 2.7.2.1 NOX4-dependent activation of HIF1α

Studies employing global and cardiomyocyte-targeted mouse models of NOX4 deletion or NOX4 overexpression revealed that an increase in NOX4 levels during chronic pressure overload was beneficial by reducing the magnitude of cardiomyocyte hypertrophy, contractile dysfunction, interstitial fibrosis and chamber dilatation [521]. An increase in endogenous NOX4 levels was found to be essential for activation of a HIF1α -vascular endothelial growth factor (VEGF) axis that preserves myocardial capillary density and cardiac contractile function during chronic hemodynamic overload, consistent with prior reports of the importance of this axis in this setting. NOX4 both in cardiomyocytes and endothelial cells appears to contribute to these effects [525]. It is suggested that the mechanism of HIF1α activation by NOX4 involves a ROS-dependent inhibition of the prolyl hydroxylase that regulates HIF1α stability. NOX4-dependent HIF1 activation has also been reported to contribute to cardioprotective effects during ischemia but the mechanisms downstream of HIF1α that mediate protection require more study. Interestingly, protective effects of NOX4-dependent HIF1α activation are also reported in the peripheral vasculature and kidneys.

##### 2.7.2.2 NOX4-dependent activation of NRF2

Nuclear factor erythroid 2 related factor 2 (NRF2) is well known to be activated by oxidative or electrophilic stress – which result in the oxidation and targeting of the NRF2 inhibitor, KEAP1, for proteosomal degradation – but recent studies indicate a unique obligatory role for endogenous NOX4 in the activation of NRF2 in the heart in several settings. NRF2 upregulation in response to hemodynamic overload was abrogated in global NOX4 knockout mice and contributed to the detrimental phenotype in these animals [526]. In cultured cardiomyocytes, phenylephrine-induced increases in NRF2 levels were dependent on NOX4 but not NOX2. An even more striking finding is that exercise-induced physiological induction of NRF2 in the heart in mice is absolutely dependent on NOX4. Using several different genetic models including cardiomyocyte-specific NOX4 or NRF2 knockout mice, it was definitively demonstrated that endogenous NOX4 in cardiomyocytes activates NRF2 during physiological free-running wheel exercise and plays a critical role in optimizing cardiac contractile function and maximizing exercise capacity [527]. The effects downstream of NRF2 that mediate these beneficial effects both during physiological and pathological stress are of considerable interest. NRF2 is well known to be a master regulator of antioxidant and cytoprotective responses by inducing diverse genes that regulate glutathione biosynthesis, intracellular redox state, phase II antioxidant enzymes and a wide variety of metabolic pathways. Studies to date indicate an important role for NRF2 in maintaining optimal cytosolic and mitochondrial redox state during stress [526,527]. In addition, metabolic effects consequent to NRF2 induction may also be important in the protective effects. At a conceptual level, it is notable that there appears to be an integral reciprocal relationship between NOX4-dependent NRF2 signaling and redox state (akin to the concept of hormesis), opposite to the conventional paradigm that ROS production associates with oxidative stress. How NOX4 mediates this specific role requires further study but it may be noted that NOX4-mediated activation of NRF2 is observed in several other cell types and organs [455].

##### 2.7.2.3 Regulation of ATF4 and the integrated stress response by NOX4

The integrated stress response (ISR) is an evolutionarily conserved adaptive pathway that responds to multiple stresses including amino acid and glucose deprivation, hypoxia, oxidative stress and ER stress. The central component of this pathway is the phosphorylation of the α-subunit of the eukaryotic initiation factor 2 (eIF2α) by one of four kinases, each responsive to different stress stimuli, resulting in a global inhibition of protein translation but enhanced cap-independent translation of a subset of mRNAs including the transcription factor ATF4. ATF4 activation induces multiple genes involved in amino acid import and metabolism, redox balance, ER chaperone functions, autophagy and intermediary metabolism, a repertoire which usually mediates adaptive functions in response to stress [528]. ATF4 activation is subject to negative regulation via the dephosphorylation of eIF2α by a sub-fraction of protein phosphatase 1 (PP1), which is targeted to the ER by a PP1-targeting subunit GADD34. NOX4 specifically enhances eIF2α phosphorylation and ATF4 translation during stress, secondary to an interaction with GADD34 and redox inhibition of PP1 at the ER [515]. NOX4-mediated enhancement of ATF4 induces strong protective effects against acute ischemic cardiac injury as well as in a model of acute renal tubular necrosis. Moreover, NOX4 itself is a transcriptional target of ATF4 [515], resulting in an additional positive feedback mechanism, and probably explaining why so many stress stimuli increase NOX4 levels.

The specific mechanisms downstream of NOX4-dependent enhancement of ATF4 signaling that mediate protection against ischemia or other stresses remain to be fully elucidated. An enhancement of cardiomyocyte autophagy is suggested to be important in the NOX4-ATF4-dependent protective response to cardiac ischemia [523]. NOX4-dependent protection against hemodynamic overload-induced contractile dysfunction may in part involve an alteration in cardiac substrate utilization. Cardiac-specific NOX4 overexpression leads to a reduction in myocardial glucose oxidation rate, an increase in fatty acid oxidation rate and a well-preserved energetic state during chronic pressure overload [529]. The mechanism underlying the increase in fatty acid oxidation appears to be the post-translational *O*-GlcNAcylation (addition of *N*-acetylglucosamine) of the fatty acid membrane transporter CD36, which regulates fatty acid uptake by the myocardium. The increase in *O*-GlcNAcylation is driven by an ATF4-dependent increase in expression of glutamine-fructose-6-phosphate transaminase 1 (GFAT1), the rate-limiting enzyme in the hexosamine biosynthetic pathway (HBP) – a glycolytic branch pathway that mediates protein *O*-GlcNAcylation. Therefore, NOX4-dependent upregulation of ATF4 links increased flux into the HBP with an increase in fatty acid oxidation to preserve energetic balance.

##### 2.7.2.4 NOX4-mediated inhibition of Ca^2+^ transfer from ER to mitochondria

A final protective mechanism involving NOX4 that was recently discovered is the regulation of ER-to-mitochondrial Ca^2+^ transfer during severe stress. It is well established that severe stresses (such as ischemia) may induce a significant transfer of Ca^2+^ from ER to mitochondria, resulting in a triggering of the mitochondrial PTP and cell necrosis [530]. NOX4 is highly concentrated at the junctions between ER and mitochondria (mitochondrial-associated membrane, MAM) in multiple tissues and its levels are significantly increased at this location during severe cell stress [531]. NOX4 enhances Akt-dependent phosphorylation of inositol 1,4,5-trisphosphate receptors (InsP_3_R) at the MAM by inhibiting PP2A (which normally dephosphorylates InsP_3_R). The consequence of increased InsP_3_R phosphorylation at the MAM is an inhibition of stress-induced Ca^2+^ release via these channels to mitochondria and an inhibition of PTP-dependent cell necrosis. This mechanism was found to be strongly protective against cardiac I/R injury [531].

These studies indicate that NOX4 mediates adaptive effects in the heart in response to diverse stresses and involving several different redox-regulated mechanisms. Elucidation of the detailed signalling underpinning these pathways may lead to novel therapeutic options, involving an enhancement of such adaptive pathways.

### 2.8 Crosstalk between sources of reactive oxygen species

**Fig. 14:**
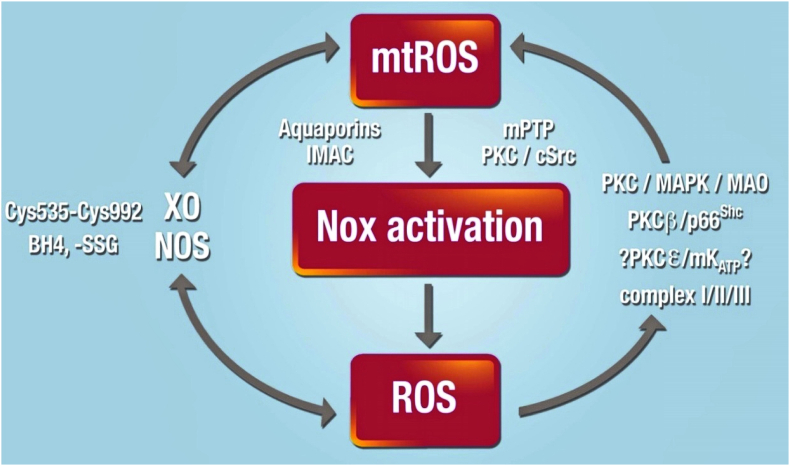
Crosstalk between different sources of RONS: mitochondria, NADPH oxidase (NOX), xanthine oxidase (XO) and uncoupled NOS. XO originates from oxidative stress-mediated conversion of the xanthine dehydrogenase via oxidation of critical thiols in cysteine535/992. NOS (mainly eNOS) are uncoupled upon oxidative depletion of BH4, *S*-glutathionylation (-SSG), adverse phosphorylation by protein kinase C (PKC) and other redox switches [535]. Mitochondrial O_2_^•^‾/H_2_O_2_ formation is triggered by oxidative stress from all ROS sources (including other damaged/activated mitochondria) via redox-activation of PKC, mitogen-activated protein kinases (MAPK), other kinase pathways and potential involvement of redox-sensitive mtK_ATP_ with subsequent p66^Shc^, monoamine oxidase (MAO), respiratory complex activation or impairment of mitochondrial antioxidant defense [352]. Mitochondrial O_2_^•^‾/H_2_O_2_ is released to the cytosol via mitochondrial pores and channels (e.g. redox-sensitive mPTP, inner membrane anion channel (IMAC) or aquaporins) or by diffusion due to increased mitochondrial permeability under pro-inflammatory conditions. In the cytosol these species (along with released calcium) cause activation of redox-sensitive PKC and tyrosine kinases (cSrc) with subsequent activation of NOX and amplification of the cellular oxidative stress [410]. Adapted from Ref. [352] with permission.

Since mitochondria and NADPH oxidases are both involved in ROS formation during I/R, there is a vital crosstalk between these ROS sources and one can stimulate the other, as reported for a broad set of disease and cellular stress conditions [532]. The concept of “ROS-triggered ROS formation” was first reported for self-amplified mitochondrial ROS formation, visualized by waves of enhanced ROS levels along mitochondrial networks [401]. According to this hypothesis damaged mitochondria produce ROS that initiate ROS formation by neighboring mitochondria. Later, similar crosstalk was also reported for interaction of NOX and mitochondria in angiotensin II (AT-II) mediated preconditioning [533]. Protection by AT–II–mediated preconditioning was blocked by the NOX inhibitor apocynin, and blockade of the mtKATP in cardiac myocytes by 5-hydroxydecanoate (5-HD). In an editorial to this original paper, Brandes proposed that cytosolic ROS generated by NOX can stimulate mitochondrial ROS formation [534]. In general, the concept of “kindling radicals” (or also “bonfire” hypothesis) explains the activation of secondary ROS sources and functional damage of redox-regulated enzymes such as eNOS but also redox-driven conversion of xanthine dehydrogenase to its oxidase form [535]. There, the kindling radicals (most likely from NADPH oxidases or mitochondria) cause oxidative conversion of eNOS or xanthine dehydrogenase to ROS-producing source enzymes (see “redox switches” in Fig. 14) [352]. The concept of the interaction (crosstalk) of different ROS sources was developed to explain the observation that pharmacological inhibition or genetic deletion of one specific ROS source is in many disease models enough to confer a complete normalization of the disease phenotype (see numerous examples for hypertension and AMI in reference) [352].

Whereas the majority of reports for this redox crosstalk was published for the NOX2/mitochondrial axis in the setting of hypertension [410,536,537], it was also observed in nitrate tolerance, a nitroglycerin-induced oxidative stress condition [538] and the aging process [410,[538], [539], [540]]. Especially the role of CypD, a small redox sensitive regulator of the PTP, in the crosstalk of mitochondrial ROS and NOX2-dependent ROS formation is meanwhile well established in AT-II induced hypertension, by prevention of most adverse effects in CypD knockout mice [410,541]. Cysteine 203 in CypD determines the activity of the PTP regulator CypD, and therefore represents a redox switch of PTP which confers higher opening probability of the pore under oxidative stress conditions [542]. In contrast, *S*-nitros(yl)ation of cysteine 203 prevented H_2_O_2_-induced PTP opening, identifying nitric oxide as an antagonist of ROS in this redox process, which may be the process of nitroglycerine-mediated cardioprotection that was lost on cyclophilin D knockout mice [543].

I/R damage is based on mitochondrial ROS formation as a central pathophysiological mechanism [[544], [545], [546]]. Rathore et al. reported a mechanism by which mitochondrial ROS activate PKCε (prevented by chelerythrine and PKCε deletion) with subsequent increase in NOX activity (prevented by apocynin and p47^phox^ deletion) in the setting of hypoxia as a model of I/R damage (e.g. as observed in AMI or stroke) [547]. The authors showed that hypoxia activates most likely NOX1 isoform in pulmonary arteries, as documented by translocation of p47^phox^ to the plasma membrane. The involvement of mitochondrial ROS formation in this process was proven by lower NADPH activity in GPx-1 overexpressing mice and higher NADPH activity in GPx-1 knockout mice. The crosstalk concept was meanwhile extended to all common ROS sources to explain reperfusion damage [548]. Oxidative stress in general and this crosstalk in particular have also large impact on cellular calcium homeostasis and mitochondrial function in the diabetic heart [549], similar to the Ca^2+^/ROS crosstalk previously described in cancer development and progression [550] and cellular function per se [551].

### 2.9 Cardioprotective exosomes/extracellular vesicles – is there a role for redox mechanisms?

**Fig. 15:**
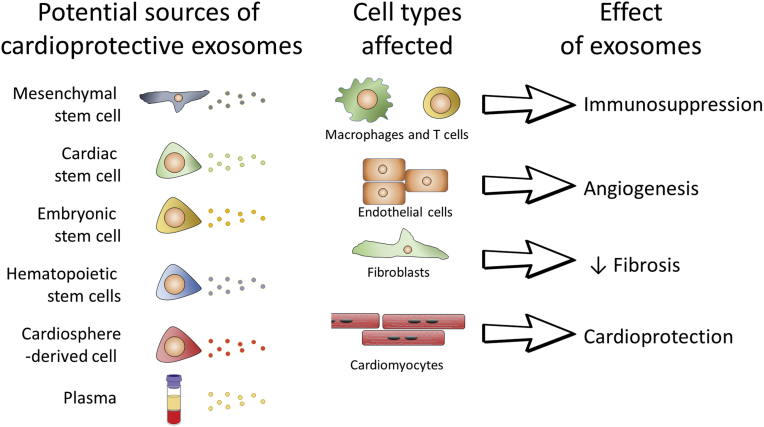
Effects and sources of exosomes. (upper part) Some of the major effects that have been reported of exosomes that are relevant to the ischemic heart, and the cell types that have been reported to be involved in the effect. See text for details. (lower part) The different potential sources of exosomes discussed in this review. Although each type of cell has certain unique characteristics, the exosomes they produce are notable for the consistent array of effects they induce. From Ref. [553] with permission.

Exosomes are a type of small extracellular vesicle (EV) that have been determined to have multiple beneficial effects on recipient cells and organs [552]. Like other EVs, exosomes are surrounded by a lipid bilayer, and contain proteins, microRNAs (miRNAs) and other non-coding RNAs originating from the cytosol. All cell types appear to have the machinery to produce and release exosomes, although the extent to which they do so appears to vary both on cell type and physiological state [552]. Most cells can also produce microvesicles, which are somewhat larger EVs with different characteristics from exosomes. However, since it is challenging to isolate highly pure exosomes, they are usually referred to experimentally as small EVs (sEV). The bulk of sEVs in the blood originate from erythrocytes and platelets, although sEVs originating from the endothelium and other organs can also be identified. Several studies have demonstrated the potential for plasma sEVs to protect the heart from I/R injury [552]. However, the bulk of interest has focused on stem and progenitor cells cultured *in vitro* as a more practical source of potential cardioprotective sEVs. Irrespective of the type of stem or progenitor cell, the sEVs they produce appear to be protective both in the acute setting where they can reduce the size of infarct following I/R, and in the chronic setting, where they are able to improve cardiac remodeling, reduce fibrosis, increase angiogenesis, and improve cardiac contractile function in the weeks following I/R or chronic myocardial ischemia [552]. As yet, there is no consensus as to the mechanism of cardioprotection by sEVs, as it also depends on their cargo molecules that includes proteins as well as coding and non-coding RNAs. Many studies interested in the chronic setting of infarction and heart failure are interested in examining the role of miRNAs, since these have the potential to remodel transcriptional and translational networks over that time scale. With regards to acute cardioprotection, where the majority of the infarct is believed to be formed acutely after reperfusion, it seems more likely that sEVs activate one of the known cardioprotective kinase signalling pathways in the heart via interaction with surface receptors, or alternatively by delivery of cytoprotective proteins from sEVs into cardiac cells. Cellular calcium overload and oxidative stress are well established as major mediators of I/R injury. One might therefore hypothesize that sEVs exert part of their cardioprotective capabilities via antioxidant mechanisms. Surprisingly, the redox activity of sEVs has not received a great deal of attention. Nevertheless, there are several relevant studies suggesting that sEVs are redox-active. A summary of the cellular sources and their cardioprotective effects is provided in Fig. 15 [553].

#### 2.9.1 EVs and redox modulation of endogenous cardioprotective mechanisms

IPC increases the rate of release of sEVs from isolated perfused rat hearts and hypoxia/reoxygenation similarly stimulates EV release from cultured endothelial cells. When these sEVs are added to cardiomyocytes *in vitro*, they increase their resistance to injury [552]. Furthermore, preconditioned hearts released sEVs that protected naïve hearts from I/R injury when transferred via the perfusate [552]. This suggested that sEVs may mediate the inter-organ signalling pathway of RIC. A subsequent study found that in both rats and humans, RIC increased the number of sEVs in the blood, although unexpectedly, plasma sEVs isolated from either naive or preconditioned individuals were found to be similarly protective when administered intravenously to rats undergoing IR [552]. In contrast, Minghua et al. found that RIC-induced exosomes attenuated infarct size and improved heart function when administered intramyocardially, with evidence that EV-delivery of miR-24 was able to reduce oxidative stress, at least in H_2_O_2_-treated H9c2 cells *in vitro* [552].

Another stimulus known to improve cardiovascular health and limit injury following I/R is exercise training. Exercise alters the cargo of circulating sEVs, increasing their antioxidant function by stimulating glutathione reductase and catalase activities. Furthermore, the exercise-induced sEVs can reduce oxidative damage in human iPS-derived cardiomyocytes subject to oxidative stress [554]. sEVs derived from ticagrelor-pretreated H9c2 significantly decreased hyperglycemia-stimulated ROS production and prevented apoptosis [555].

Oxidative stress has been implicated in the pathogenesis of metabolic disease-induced (diabetes or hyperlipidemia) cardiovascular complications [556]. Many cardioprotective modalities including IPC and RIC are impaired or ineffective in the setting of diabetes [556]. RIC was ineffective in diabetic Zucker fatty rats, and an exosome-rich sample from the diabetic rats was unable to protect cardiomyocytes from hypoxia/reoxygenation-induced death [557]. Similarly, sEVs from diabetic rats had lost the ability to protect cardiomyocytes. Importantly, however, protection could be restored by using exosomes from a healthy, non-diabetic source [558].

In certain circumstances such as sepsis, sEVs from platelets may even be detrimental. Exposure of platelets to NO or lipopolysaccharides caused the release of redox-active exosomes containing nNOS and NOX, which generated superoxide and NO. This caused endothelial cell apoptosis via formation of peroxynitrite [559], and induced myocardial dysfunction in isolated rabbit heart and papillary muscle preparations [560]. On the other hand, seemingly toxic sEVs are not always necessarily damaging. Energetically stressed adipocytes released EVs containing respiration-competent, but oxidatively damaged mitochondrial particles. These were taken up by cardiomyocytes, where they triggered a burst of ROS resulting in compensatory antioxidant signalling that protected cardiomyocytes from subsequent acute oxidative stress – somewhat reminiscent of preconditioning [561].

#### 2.9.2 Exogenous EVs and redox modulation of cardioprotective mechanisms

The first report that MSC-derived exosomes could reduce infarct size in mice subject to I/R was by Lai et al., in 2010 [562]. In a subsequent publication they showed that increased myocardial viability and cardiac function in this model corresponded with increased levels and ATP and NADH, decreased oxidative stress and activated PI3K/Akt pathway in the heart [552].

Since then, sEVs from many different stem and progenitor cell sources have been shown to be cardioprotective, but MSC remain of particular interest due to their apparent beneficial immunomodulatory effects of the sEVs the produce [552]. There is some evidence that they have redox-mediated effects. For example, bone marrow-derived MSC decreased ROS production and apoptosis in cardiac stem cells after oxidative stress injury [563]. Interestingly, the effect was greater with those EV isolated from MSC cultured in hypoxic conditions. The mechanism was proposed to be via transfer of miRNA-214 to suppress its target, CaMKII.

In a proteomic analysis, sEVs from human vascular endothelial cells were found to contain proteins associated with redox state, calcium handling and cellular metabolism. Importantly, analysis of human cardiomyocytes after exposure to the sEVs revealed corresponding changes in these proteins, suggesting their cargo was delivered within the cells. Treatment with the sEVs increased the respiratory capacity of normoxic cardiomyocytes and reduced tissue damage in a human heart-on-a-chip I/R injury model [564].

EVs may modulate redox signalling between cells of the heart. For example, treatment of cardiomyocytes with Tongxinluo (a traditional Chinese medicine approved in 1996 by the State Food and Drug Administration of China for treating angina pectoris and ischemic stroke), causes them to release sEVs containing the lncRNA Linc-ROR. When the sEVs are taken up by cardiac endothelial cells, linc-ROR downregulates miR-145–5p leading to activation of eNOS. The NO increases survival in both the endothelial cells and cardiomyocytes [565].

Another type of cardiac injury involving ROS is chemotherapy-induced cardiotoxicity. For example, doxorubicin/trastuzumab-induced cardiac toxicity increases ROS in cardiomyocytes. Intravenous administration of cardiac progenitor cell-derived exosomes prevents ROS and protects against doxorubicin/trastuzumab-induced cardiac toxicity [552]. In this case, the sEVs were highly enriched in miR-146a-5p and suppressed target genes in doxorubicin-treated cells, including NOX4 and myeloperoxidase, both major ROS-producing enzymes [552]. Interestingly, sEVs are no longer beneficial when they are obtained from inflamed hearts. Instead, they increase ROS production by NOX, thereby reducing NO bioavailability in treated cardiac endothelial cells [566]. Thus, the functional effects of sEVs can depend greatly on the health of the cells of origin.

Ageing-associated vascular dysfunction involves oxidative stress. With aging, primary cells undergo senescence, during which they cease proliferation, become pro-inflammatory and produce higher levels of ROS. This increases the production of sEVs [567]. sEVs from endothelial progenitor cells down-regulated the NOX2/ROS pathway by delivery of miR-18a, thereby protecting them from hypoxia and reoxygenation injury [567]. Interestingly, a population of microvesicles from endothelial cells contained enzymes and substrates of the pentose phosphate pathway leading to their ability to synthesize NADPH, which is a key metabolite in antioxidative pathways. Thus, they may act as ROS scavengers [567]. This pathway was even more active in senescent endothelial cells. On the other hand, when human umbilical vein endothelial cells were subject to hypoxia/reoxygenation, the microvesicles they produced induced the phosphorylation of p38 and JNK1/2 and promoted apoptosis and oxidative stress in H9c2 cardiomyocytes [567].

The cargo of sEVs is rich in small non-coding RNAs such as miRNAs. miRNAs have been associated with both cardioprotection and also with regulation of redox signalling of the heart. In a recent study of miRNA-mRNA interaction of I/R and oxidative stress-induced alterations followed by microRNA-mRNA target interaction, network analysis revealed that microRNAs and their mRNA targets that may play a role in cardioprotection via alterations in redox signalling [568].

In summary, there remains great interest in sEVs as cardioprotective agents, and there is suggestive evidence in the literature that part of their benefit may be mediated through redox-regulated pathways. Although there is no consensus as to their main mechanism of action, this may be a reflection of the multi-factorial nature of the sEVs, which contain an assortment of proteins, lipids and miRNAs that may all contribute to the overall benefit. This is in line with the proposal that a multi-target strategy may be required for successful clinical translation of cardioprotection [57].

### 2.10 Cardioprotective cytokines and growth factors

One of the problems that underlies many cardiac disorders is the incapacity of the heart to undergo regeneration after damage. Measurements by ^14^C-carbon dating [569], imaging mass spectrometry [570] and analysis of DNA synthesis [571] are concordant in showing a minimal regenerative capacity of the adult mammalian heart, in the order of 1% cardiomyocyte renewal per year. This is well below what would be required to compensate for pathological loss. Thus, the goal of developing treatments that protect cardiomyocytes from death after acute I/R injury or more chronically in other forms of cardiac disease has paramount importance.

Studies performed over 2 decades ago have already shown that various growth factors, including insulin-like growth factor-1 (IGF-1), hepatocyte growth factor (HGF), endothelin-1 (ET-1), fibroblast growth factor-2 (FGF-2), and transforming growth factor-β (TGF-β) can protect the heart against oxidative stress (reviewed in Ref. [572]). These growth factors stimulate intracellular signal transduction pathways that converge on the activation of protective kinases, in particular PI3K/AKT and MAPKs, which eventually protect cardiomyocytes from oxidative stress-induced apoptosis [572]. Other cardioprotective cytokines against I/R injury include macrophage migration inhibitory factor (MIF), which contributes to the regulation of cardioprotective AMPK (reviewed in Ref. [573]), IL-6 [574,575], irisin (a myokine produced by FNDC5 cleavage) [576,577] and follistatin like-1 (FSTL-1) [578,579]. Several of these cytokines are physiologically expressed by the skeletal muscle or heart after injury and are part of the endogenous protective mechanism against I/R injury, which can be enhanced for therapeutic purposes [580]. Other cytokines are produced by different leukocytes populations. Among these, myeloid-derived growth factor (MYDGF) is highly upregulated in both human and mouse infarcts and is predominantly expressed by CXCR4^+^ monocytes and macrophages [581]. Treatment with this recombinant cytokine was shown to reduce scar size and contractile dysfunction after AMI by inducing AKT activation followed by reduction of apoptosis, while also promoting cardiac endothelial cell proliferation [581].

Recent work performed by the Giacca laboratory has tackled the issue of identifying cardioprotective secreted molecules in a systematic, unbiased manner and independent of whether these cytokines and growth factors are physiologically expressed in the heart after injury. Through an AAV-based method that functionally selects for tissue protective molecules [582,583] and the use of a cDNA library corresponding to the mouse secretome, this work systemically ranked a collection of about 1200 cDNAs coding for secreted factors for their cardioprotective efficacy [584]. After two rounds of iterative, in vivo selection, the most effective cytokines in this functional screening were Chrdl1, an inhibitor of bone morphogenic protein (BMP) [585], Fam3c/ILEI, a metabolic regulator that takes part in various biological functions [[586], [587], [588]] and Famb3b/PANDER, which is normally expressed in the pancreas and participates in the regulation of glucose homeostasis and β-cell function [[589], [590], [591]]. None of these three cytokines are known to take part in the normal cardiac response to injury but are effective when pharmacologically administered to the heart.

Several of the above mentioned cardioprotective cytokines act by blunting oxidative damage after myocardial infarction, and their effect can thus be scored by visualizing viable myocardium immediately after I/R. However, several others also act by different mechanisms that are equally essential to determine longer term cardiomyocyte survival and cardiac function. These mechanisms include, among others, the extent of perfusion and collateral flow induction, efficiency in removal of dysfunctional mitochondria, blunting of excessive inflammation, and activation of intracellular metabolic pathways. These protective mechanisms are activated several hours after the acute ischemic events, and thus cannot be properly assessed immediately after reperfusion, as they act on a vast number of cardiomyocytes in the so called “area-at-risk” [592], which is significantly larger than the area that eventually becomes infarcted [593]. A more straightforward manner to assess the efficacy of cardioprotection, which also considers cardiomyocyte survival in these areas of uncertain fate after reperfusion, is by analyzing the effects of treatments over time. For this purpose, CMR not only provides information on the extent of early damage after ischemia reperfusion [594], but, also and most notably, on the evolution of myocardial infarction and cardiac function at later time points.

In terms of the mechanisms underlying cardioprotection, multiple studies have shown that autophagy and, more specifically, the autophagic recycling of malfunctional mitochondria (mitophagy) exert an essential protective effect after ischemia/reperfusion to prevent later pathological remodeling and the development of heart failure [595,596]. Aberrant mitochondrial function not only results in inadequate energy production but, more relevant, augments the generation of reactive oxidative species [597]. Originally identified in yeast [598], mitophagy is a universal autophagic response that specifically targets these potentially cytotoxic mitochondria (reviewed in Refs. [599,600]). Not surprisingly, several treatments that reduce cardiac damage, including after I/R, also modulate mitophagy (reviewed in Ref. [601]). In a consistent manner, all the three cytokines identified in the above mentioned, systematic, AAV functional selection screening are also powerful inducers of autophagy [584], as it is ghrelin, a 28 amino acid peptide that was previously identified as protective for skeletal muscle and heart by a similar in vivo selection approach [583].

In light of the information obtained over the last years on the effect of molecules that act extracellularly to protect cardiomyocytes from death, the development of an injectable cytokine as a therapeutic molecule for acute or chronic cardioprotection appears to be an attainable goal.

## 3 Conclusions and perspectives

**Fig. 16:**
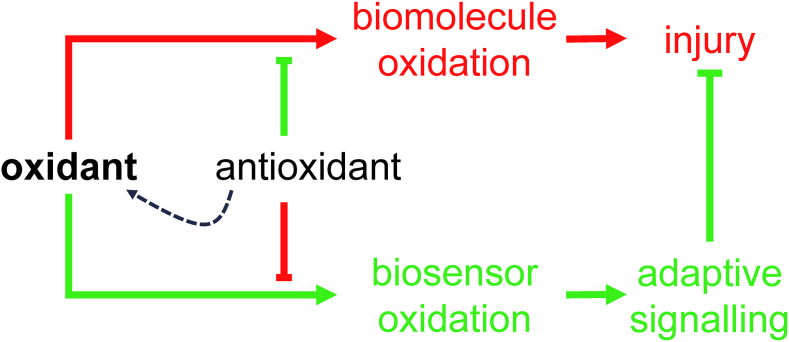
ROS and RNS are commonly thought to induce injury by oxidizing (i.e., damaging) the cellular fabric, including proteins, lipids and nucleotides. There is significant pre-clinical evidence that oxidants are damaging based on protection by antioxidants, but this has not translated though to human studies with large-scale clinical trials often showing no benefit or sometimes adverse outcomes. This could be because antioxidants scavenge oxidants that otherwise initiate protective signaling events. As electron donors, antioxidants have the potential to fuel oxidants generation as shown by the dotted line, which adds further complexity. Another consideration is that oxidized antioxidants can exert biological actions via their pro-oxidant chemistry.

Rapid reperfusion is mandatory to salvage myocardium from I/R injury but there is a continued need for cardioprotection beyond that by rapid reperfusion. A number of mechanical and pharmacological interventions have reduced infarct size and coronary microvascular obstruction in preclinical and clinical proof-of-concept studies but their translation to better clinical outcome of patients has been largely disappointing such that further research on mechanisms of I/R injury and its attenuation is needed [35]. ROS play a decisive role on I/R injury and its attenuation, both as damaging agents and as cardioprotective signals (Fig. 16). There continues to be significant advances in understanding mechanisms of ROS production during I/R [52] and how these mediate dysfunction [457]. Perhaps these new insights may offer opportunities for therapies that are more successful than the antioxidant interventions that failed to translate, as will now be discussed in the next section. Given the multitude of preclinical studies showing antioxidants are protective, including against cardiovascular conditions, it seems obvious that their administration to patients should be therapeutic. Whilst some clinical trials have demonstrated antioxidants protect against cardiovascular disease, this has not been widely replicated with the outcomes of large-scale clinical trials showing supplementation is not beneficial. Indeed, supplementing cardiovascular disease patients with antioxidants worsens outcomes in terms of all-cause mortality, as it has in other diseases such as cancers [460]. In the same way that ROS and RNS are a diverse array of molecules with often markedly different chemistry, the same is true for antioxidants, with differing abilities to react with and so neutralize the supposedly damaging oxidants (Fig. 16). Little consideration is typically given to the fact that cells are replete with antioxidant systems and whether supplementation of a human with small tablets of antioxidant can significantly or sufficiently bolster the endogenous defenses against oxidant species. In addition, oxidation of antioxidants may in some cases generate pro-oxidant molecule that initiate biological actions, including those that may be beneficial to the cardiovascular system [602,603]. Furthermore, as antioxidants are electron donors, they can potentially generate oxidant species to counterintuitively induce oxidative stress.

The concept that diseases caused by oxidative stress can be corrected with antioxidants or inhibitors of the enzymes that synthesize these perceived perpetrators, is perhaps attractive because of the seeming simplicity of the paradigm for which there is so much pre-clinical evidence. Furthermore, natural antioxidants are often considered safe, perhaps because they are present in our diets, and they are also readily available. The oxidative damage concept is arguably widely grasped by the lay public with advertisements for antioxidant supplements for aging and all manner of conditions enabling a multibillion-dollar annual market. However, whether the lay public are aware of the evidence against the use of antioxidants in disease and significantly understand the basic principles of redox processes is questionable.

The dual role of ROS, beneficial and detrimental, is shared by other processes, such as intracellular [Ca^2+^] elevation and PTP opening. Indeed, preconditioning-like protection has been obtained by increasing extracellular Ca^2+^ [604], while CypD deletion abolished IPC-induced protection [605]. Furthermore, translating experimental acute *vs* prolonged effects should be considered carefully. For instance, suppression of mitochondrial ROS generation by mitochondrial-targeted catalase hampered autophagy worsening outcome in a model of heart failure [606], Similarly, the transition from hypertrophy to failure was shown to be exacerbated by PTP inhibition [607]. Therefore, the impetuous flow of knowledge in basic science will provide real benefits in clinical settings only if all aspects are considered thoroughly.

## Funding

GH is supported by the German Research Foundation (CRC 1116, B8) and the 10.13039/501100000780European Union (COST ACTION CARDIOPROTECTION CA 16225).

IA is supported by by the Operational Program “Competitiveness, Entrepreneurship and Innovation"2014–2020 under the call “RESEARCH – CREATE - INNOVATE” (project codes: 03427 and 5048539) and the 10.13039/501100000780European Union (COST ACTION CARDIOPROTECTION CA 16225).

HEB was supported by Independent Research Fund Denmark (0134–00294B), 10.13039/501100009708Novo Nordisk Foundation (NNF19OC0058543, NNF18OC0052789) and the 10.13039/501100000780European Union (COST ACTION CARDIOPROTECTION CA 16225).

SMD acknowledges the support of the 10.13039/501100000274British Heart Foundation (BHF PG/16/85/32471, PG/18/44/33790, PG/19/51/34493, PG/21/10798).

PE is supported by The 10.13039/100015652Barts Charity Cardiovascular Programme Award G00913 and programme grants from the 10.13039/501100000274British Heart Foundation and the 10.13039/501100000265Medical Research Council.

PF was supported by the National Research, Development and Innovation Office of Hungary (Research Excellence Program TKP within the framework of the Therapeutic Development thematic program of the Semmelweis University; National Heart Laboratory (RRF-2.3.1-21-2022-00003). PF is a vice chair of the COST CIG (IG16225) and an MC member of the COST CardioRNA project (CA17129).

MG was supported by the 10.13039/501100000781European Research Council (ERC) Advanced Grant 787971 “CuRE”; 10.13039/501100000274British Heart Foundation (BHF) Programme Grant RG/19/11/34633; King's College London BHF Centre of Research Excellence grant RE/18/2/34213; grants 825670 “CardioReGenix” and 874764 “REANIMA” from the 10.13039/501100000780European Commission Horizon 2020 programme.

DH is supported by the 10.13039/100016017Duke-NUS Signature Research Programme funded by the Ministry of Health, Singapore Ministry of Health's National Medical Research Council under its Singapore Translational Research Investigator Award (MOH-STaR21jun-0003), Centre Grant scheme (NMRC CG21APR1006), and Collaborative Centre Grant scheme (NMRC/CG21APRC006). This article is based upon work supported by the 10.13039/501100000921COST Action EU-CARDIOPROTECTION IG16225 supported by 10.13039/501100000921COST (10.13039/501100000921European Cooperation in Science and Technology).

BI is supported by the 10.13039/501100000780European Commission (ERC-CoG 819775, and H2020-HEALTH 945118), by the 10.13039/501100004837Spanish Ministry of Science and Innovation (PID2019‐110369RB‐I00), and by the 10.13039/100012818Comunidad de Madrid (P2022/BMD-7403, RENIM-CM).

CM is funded by the 10.13039/501100001659Deutsche Forschungsgemeinschaft (DFG; SFB-1525 project # 453989101, and Ma 2528/8–1).

RS is supported by 10.13039/501100001659Deutsche Forschungsgemeinschaft (DFG; German Research Foundation) [Project number 268555672—SFB 1213, Project B05].

FS was supported by R01HL46716 and R01HL128831 from the National Heart Lung and Blood Institute.

AMS is supported by the 10.13039/501100000274British Heart Foundation (CH/1999001/11735, RE/18/2/34213) and a Foundation Leducq Transatlantic Network of Excellence award (17CVD04).

## Declaration of competing interest

PF is the founder and CEO of Pharmahungary Group, a group of R&D companies. AMS is an adviser to Forcefield Therapeutics and CYTE – Global Network for Clinical Research and sits on the Board of Heqet Therapeutics.

CM served as an advisor to Amgen, Boehringer Ingelheim, Bristol Myers Squibb, NovoNordisk and Servier and received speaker honoraria from AstraZeneca, Bayer, Bristol Myers Squibb, Boehringer Ingelheim, Berlin Chemie, Novartis and NovoNordisk.

No other author had an interest to declare.

## Data Availability

No data was used for the research described in the article.
